# Death Certification: An Interactive Teaching Session

**DOI:** 10.15766/mep_2374-8265.11296

**Published:** 2023-01-17

**Authors:** James H. Wykowski, Andrew M. Luks, Gabrielle Berger, Desiree A. Marshall

**Affiliations:** 1 Chief Resident, Department of Medicine, University of Washington School of Medicine; 2 Professor, Department of Medicine, Division of Pulmonary, Critical Care, and Sleep Medicine, University of Washington School of Medicine; 3 Clinical Associate Professor, Department of Medicine, University of Washington School of Medicine; 4 Assistant Professor, Department of Laboratory Medicine and Pathology, University of Washington School of Medicine

**Keywords:** Cause of Death, Death Certification, Internal Medicine, Pathology - Clinical

## Abstract

**Introduction:**

Documentation of the cause of death is important for local and national epidemiology as well as for research and public health funding allocation. Despite this, many physicians lack the skills necessary to accurately complete a death certificate.

**Methods:**

We created a 45-minute virtual workshop to improve skills in completing death certificates. Participants examined the role of death certificates in disease epidemiology and resource allocation for research and public health interventions, reviewed the components of a death certificate, and practiced correcting and filling out death certificates from actual patient cases. To assess the workshop, participants completed sample death certificates immediately before and after the workshop for two representative cases.

**Results:**

Thirty-six internal medicine residents (17 PGY 1s, 12 PGY 2s, and seven PGY 3s) completed the workshop. Prior to the workshop, 89% of the sample death certificates contained one or more errors, compared with 46% postworkshop. Major errors, such as incorrect categorization of a cause of death, decreased from 58% preworkshop to 17% postworkshop. Learners expressed discomfort after realizing they had made errors in completing previous death certificates and noted a desire for continuing education and reference materials on this topic.

**Discussion:**

Death certification is a key competency for physicians. Our virtual workshop improved participants’ skills in completing death certificates. Although a significant number of errors remained after the workshop, most of these residual errors were minor and would not affect cause-of-death reporting. The durability of these improvements over time requires further study.

## Educational Objectives

By the end of this activity, learners will be able to:

1. Distinguish between mechanism, proximal cause, and underlying cause of death.
2. Determine the cause of death for two specific clinical scenarios.
3. Identify situations where deaths should be reported to the medical examiner.

## Introduction

Death certification is a required task for most medical providers, with major consequences for patients and their families as well as for the broader society.^1,2^ Completing a death certificate is often the final part of a physician's responsibility to the patient. For families, death certificates are needed to complete funeral arrangements, life insurance, and pension claims, and may also carry sentimental value or perceived stigma.^1,3^ For society, death certificates inform the local, regional, and national epidemiology of many diseases and are essential for setting research priorities and allocating public health resources.^2,4^

Despite the importance of death certificates, prior data suggest that many physicians lack the ability to accurately complete one. A study of 4,800 residents demonstrated that only 23% could accurately complete an example death certificate for a fictional patient, with 45% identifying an incorrect underlying cause of death.^5^ Multiple audits of death certificates confirm these findings: 53%-85% of death certificates contain at least one error, and 23%-60% include an incorrect underlying cause of death.^6–8^

The Centers for Disease Control and Prevention (CDC) has published a death certification handbook for medical providers on its website.^9^ However, this document uses terminology that may be unfamiliar to many clinicians as well as formatting that differs from many hospitals’ death certification templates. Previous studies have shown that training on death certification improves physician confidence and accuracy in completing death certificates.^10,11^ In addition to its handbook, the CDC has created an interactive digital module focused on improving cause-of-death reporting.^12^ A randomized trial of passive module-based training versus interactive training demonstrated that while both improve the accuracy of death certificate completion, the interactive approach yields greater improvement.^11^ A previously published curriculum designed to prepare learners for patient deaths briefly describes the process for completing death certificates but is targeted towards third-year medical students and does not measure learner skills in death certificate completion.^13^

At our institution, internal medicine residents receive only one brief training on death certification prior to commencing clinical rotations. Informal interviews with senior residents have suggested a low level of confidence in this skill. We sought to address this knowledge gap by implementing an interactive teaching session on death certification.^14^ This workshop can be adapted to learners in various stages of their training and in different training specialties.

Nationally, states and local jurisdictions use different systems for reporting and investigating death. These can be broadly divided into either medical examiner systems or coroner systems. Medical examiner is an appointed position held only by medical doctors with subspecialty training in forensic pathology, whereas coroner is an elected position that can be held by any individual, often a nonphysician, who meets county criteria. These distinctions impact who performs autopsies and investigates deaths but do not affect reporting criteria or processes. Thus, our workshop is designed for both systems, with the term *medical examiner/coroner* used throughout. Facilitators can adjust the terminology accordingly.

## Methods

### Facilitator Preparation

Prior to our session, we reviewed recent deaths at our institution. From these, we identified cases with errors on the preliminary cause-of-death statements. We then met with faculty from Autopsy and After Death Services in the Department of Laboratory Medicine and Pathology at our institution to review the internal process for death certification. Faculty from the Department of Pathology also presented the finalized corrected death certificates for the cases during the teaching session.

### Workshop Setting

We conducted a 45-minute virtual workshop in October 2021 during protected didactic time for first-, second-, and third-year residents in internal medicine. First-year residents participating in the workshop had completed four 28-day residency rotations prior to participating in the workshop. We delivered an interactive PowerPoint presentation (Appendix A) using the Zoom platform. Participants were not required to complete any reading or training modules prior to the workshop.

### Workshop Overview

The teaching session comprised three sections. First, we discussed the impact of death certificates for local, regional, and national disease epidemiology. We described the flow of information from a death certificate to local and national agencies. This section lasted about 10 minutes (Appendix A, slides 6–9). We used an example from our local public health agency of deaths from accidental fentanyl overdoses. This example could be applied to other communities or adapted to highlight local public health concerns and the role death certification plays in setting community health priorities.

In the next section, we defined common terms in death certification that might be unfamiliar to many clinicians, such as *mechanism of death* and *underlying cause of death.* This section lasted 20 minutes. We contrasted the typical experience of caring for a dying patient as a clinician with the framework used by pathologists to describe the chain of events leading to a patient's death. Participants were asked to classify commonly listed causes of death as an underlying cause of death, immediate cause of death, or mechanism of death. We then reviewed the template for death certificates and discussed how to apply these terms when completing a death certificate (Appendix A, slides 12–30).

In the third section, we used several cases from our own institution to practice filling out death certificates, first by identifying errors in existing death certificates and then by creating death certificates based on clinical vignettes. This section lasted 15 minutes (Appendix A, slides 32–39). Participants shared their responses to the sample cases using the chat function on Zoom and through open discussion. Our training focused on internal medicine residents, and thus, our cases primarily reflected this patient population. However, the learning objectives from our session are applicable to trainees in multiple specialties. Facilitators can include cases from their own institution and specialty to maximize the relevance of this teaching resource.

We concluded the workshop by discussing the indications for reporting a death to the medical examiner or coroner (Appendix A, slides 41–46). This section included two practice cases, during which participants voted on whether they would report each case using the Zoom polling function.

### Evaluation

We created two sample cases representative of common scenarios for in-hospital deaths: a patient with complex multiorgan failure and a patient with advanced malignancy on a comfort-measures-only pathway (Appendix B). We crafted clinical vignettes, and pathologists wrote example cause-of-death statements, with allowances for variation in practice. Participants completed a practice death certificate for each case immediately before and after the workshop. The same cases were used before and after the workshop. Participants were not given answers to the cases during the workshop. Participants’ responses were anonymously linked using the survey software Catalyst.

We graded responses from 1 to 4 using a standardized scale described in the death certification literature (Table 1).^7^ The full process of grading responses is described in Appendix C. Grades 1 and 2 were pooled as minor errors and included errors such as using an incorrect abbreviation or acronym or an incorrect chain of events. Grade 3 and grade 4 errors were pooled as major errors. Grade 3 errors were defined as including an incorrect immediate cause of death or contributing diagnosis, and grade 4 errors were defined as an incorrect underlying cause of death. After the session, we provided participants with sample answers to the assessment cases and a reference sheet outlining the process for completing a death certificate (Appendix D).

**Table 1.**
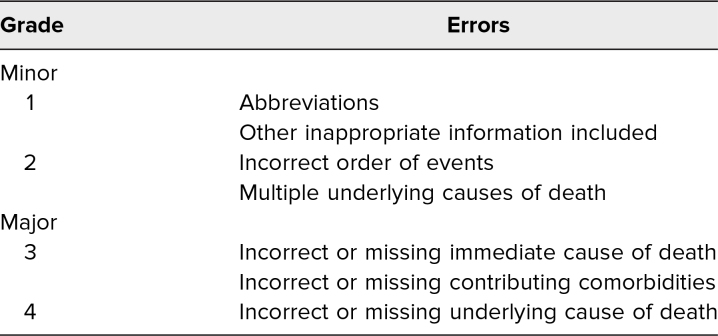
Types of Death Certificate Errors and Corresponding Grade

We categorized participants’ pre- and posttest scores into proficient (no error or highest error grade 1 or 2) and not proficient (highest error grade 3 or 4). We calculated a binomial test of proportions based on the assumption that the proportion of participants scored as proficient did not change after the workshop.^15^

When filling out the sample death certificates for these cases, participants used a template identical to the death note template in our institution's electronic medical record. At our institution, Autopsy and After Death Services uses the information recorded in this death note to create the actual death certificate in an online system, providing suggested edits to cause-of-death language as a part of an internal quality assurance program. Facilitators can include the template used at their own institution so that the evaluation adequately represents the expected workflow for learners completing death certificates in that local context.

After the workshop, we sought feedback from participants on future venues and timing for death certificate education as well as additional learning resources they thought would be helpful.

## Results

Thirty-six residents completed the workshop, including the pre- and postworkshop assessments. Seventeen participants were PGY 1, 12 were PGY 2, and seven were PGY 3. Table 2 describes the frequency of all error types, allowing for multiple error types by a single participant (e.g., a participant who listed an inappropriate underlying cause of death and used abbreviations would be counted as having both grade 1 and grade 4 errors). Table 3 describes the distribution of the single highest error grade for each death certificate. Prior to the workshop, 89% of all sample death certificates contained at least one error, with 58% containing a major error and 20% reporting an incorrect underlying cause of death. Errors in the underlying cause of death were more common in the case describing a patient with multiorgan failure (28%) than in the case describing a patient with advanced cancer on a comfort-measures-only pathway (8%). Prior to the workshop, 49% of participants believed it was necessary to report all in-hospital deaths to the medical examiner.

**Table 2.**
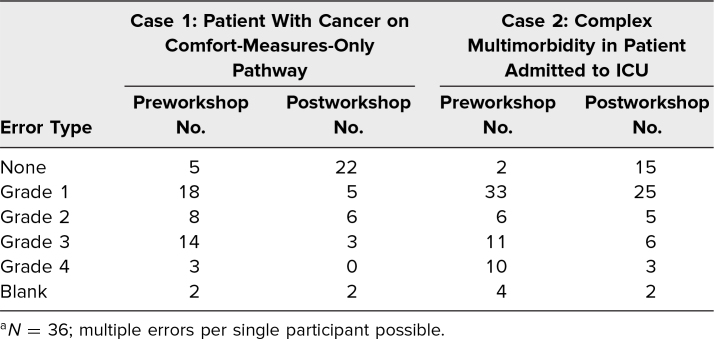
Frequency of Error Types in Pre- and Postworkshop Example Death Certificates^a^

**Table 3.**
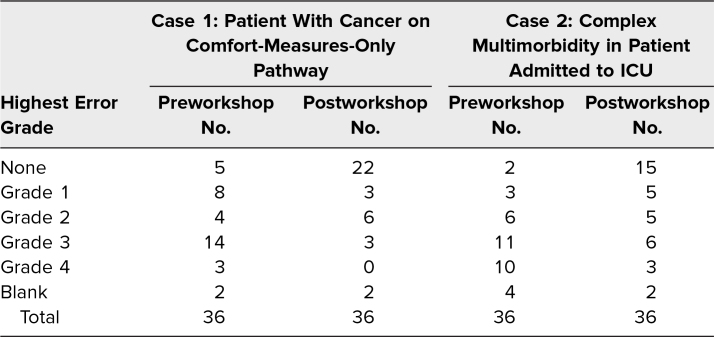
Highest Grade of Error for Pre- and Postworkshop Example Death Certificates

After the workshop, 46% of all death certificates contained at least one error. The highest error grade identified was a minor error in 28% of sample death certificates, a grade 3 error in 13% of sample death certificates, and a grade 4 error in 4% of sample death certificates. Six percent of participants believed that all in-hospital deaths must be reported to the medical examiner. Based on the description of proficient as highest error grade of 0–2, the increase in the proportion of participants categorized as proficient was statistically significant for both cases (Table 4).

**Table 4.**
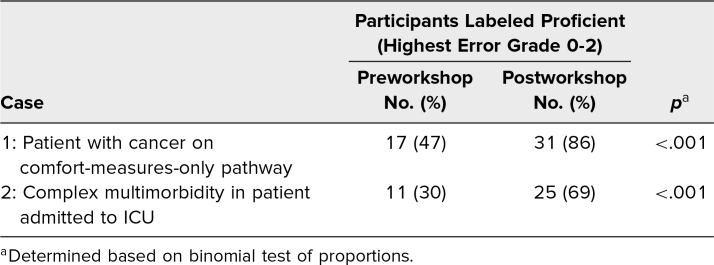
Test of Statistical Significance for Change in Participant Proficiency Pre- and Postworkshop

Using the chat function in Zoom, participants reported significant distress throughout the workshop about their lack of prior knowledge on this topic. They expressed concern that they might have previously completed death certificates incorrectly based on their knowledge gaps. Participants recommended continuing education on death certification. Many stated that the education they had received before starting the intern year was less effective because it took place prior to pronouncing a patient's death and completing the death summary. Instead, they suggested that training occur between the first and second years of residency, when many residents take on a supervisory role for interns pronouncing deaths.

## Discussion

Accurate death certificate completion remains a large knowledge gap for many medical providers and providers-in-training. Our brief virtual workshop improved efficacy at completing death certificates for clinical scenarios commonly encountered by residents at our institution. The strengths of our workshop include the low barrier for participation, ease of use in a digital environment, and adaptability to differing institutional death certification practices.

One reason for our workshop's success was the strong partnership between members of our Internal Medicine Residency Program and Autopsy and After Death Services in the Department of Laboratory Medicine and Pathology. This collaboration clarified the process for cause-of-death reporting, offered access to actual death certificates for use in the workshop, and provided content expertise and review prior to the workshop. We recommend that facilitators partner with their own pathology department and/or medical examiner's office to ensure the accuracy of content about local reporting practices.

Despite the improvement shown by participants in our workshop, nearly half of death certificates still contained at least one error afterward. However, most of these errors were minor, such as continuing to use inappropriate abbreviations (e.g., CHF instead of congestive heart failure) or using an incorrect chain of events when completing the death certificates. In general, minor errors do not significantly impact health statistics, and at our institution, After Death Services corrects minor errors prior to death certificate finalization. Thus, while a 46% error rate postworkshop was not ideal, we do not think this reflected negatively on the workshop's overall efficacy. The reduction in high error grades was significant and has important implications for real-life clinical practice. Additionally, because one of the criteria for a grade 1 error was use of abbreviations and one of the cases used a patient with chronic obstructive pulmonary disease, which is often abbreviated as COPD in clinical practice, participants may have been particularly vulnerable to committing grade 1 errors when completing the second case.

Our evaluation is limited by the lack of longitudinal follow-up to assess the durability of knowledge gained through the workshop. We attempted to decrease knowledge decay by providing participants with a digital handout for use as a reference when completing death certificates in the future (Appendix D), but we have no data on the utility of this intervention. Another institutional intervention to support long-term retention of this material could be to ensure adequate training of attending physicians, who could then provide ongoing real-time feedback to trainees after they receive a draft death certificate. This type of longitudinal intervention would both prevent knowledge decay and promote a culture of ongoing improvement and death certificate education. A more idealized evaluation would assess actual death certificates filled out by trainees after the workshop and compare error rates with those of nonparticipants. Additionally, our evaluation would have been strengthened by systematically describing participant comfort with filling out death certificates before and after the workshop. Comparing subjective comfort with objective performance would help us better understand gaps between residents’ perceived and actual ability to fill out death certificates. It would also be possible to reexamine learner performance 3–6 months after the workshop to assess for retention of skills. Finally, our evaluation could have been strengthened by quantifying participants’ experience with death certificates prior to the workshop to better understand who most benefited from the workshop.

Accurate death certificate completion is essential to local and national epidemiology. Education on this topic should be mandatory for all medical provider trainees. Our brief virtual workshop on death certification improved trainees’ skills at our institution and is easily adaptable to other institutions.

## Appendices


Death Certification Interactive Session.pptxExample Cases.docxRubric for Grading Cases.docxTake-home Handout for Participants.docx
*All appendices are peer reviewed as integral parts of the Original Publication.*
