# Supplementary material for: Death Certification: An Interactive Teaching Session
Source: MedEdPORTAL. 2023 Jan 17;19:11296. doi: 10.15766/mep_2374-8265.11296 (PMC9842806; doi:10.15766/mep_2374-8265.11296)
Supplement: Supplementary file 1 — Death Certification Interactive Session.pptxExample Cases.docxRubric for Grading Cases.docxTake-home Handout for Participants.docx [file mep_2374-8265.11296-s001.zip › C. Rubric for Grading Cases.docx]

Death Certification: An Interactive Teaching Session

Appendix C: Rubric for grading evaluation cases

**Section 1:General Instructions**

Cases should be evaluated based on the rubric listed below. When errors are present for multiple grades, assign the highest-grade error (e.g., certificate with abbreviations used and incorrect underlying cause of death would be grade 4). Examples of correct and incorrect death certificates are provided below.

Death Certificate Error Grades

| Grade | Error(s) |
| --- | --- |
| One | Abbreviations  Other inappropriate information included |
| Two | Incorrect order of events  Multiple underlying causes of death |
| Three | Incorrect or missing immediate cause of death  Incorrect or missing contributing comorbidities |
| Four | Incorrect or missing immediate cause of death |

**Section 2: Example Certificates Graded**

**Case 1: Case Description**

A 58-year-old-woman with hypertension, hyperlipidemia, and breast cancer with known peritoneal carcinomatosis is directly admitted from the oncology clinic for intractable pain. A CT scan of the abdomen and pelvis demonstrates her known metastatic disease. After talking with her primary oncologist and the Palliative Care team, she transitions to comfort measures only with the aim of going home on hospice care. Before she can be discharged, she dies in the hospital on a hydromorphone infusion.

**Correct Examples:**

Example 1:

Immediate cause of death:

Next oldest condition:

Next oldest condition:

Underlying cause of death: Metastatic breast cancer

Significant comorbidities contributing to death:

Example 2:

Immediate Cause of Death: Presumed hypoxemic respiratory failure

Next oldest condition: Cancer-related pain

Next oldest condition: Peritoneal carcinomatosis

Underlying cause of death: Metastatic breast cancer

Significant comorbidities contributing to death:

Discussion: These examples represent two different approaches to the death certificate in this patient. Without additional data to understand what caused her death, it is reasonable to only list her underlying diagnosis of breast cancer. This could be listed on the top “Preliminary Cause of Death 1” line as well. Other clinicians may want to include more details on the death certificate, which is permissible if the chain of events is described correctly. With this clinical picture, presuming the mechanism of death to be hypoxemia is reasonable with the “more likely than not” standard.

**Case 1 Incorrect Examples:**

Example Certificate One:

Immediate cause of death

Next oldest condition:

Next oldest condition:

Underlying cause of death: Breast ca, abdominal mets

Significant comorbidities contributing to death:

Discussion: This example contains multiple errors. There are multiple diagnoses listed as the underlying cause of death, a Grade 2 error. The abbreviations “ca” and “mets” are not allowed, a Grade 1 error. The higher-grade error of multiple underlying causes of death takes precedence, and so this is a Grade 2 error.

Example Certificate Two:

Immediate cause of death: Hypoxemic respiratory failure

Next oldest condition:

Next oldest condition:

Underlying cause of death:

Significant comorbidities contributing to death: Breast cancer

Discussion: This example represents a Grade 4 error, as there is no underlying cause of death identified. The immediate cause of death, hypoxemic respiratory failure, is a mechanism, not a cause of death, and there is no additional supporting information.

**Case 2: Case Description**

A 52-year-old man with chronic obstructive pulmonary disease is admitted to the ICU with hypoxemic respiratory failure due to COVID-19 requiring oxygen via high-flow nasal cannula. On hospital day 3, he develops worsening chest pain and hypoxemia, is found to have a new segmental pulmonary embolism and is started on anticoagulation. Despite this intervention, he develops progressive hypoxemia and suffers a cardiac arrest. After 30 minutes of advanced cardiac life support measures, return of spontaneous circulation is never achieved and he is declared dead.

Case 2: Correct Example

Immediate cause of death: Pulmonary embolism

Next oldest condition:

Next oldest condition:

Underlying cause of death: COVID-19 pneumonia

Significant comorbidities contributing to death: Chronic obstructive pulmonary disease

Discussion: Participants could choose to also include additional mechanisms in the subsequent preliminary cause of death lines, including cardiac arrest, obstructive shock, or hypoxemic respiratory failure, provided they still include COVID-19 pneumonia as the underlying cause of death and pulmonary embolism as preliminary cause of death 1, with COPD as a significant comorbidity. A logical chain of events must be established.

Case 2: Incorrect Example 1

Immediate cause of death: Pulmonary embolism

Next oldest condition:

Next oldest condition:

Underlying cause of death: COVID-19 pneumonia

Significant comorbidities contributing to death:

Discussion: This represents a grade 3 error, due to omission of COPD as a significant comorbidity.
