# Supplementary material for: Death Certification: An Interactive Teaching Session
Source: MedEdPORTAL. 2023 Jan 17;19:11296. doi: 10.15766/mep_2374-8265.11296 (PMC9842806; doi:10.15766/mep_2374-8265.11296)
Supplement: Supplementary file 1 — Death Certification Interactive Session.pptxExample Cases.docxRubric for Grading Cases.docxTake-home Handout for Participants.docx [file mep_2374-8265.11296-s001.zip › D. Take-home Handout for Participants.docx]

**Appendix D: Tips on Completing Death Notes and Calling the Medical Examiner**

**Why Death Certification Matters**

The specified cause(s) of death is(are) important for several reasons:

• **Health statistics**: local, national and international health statistics are used to set public health goals and funding priorities for research and public health interventions.

• **Legal implications**: criminal and civil matters may arise after patient deaths and the cause of death can play a role in these proceedings.

• **Insurance and other claims**: the ability to collect on life insurance policies or resolve issues related to workplace injuries may be affected by the specified cause of death.

• **Family peace of mind**: listing particular causes of death may cause family distress, particularly in cases where the cause is a potentially stigmatizing condition (e.g., drug overdose). Always be accurate, but if a stigmatizing condition did not definitely contribute – omit. Consider using the term WHO class III obesity rather than “morbid” obesity.

**Categories for Causes of Death**

The causes of death can be broken down into one of three categories. Understanding the distinction between these categories is key to filling out the death note accurately.

| **Category** | **Underlying** | **Immediate** | **Mechanism** |
| --- | --- | --- | --- |
| **Definition** | Disease that started the lethal chain of events | Complication linked to the underlying cause | Physiological / biochemical effects of the immediate cause |
| **Examples** | Alcohol use disorder  Alzheimer’s dementia  Atherosclerosis  Coronary artery disease  Chronic kidney disease  Cancer  COVID-19  Diabetes mellitus  Hepatitis C  Motor vehicle collision | Aspiration pneumonia  COPD exacerbation  Gastrointestinal bleeding  Hepatorenal syndrome  Ischemic stroke  Meningitis  Myocardial infarction  Pulmonary embolism  Sepsis | Anoxic brain injury  ARDS  Cardiac tamponade  Cerebral herniation  DIC  Hypothermia  Pulmonary edema  Septic shock |

**Filling in the Death Note**

Providers are prompted to complete a table as part of the death note in the electronic medical record:

| **Presumed cause of death** | | |
| --- | --- | --- |
|  | **Cause** | **Approximate Duration** |
| **Preliminary cause of death 1** |  |  |
| **Preliminary cause of death 2** |  |  |
| **Preliminary cause of death 3** |  |  |
| **Underlying cause of death** |  |  |
| **Significant comorbidities contributing to death** |  |  |

The following are key factors to consider when completing the death certificate:

• Start with the underlying cause of death first and be specific.

• Working upwards from the underlying cause, move to the rows for preliminary causes to list diagnoses that fall in the categories of “immediate causes” or “mechanisms” of death.

• If there is a chain of events, the immediate causes and mechanisms should follow a causal path starting with the initial problem. For example, acute myeloid leukemia, then bacterial pneumonia, then septic shock

• Many commonly encountered chronic conditions still have underlying etiologies that should be specified when known, e.g., cirrhosis due to hepatitis c, ESRD due to Type 2 Diabetes

• In rare situations, the underlying cause may be the only thing that goes in the table. In such cases, place it in the first row of “Preliminary cause of death.” An example is a patient who dies from advanced lung cancer.

• Acknowledge uncertainty by using terms such as, “suspected” or “likely.”

• Never use abbreviations or acronyms

• Mechanisms such “cardiac arrest,” “respiratory failure,” or “brain death” should never go on the death certificate without an underlying cause. Do not include “comfort care” on the death certificate.

• The row for “significant comorbidities contributing to death” is not a problem list. Instead, list chronic conditions or in-hospital complications that affected the severity of the underlying problem and meaningfully contributed to death. For example, if a patient with COPD dies from COVID-19, COVID-19 would be listed as the cause of death while COPD would be listed under significant comorbidities.

**When To Call the Medical Examiner/Coroner**

Providers are *not* required to call the Medical Examiner/Coroner after all deaths. These entities will generally decline involvement for deaths considered to be natural but will assume jurisdiction for deaths due to accidents, suspected suicides, homicides, and possibly those for which the cause of death is unknown. The following is a practical list of situations in which providers should report a death to the Medical Examiner’s Office:

• Death caused by injury or violence, regardless of how long ago the injury occurred

• Death caused in part or entirely by unlawful or unnatural means

• Death related to an undiagnosed toxin or infectious agent

• Suspected drug overdose or poisoning

• Deaths related to a complication of a procedure or treatment

• Patients who were transferred from jail, prison or other non-medical institution

• Work-related deaths

• During or within 42 days of pregnancy

• Unexpected death of an apparently healthy person and without medical attendance within 36 hours of death

• Any suspicious death

If unsure of how to proceed, providers should feel empowered to call the Medical Examiner or Coroner. In cases where they are likely to take jurisdiction (e.g., overdose, trauma), avoid asking legal next-of-kin permission for autopsy, as this decision will ultimately be made by the Medical Examiner’s office (consent not required for cases legally under ME/coroner jurisdiction).
