# Supplementary material for: Death Certification: An Interactive Teaching Session
Source: MedEdPORTAL. 2023 Jan 17;19:11296. doi: 10.15766/mep_2374-8265.11296 (PMC9842806; doi:10.15766/mep_2374-8265.11296)
Supplement: Supplementary file 1 — Death Certification Interactive Session.pptxExample Cases.docxRubric for Grading Cases.docxTake-home Handout for Participants.docx [file mep_2374-8265.11296-s001.zip › A. Death Certification Interactive Session.pptx]

## Slide 1
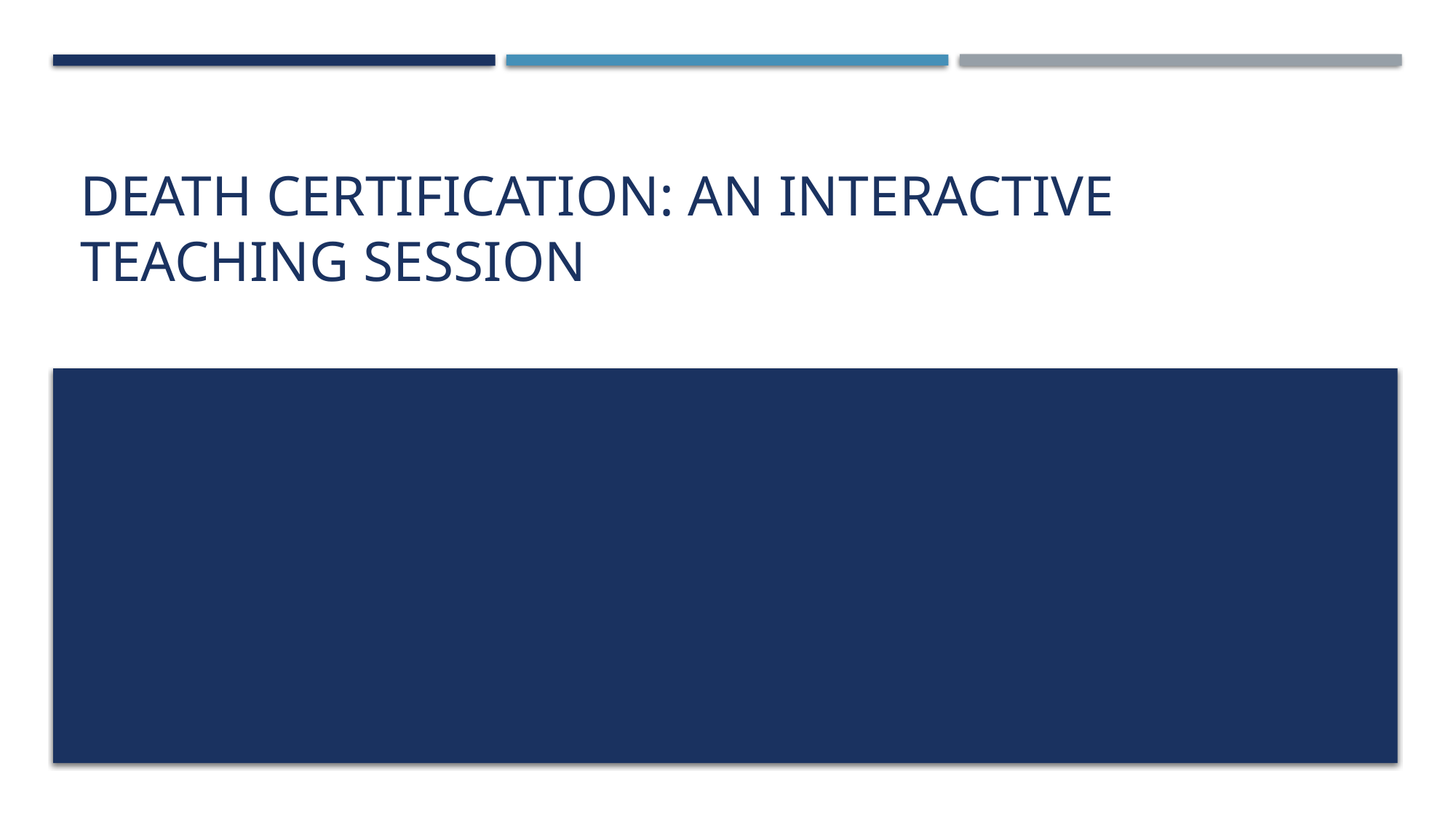

# DEAth Certification: an interactive Teaching Session

## Slide 2
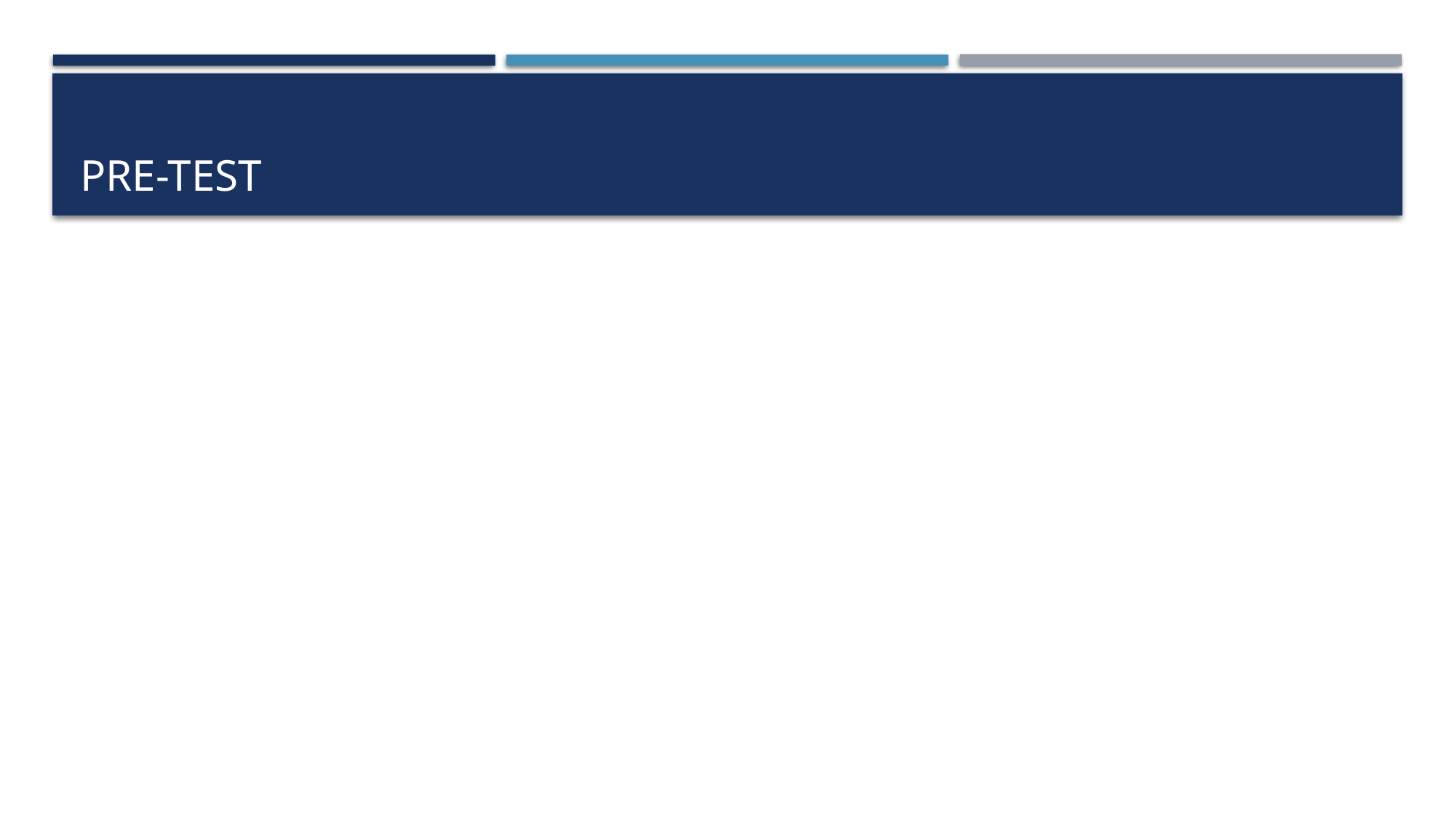

# Pre-Test

## Slide 3
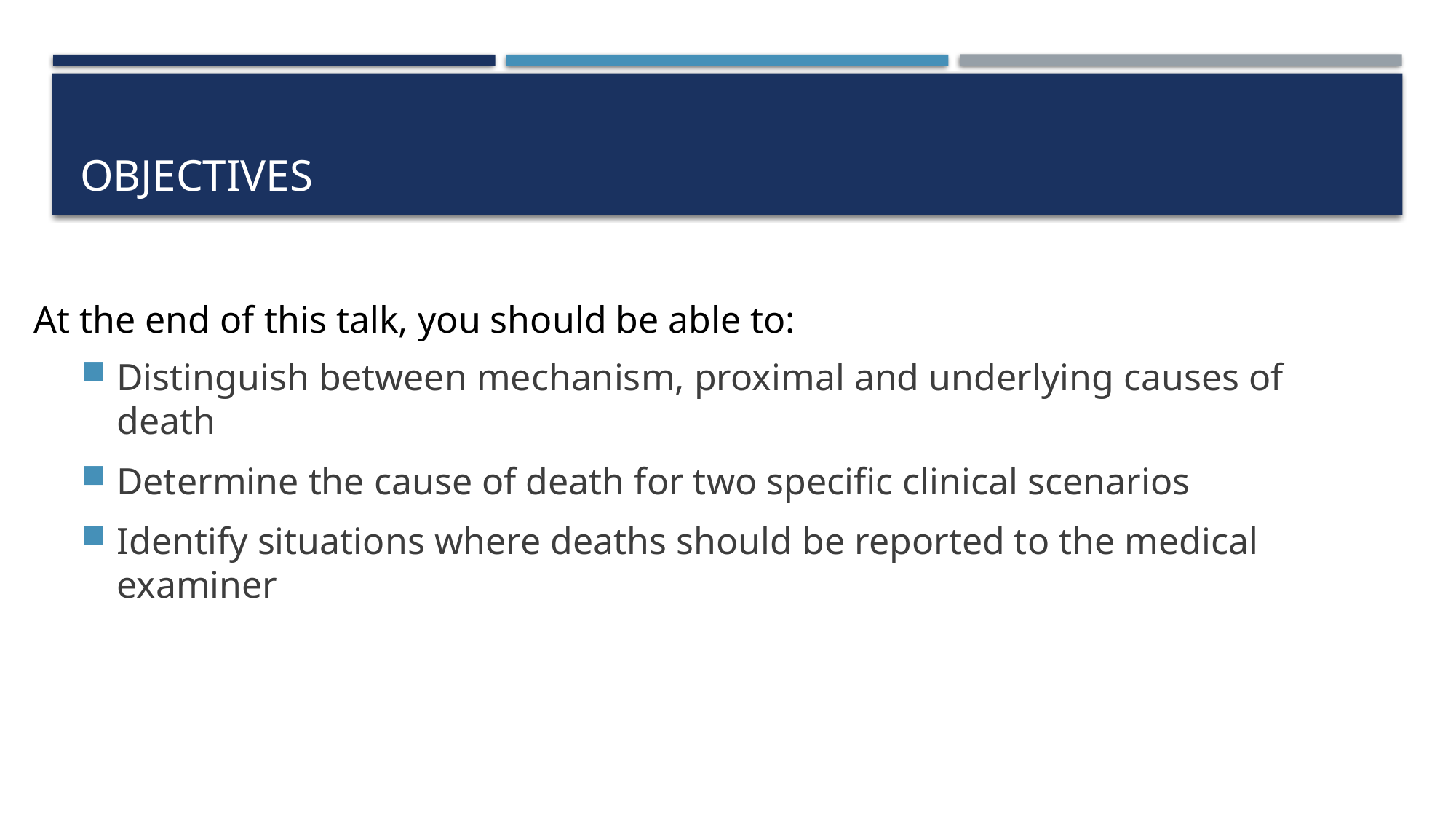

# Objectives
Distinguish between mechanism, proximal and underlying causes of death
Determine the cause of death for two specific clinical scenarios
Identify situations where deaths should be reported to the medical examiner
At the end of this talk, you should be able to:

## Slide 4
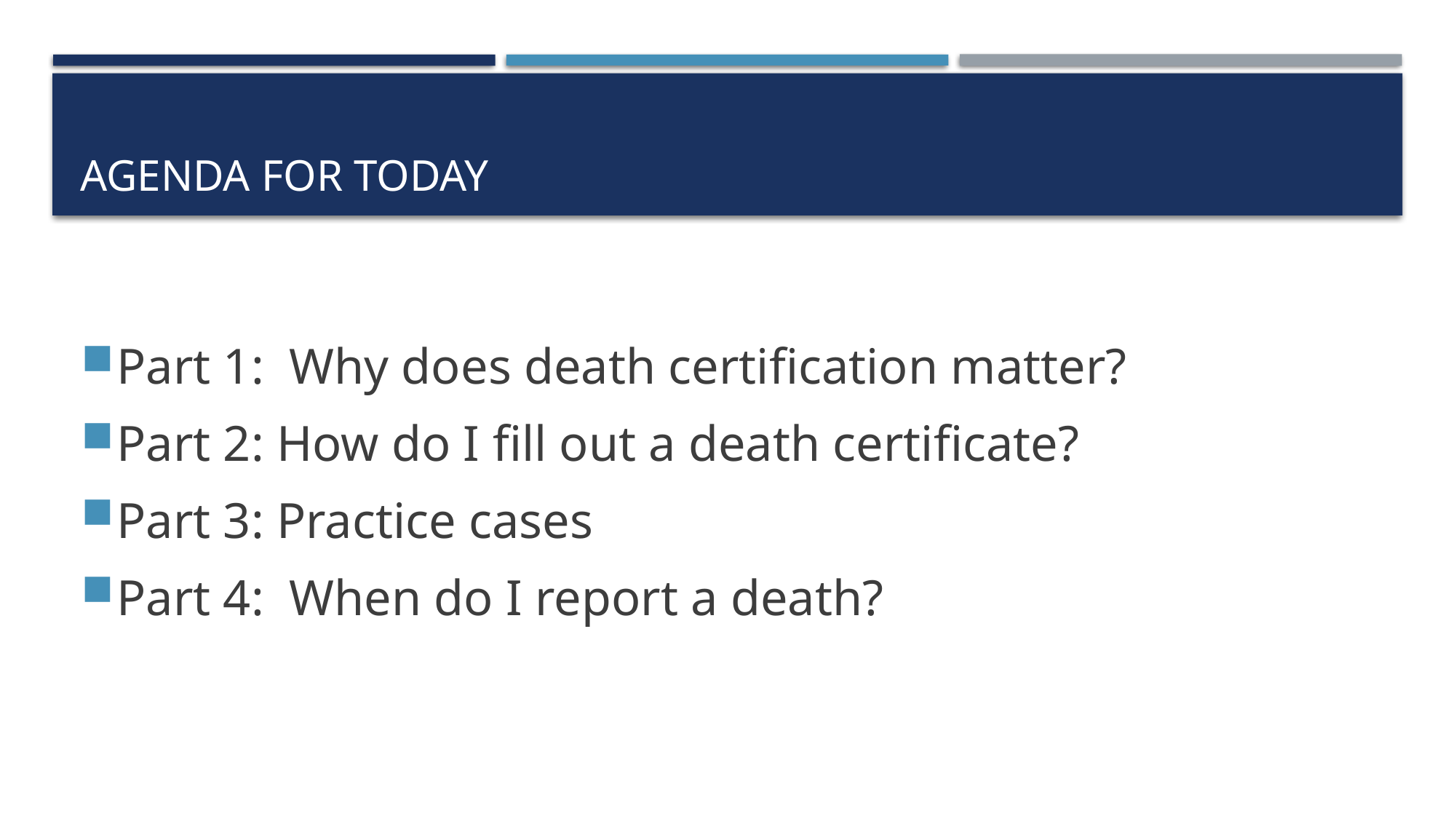

# Agenda for today
Part 1: Why does death certification matter?
Part 2: How do I fill out a death certificate?
Part 3: Practice cases
Part 4: When do I report a death?

## Slide 5
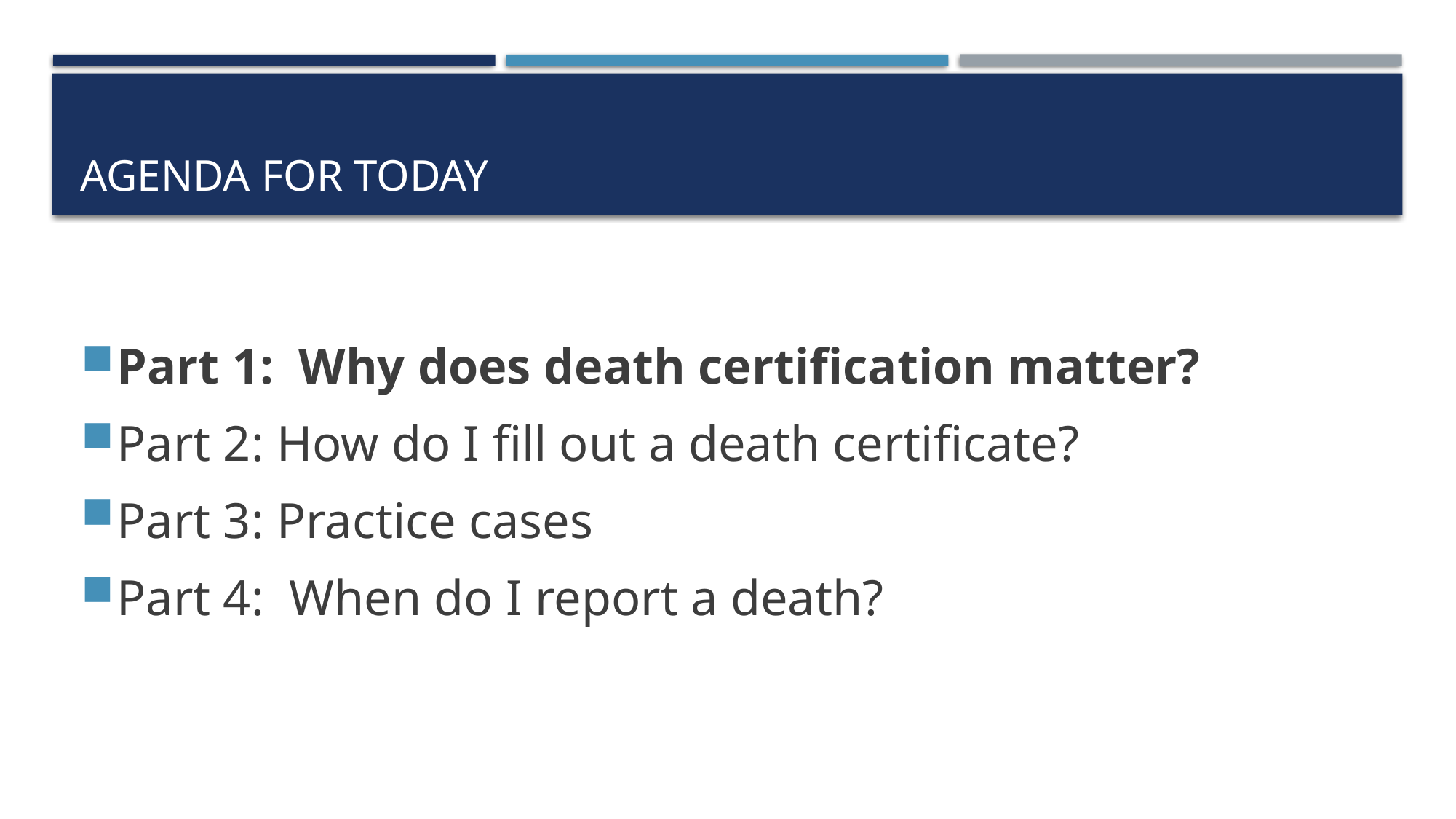

# Agenda for today
Part 1: Why does death certification matter?
Part 2: How do I fill out a death certificate?
Part 3: Practice cases
Part 4: When do I report a death?

## Slide 6
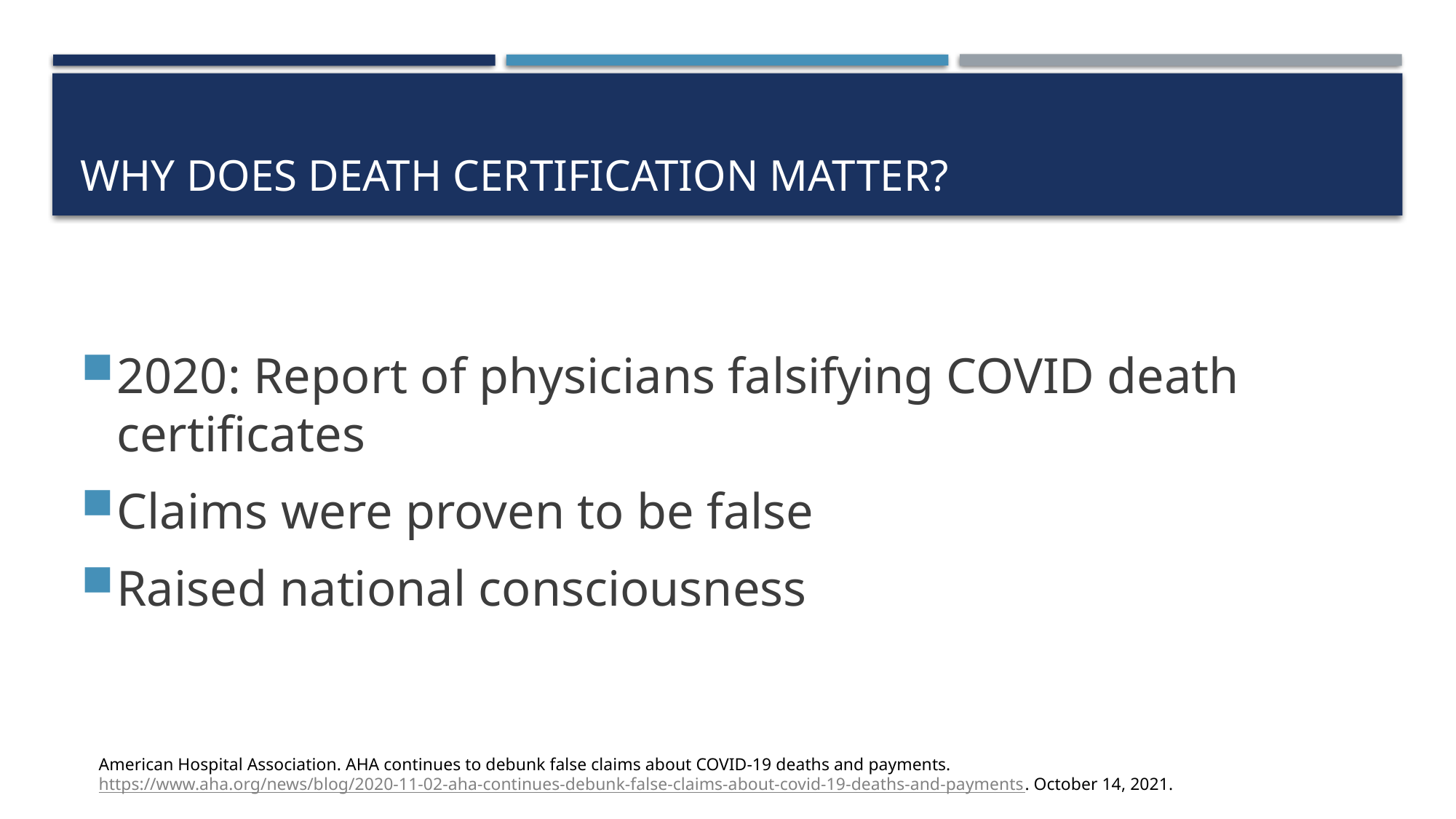

# Why does death certification matter?
2020: Report of physicians falsifying COVID death certificates
Claims were proven to be false
Raised national consciousness
American Hospital Association. AHA continues to debunk false claims about COVID-19 deaths and payments. https://www.aha.org/news/blog/2020-11-02-aha-continues-debunk-false-claims-about-covid-19-deaths-and-payments. October 14, 2021.

## Slide 7
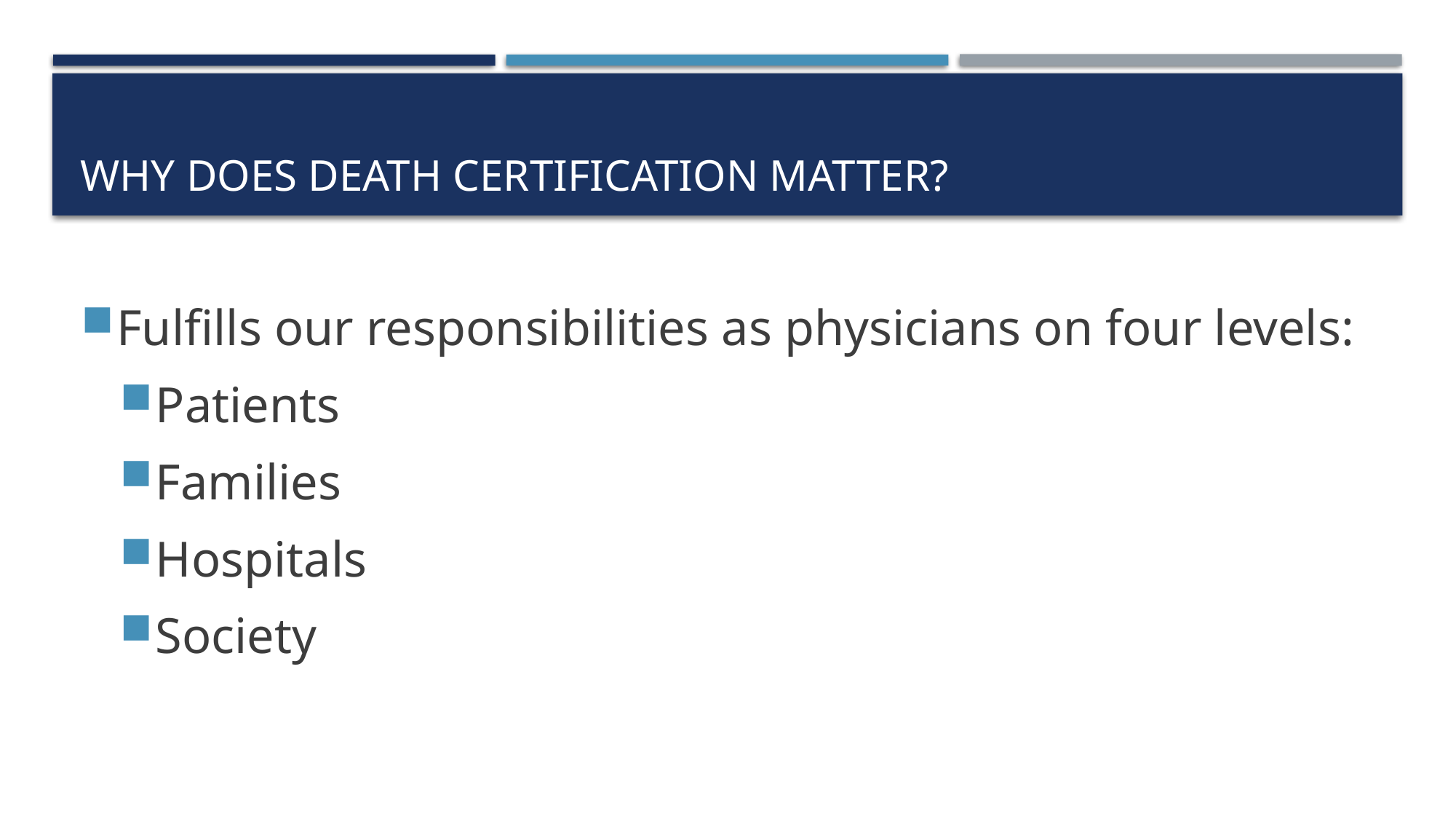

# Why does death certification matter?
Fulfills our responsibilities as physicians on four levels:
Patients
Families
Hospitals
Society

## Slide 8
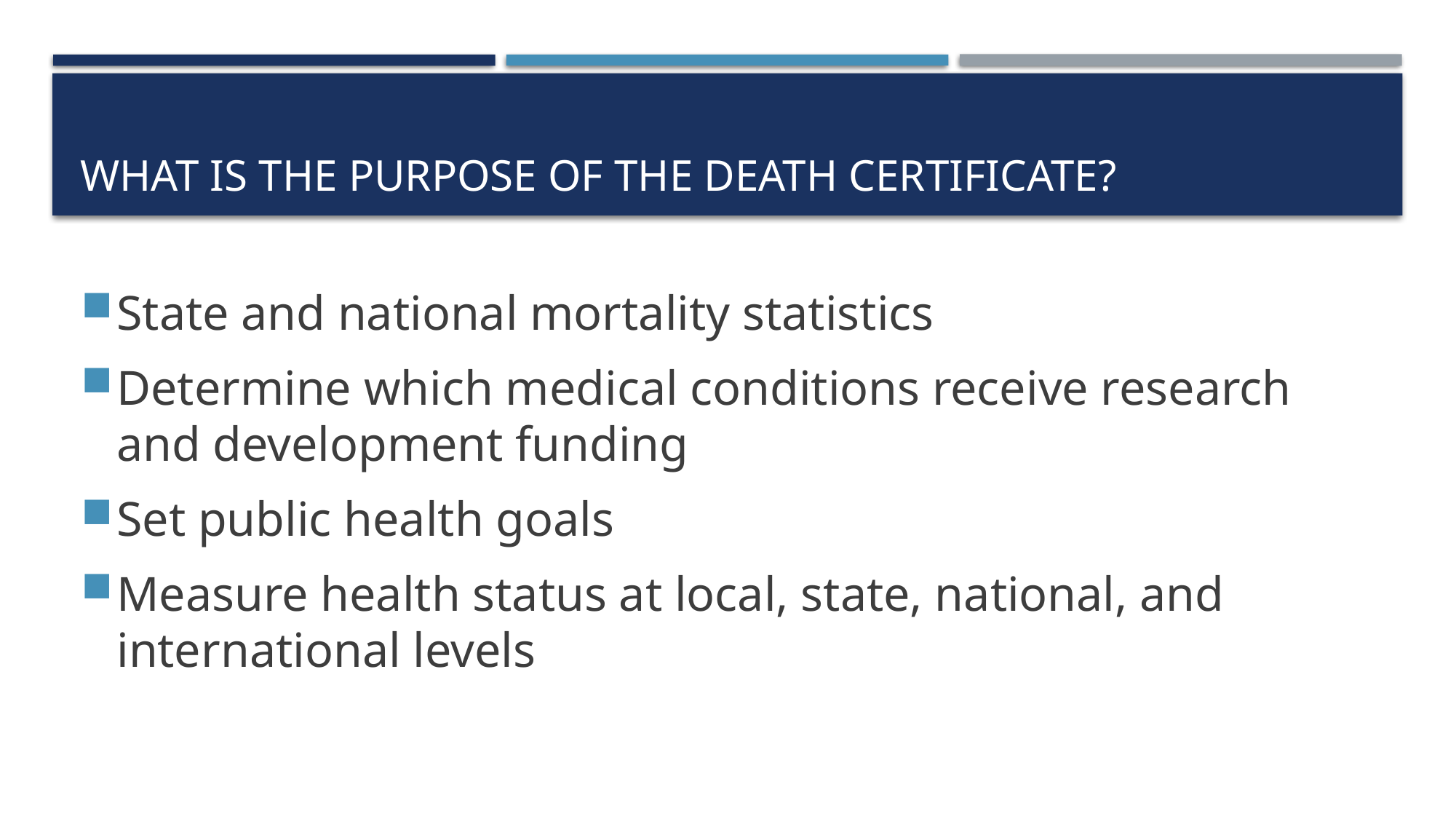

# What is the purpose of the death certificate?
State and national mortality statistics
Determine which medical conditions receive research and development funding
Set public health goals
Measure health status at local, state, national, and international levels

## Slide 9
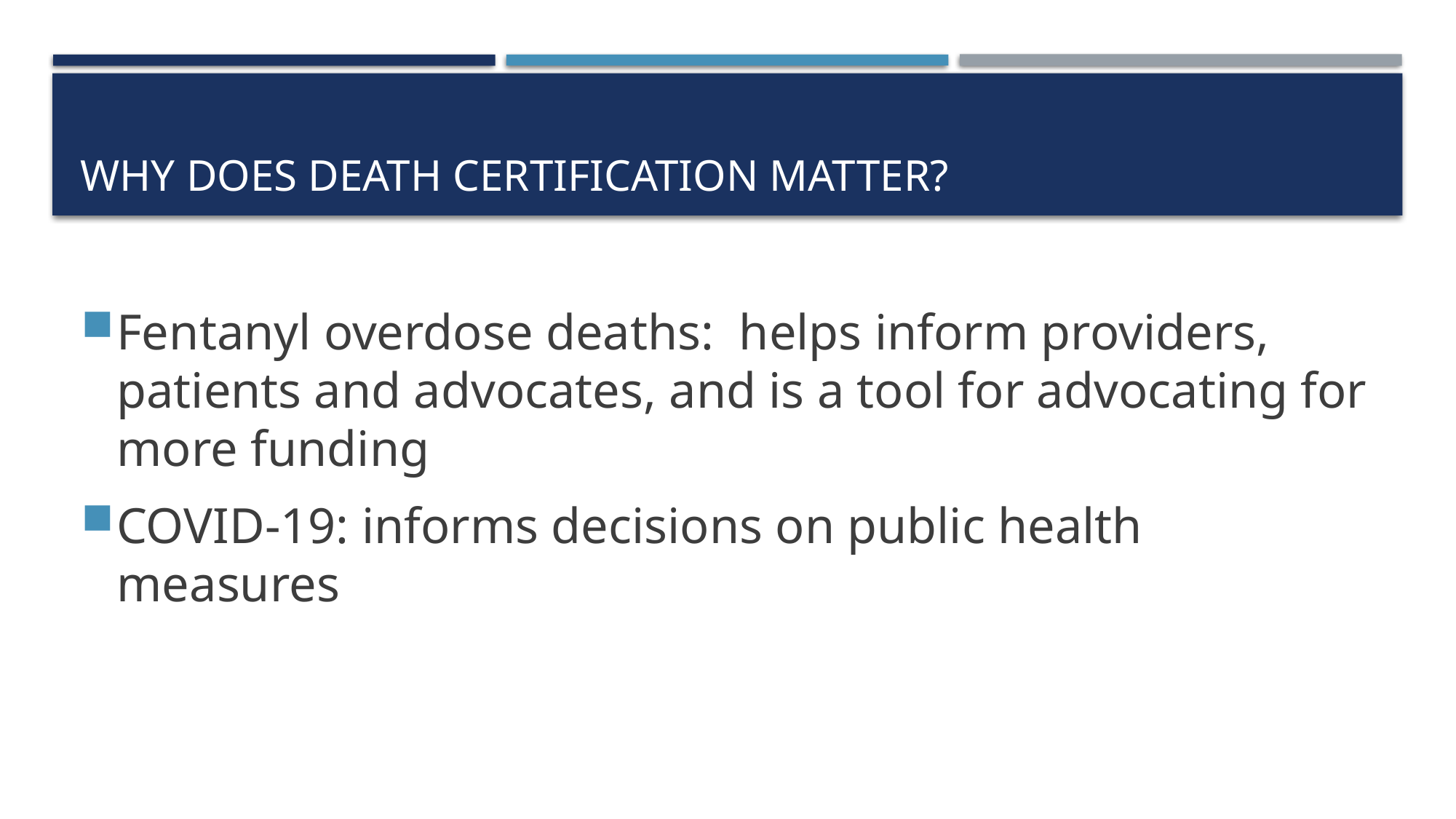

# Why does death certification matter?
Fentanyl overdose deaths: helps inform providers, patients and advocates, and is a tool for advocating for more funding
COVID-19: informs decisions on public health measures

## Slide 10
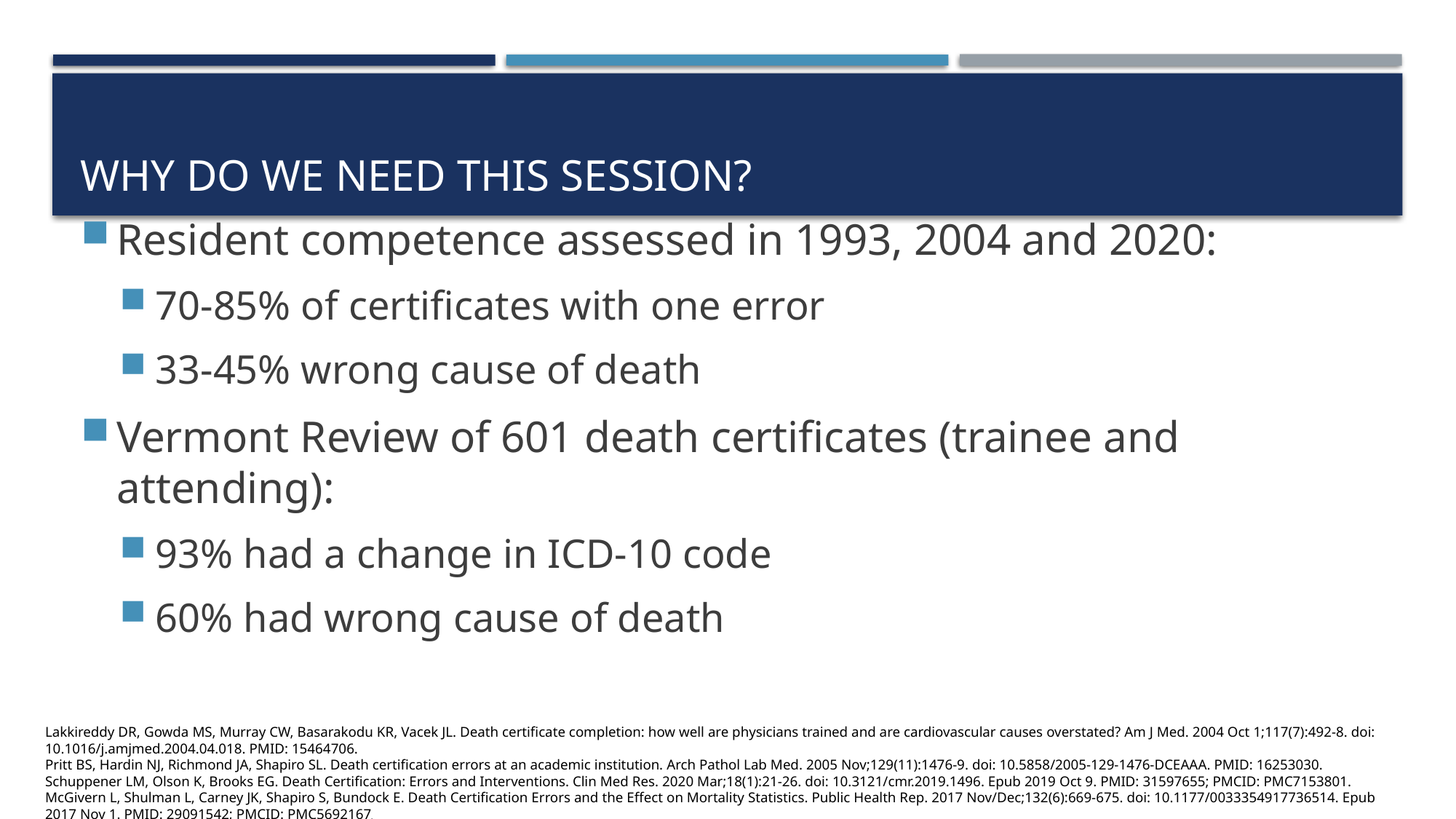

# Why do we need this session?
Resident competence assessed in 1993, 2004 and 2020:
70-85% of certificates with one error
33-45% wrong cause of death
Vermont Review of 601 death certificates (trainee and attending):
93% had a change in ICD-10 code
60% had wrong cause of death
Lakkireddy DR, Gowda MS, Murray CW, Basarakodu KR, Vacek JL. Death certificate completion: how well are physicians trained and are cardiovascular causes overstated? Am J Med. 2004 Oct 1;117(7):492-8. doi: 10.1016/j.amjmed.2004.04.018. PMID: 15464706.
Pritt BS, Hardin NJ, Richmond JA, Shapiro SL. Death certification errors at an academic institution. Arch Pathol Lab Med. 2005 Nov;129(11):1476-9. doi: 10.5858/2005-129-1476-DCEAAA. PMID: 16253030.
Schuppener LM, Olson K, Brooks EG. Death Certification: Errors and Interventions. Clin Med Res. 2020 Mar;18(1):21-26. doi: 10.3121/cmr.2019.1496. Epub 2019 Oct 9. PMID: 31597655; PMCID: PMC7153801.
McGivern L, Shulman L, Carney JK, Shapiro S, Bundock E. Death Certification Errors and the Effect on Mortality Statistics. Public Health Rep. 2017 Nov/Dec;132(6):669-675. doi: 10.1177/0033354917736514. Epub 2017 Nov 1. PMID: 29091542; PMCID: PMC5692167.

## Slide 11
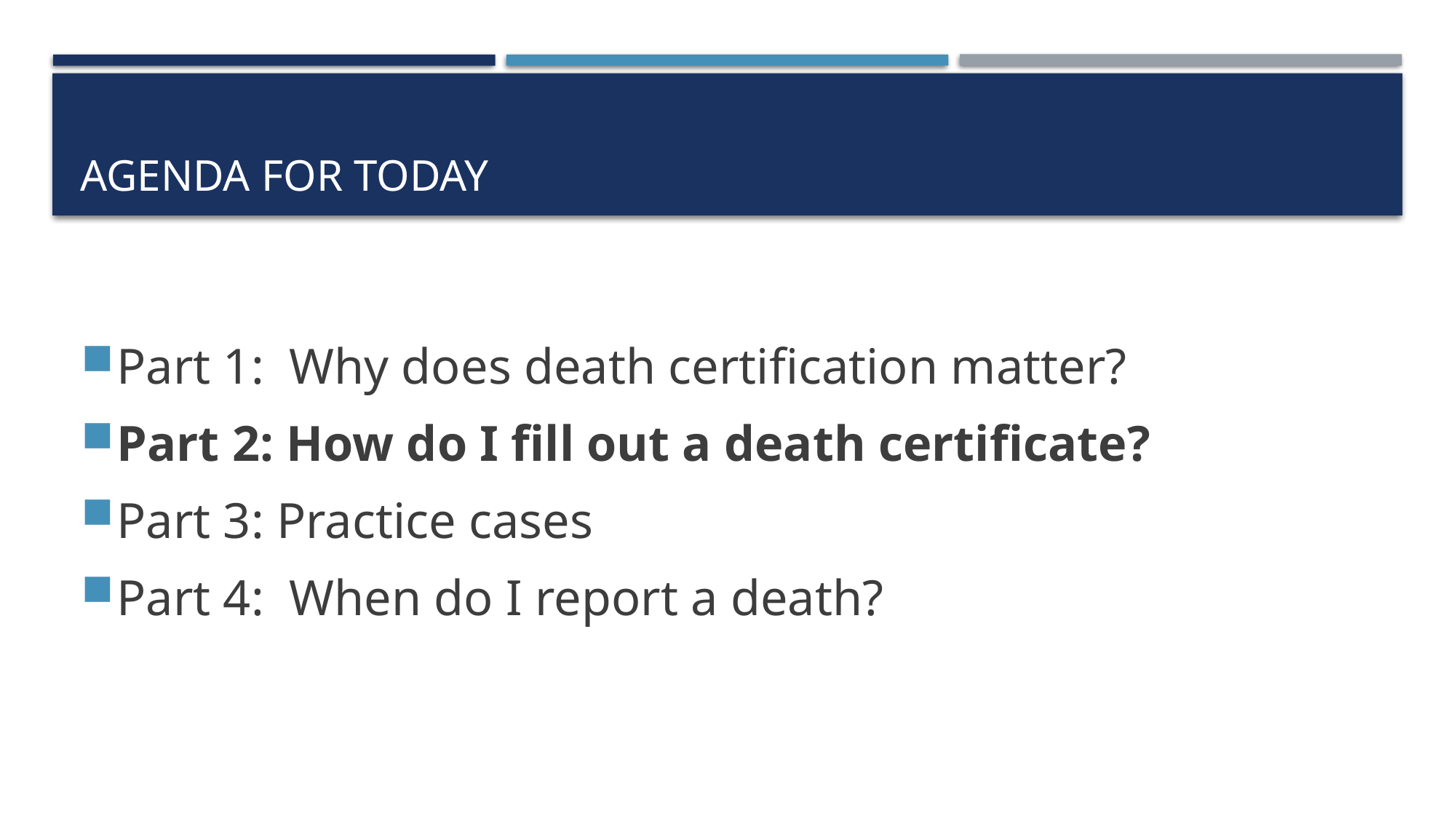

# Agenda for today
Part 1: Why does death certification matter?
Part 2: How do I fill out a death certificate?
Part 3: Practice cases
Part 4: When do I report a death?

## Slide 12
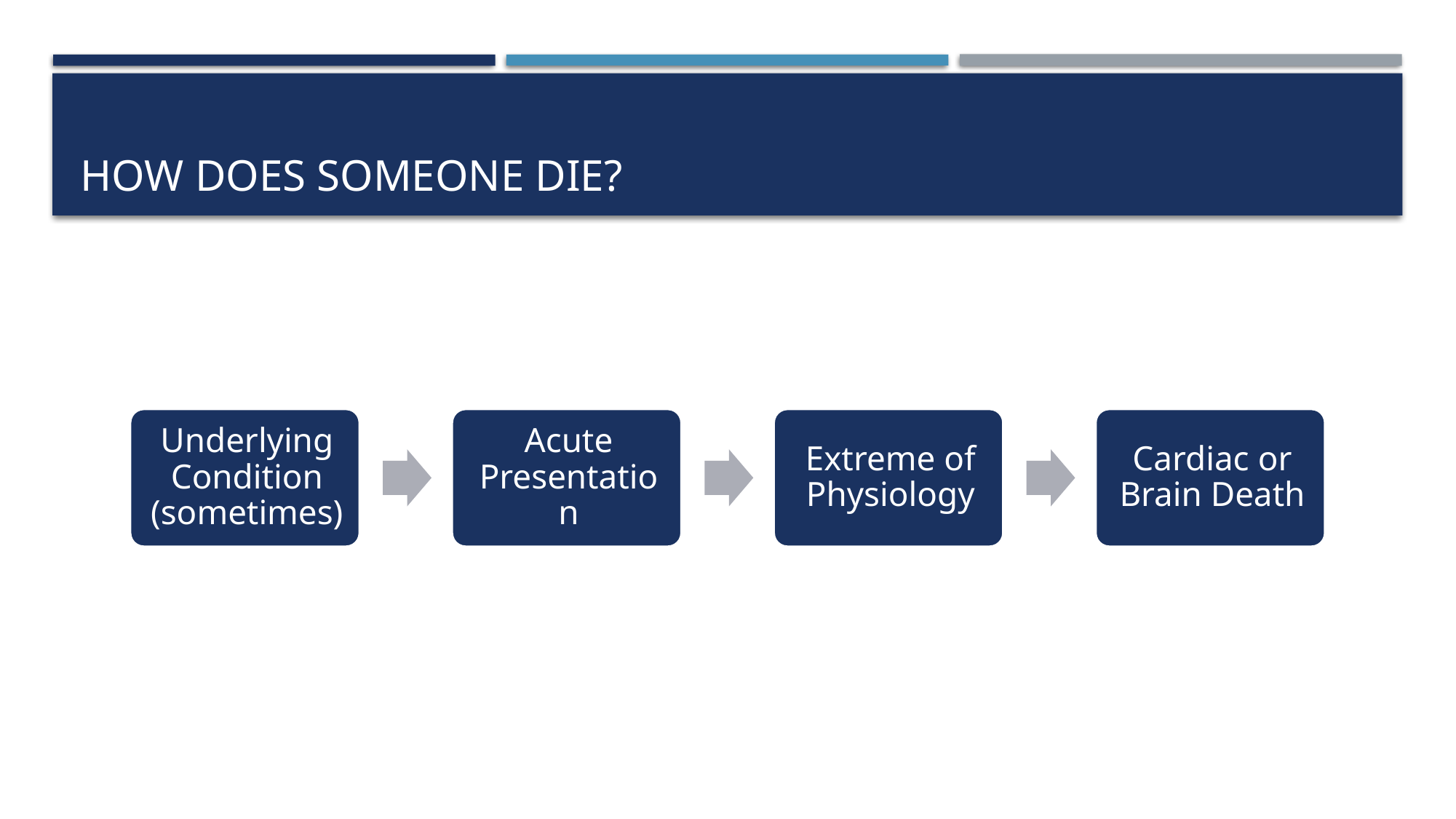

# How does someone die?

## Slide 13
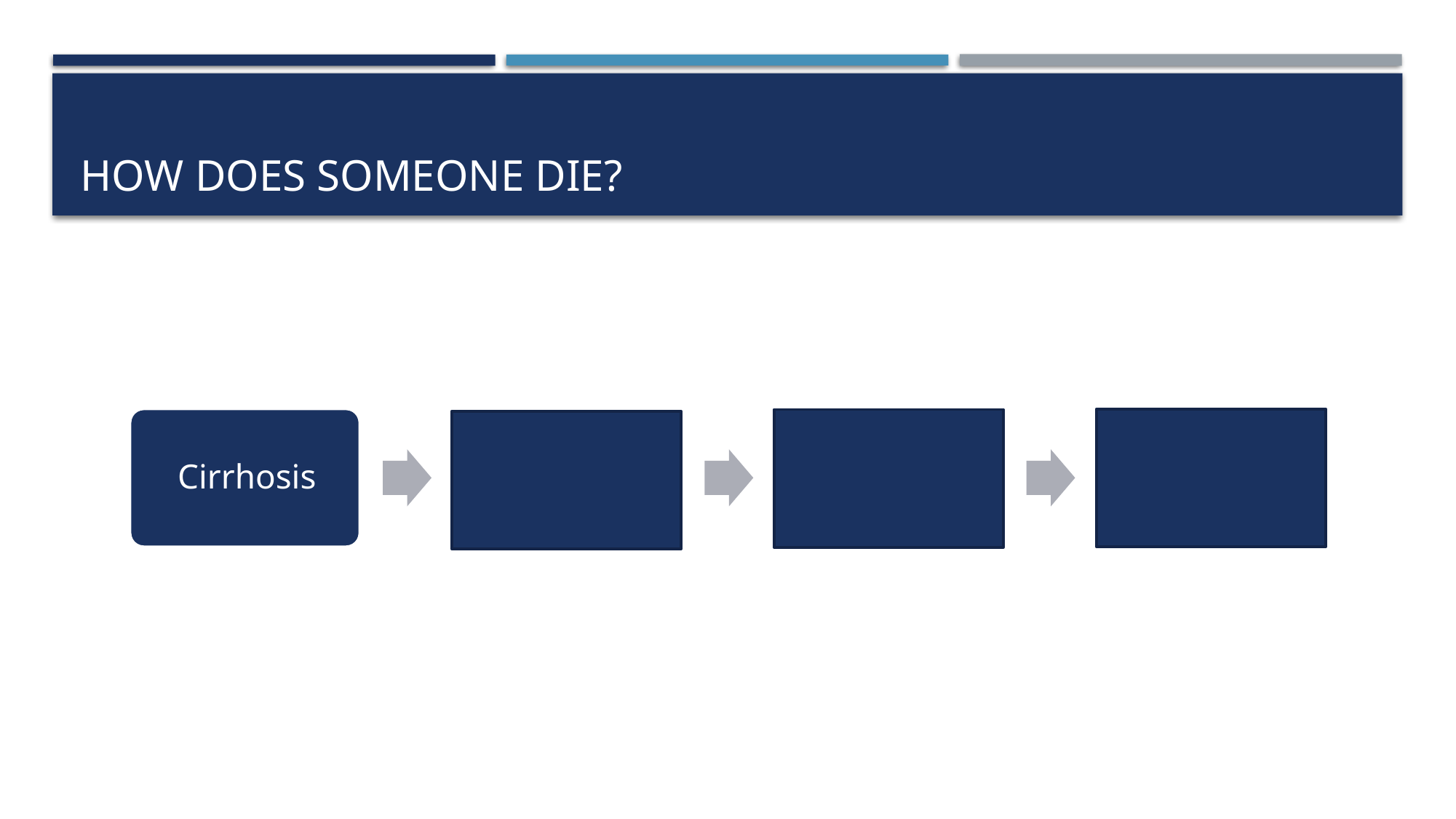

# How does someone die?

## Slide 14
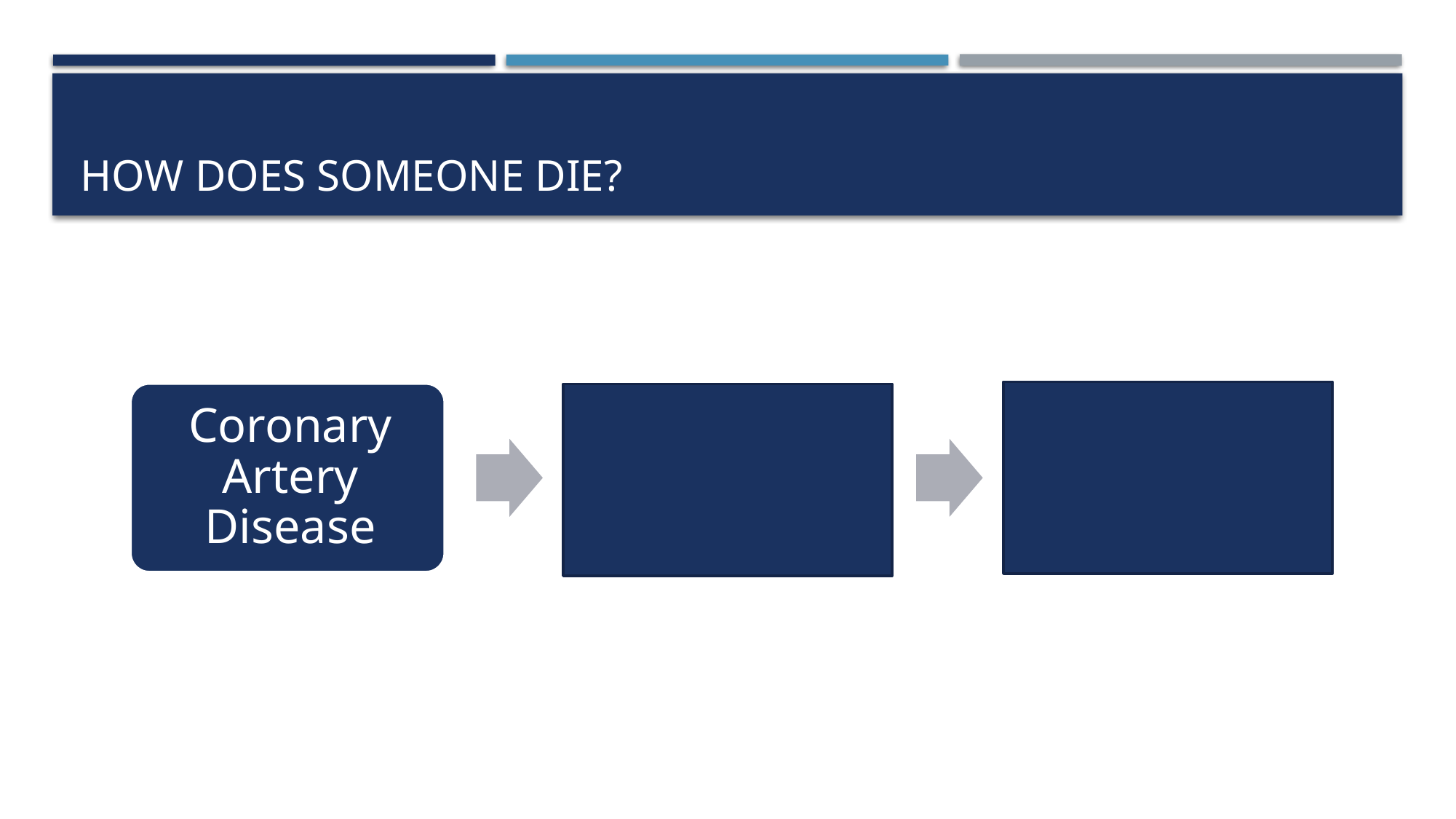

# How does someone die?

## Slide 15
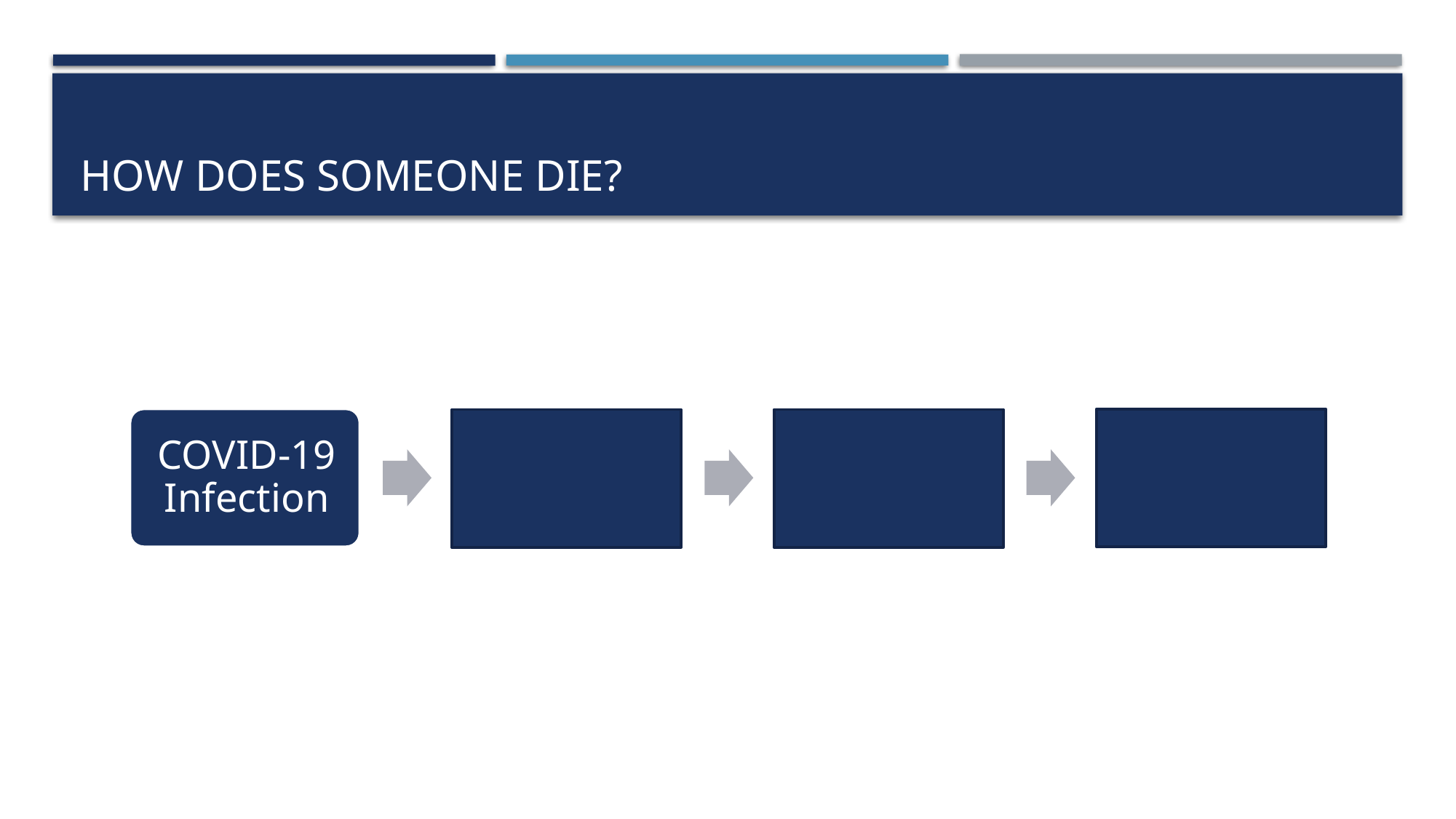

# How does someone die?

## Slide 16
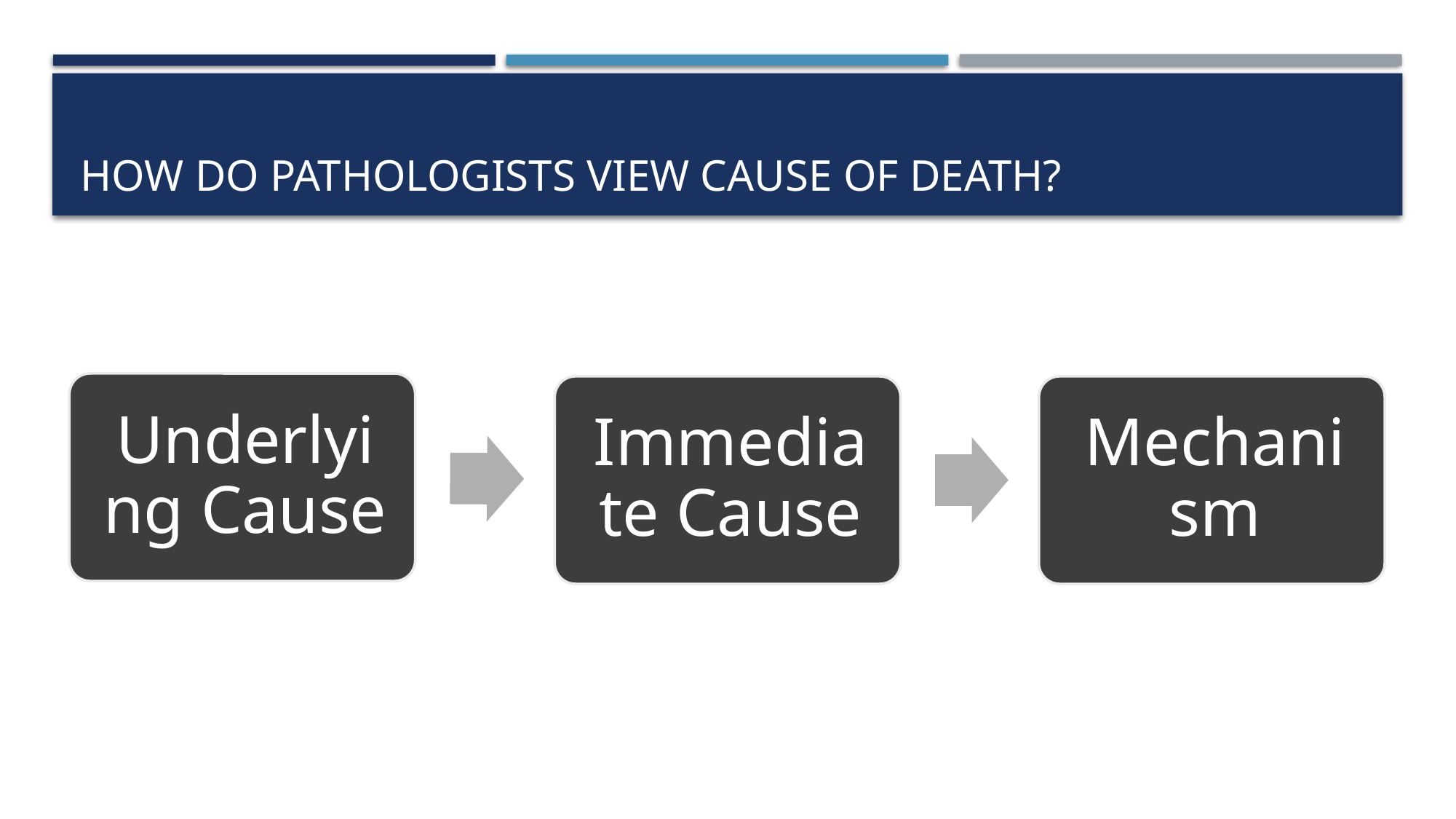

# How do Pathologists view cause of death?

## Slide 17
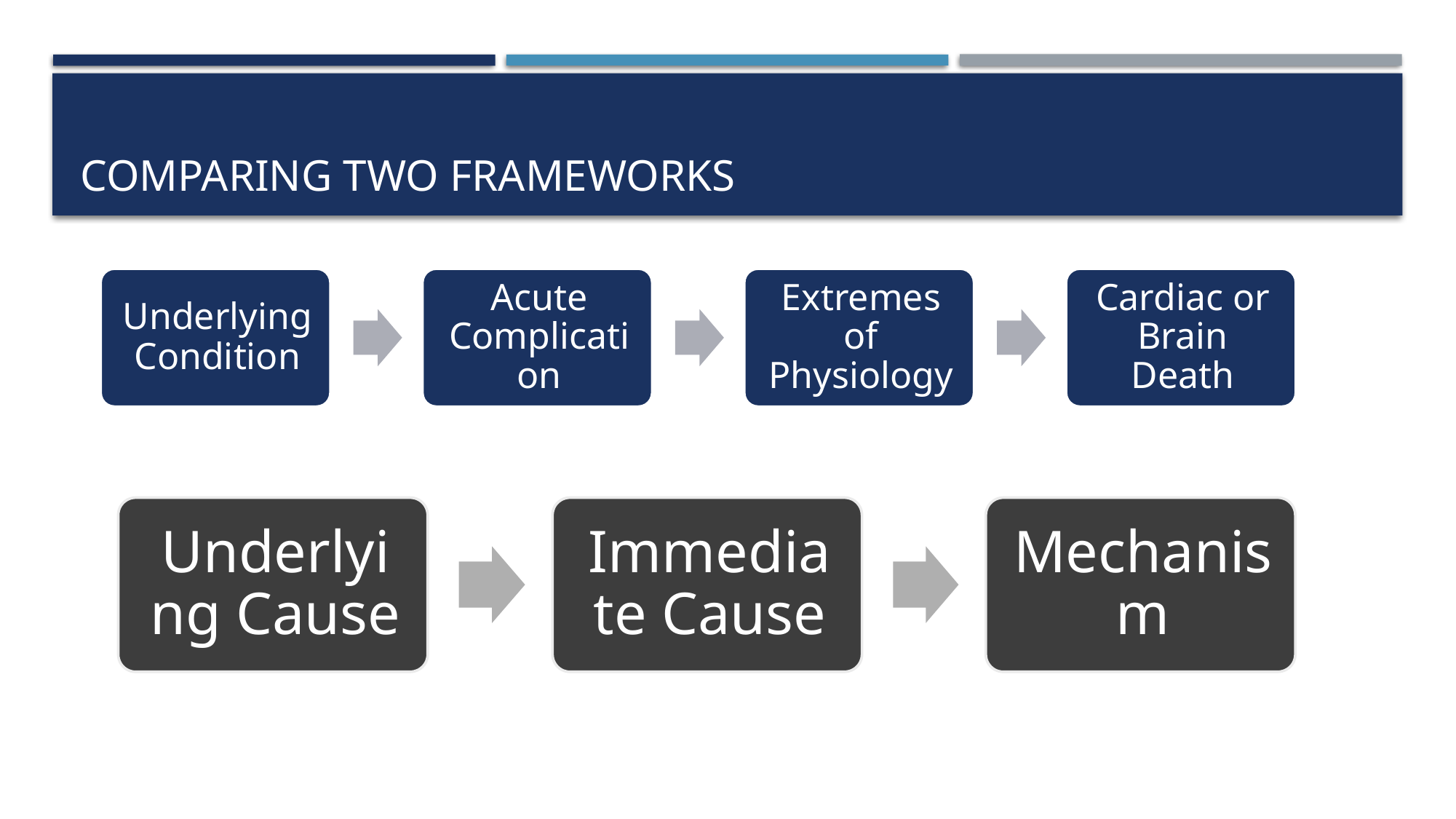

# Comparing two frameworks

## Slide 18
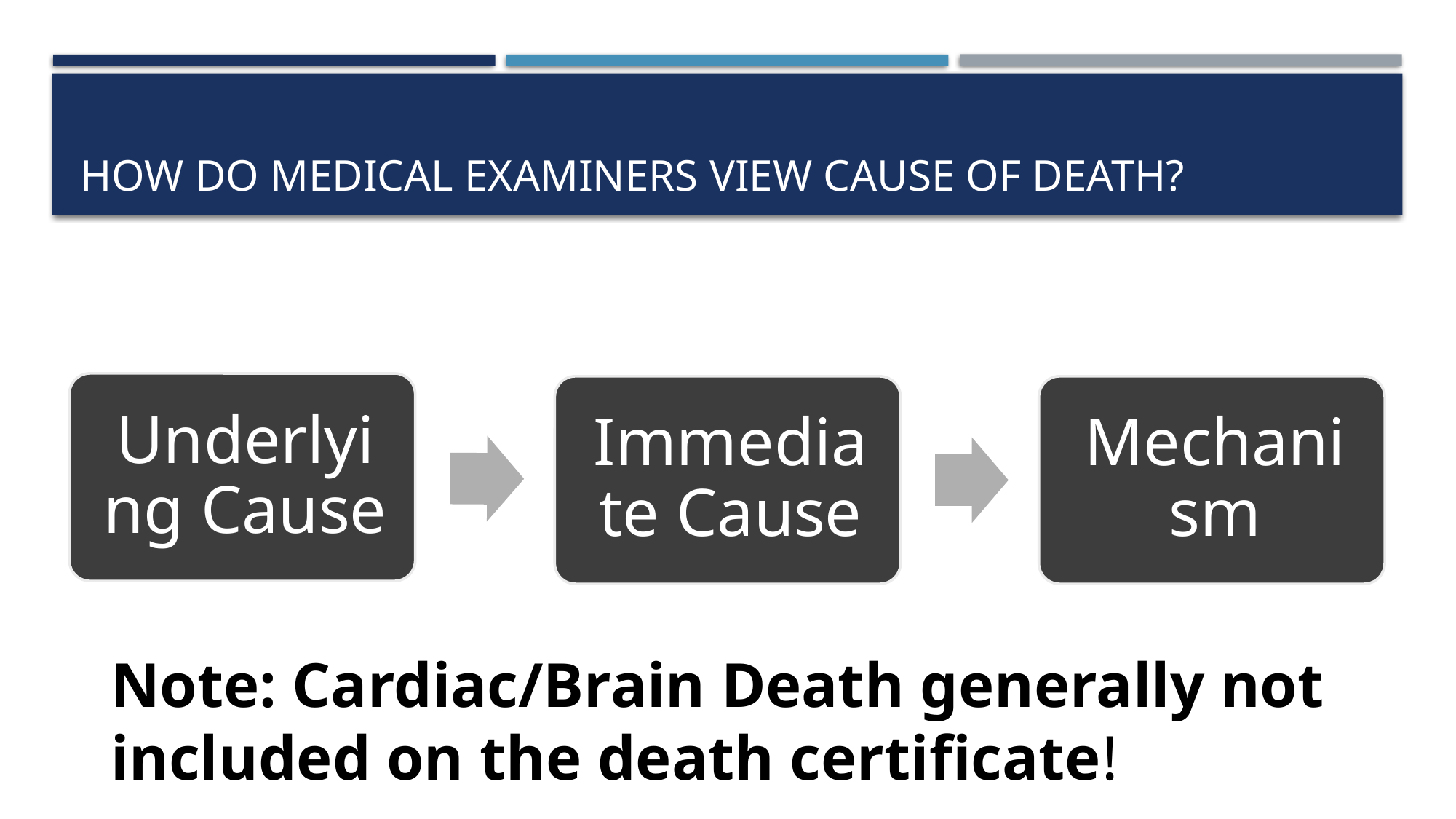

# How do medical examiners view cause of death?
Note: Cardiac/Brain Death generally not included on the death certificate!

## Slide 19
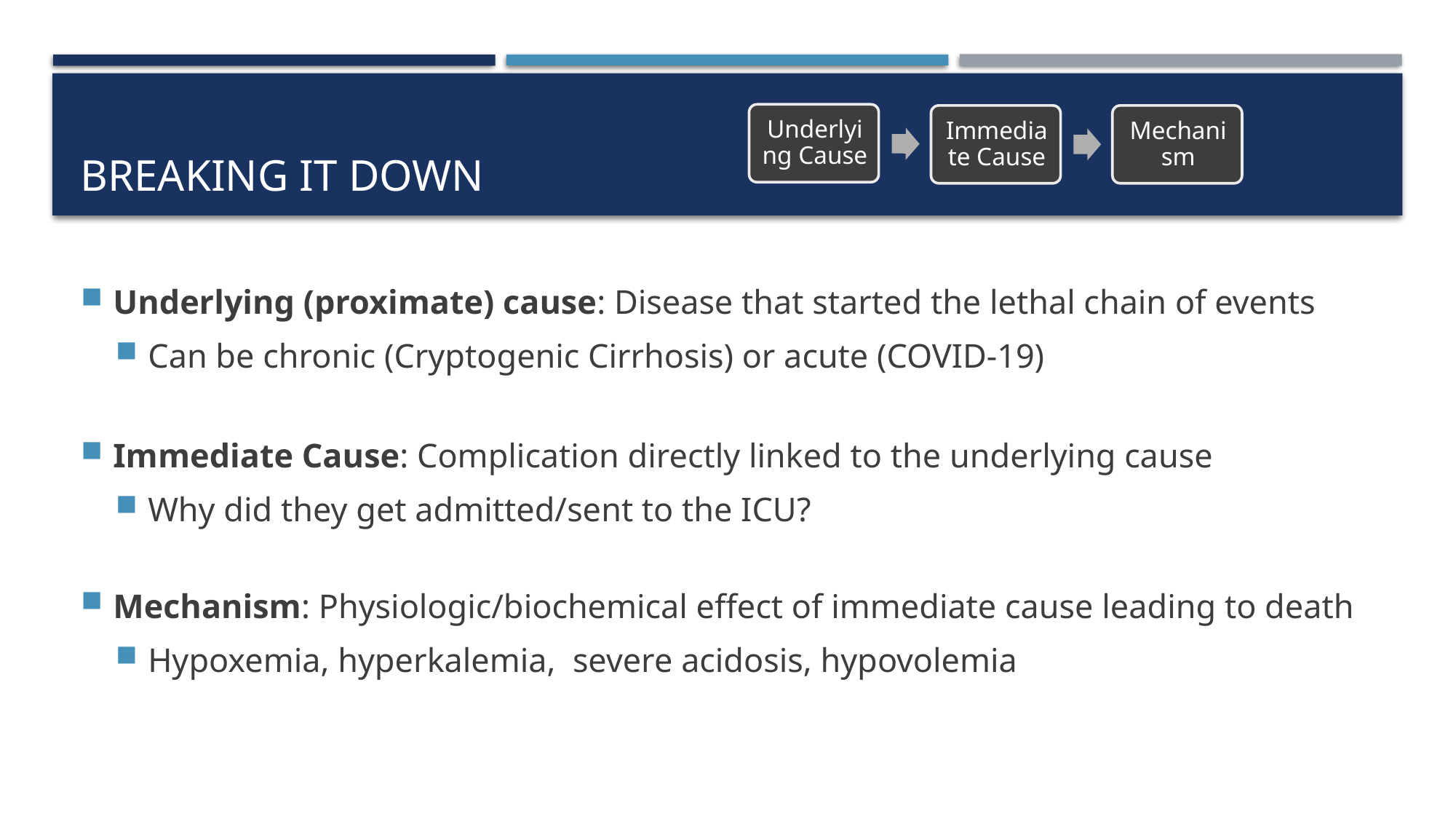

# Breaking it down
Underlying (proximate) cause: Disease that started the lethal chain of events
Can be chronic (Cryptogenic Cirrhosis) or acute (COVID-19)
Immediate Cause: Complication directly linked to the underlying cause
Why did they get admitted/sent to the ICU?
Mechanism: Physiologic/biochemical effect of immediate cause leading to death
Hypoxemia, hyperkalemia,  severe acidosis, hypovolemia

## Slide 20
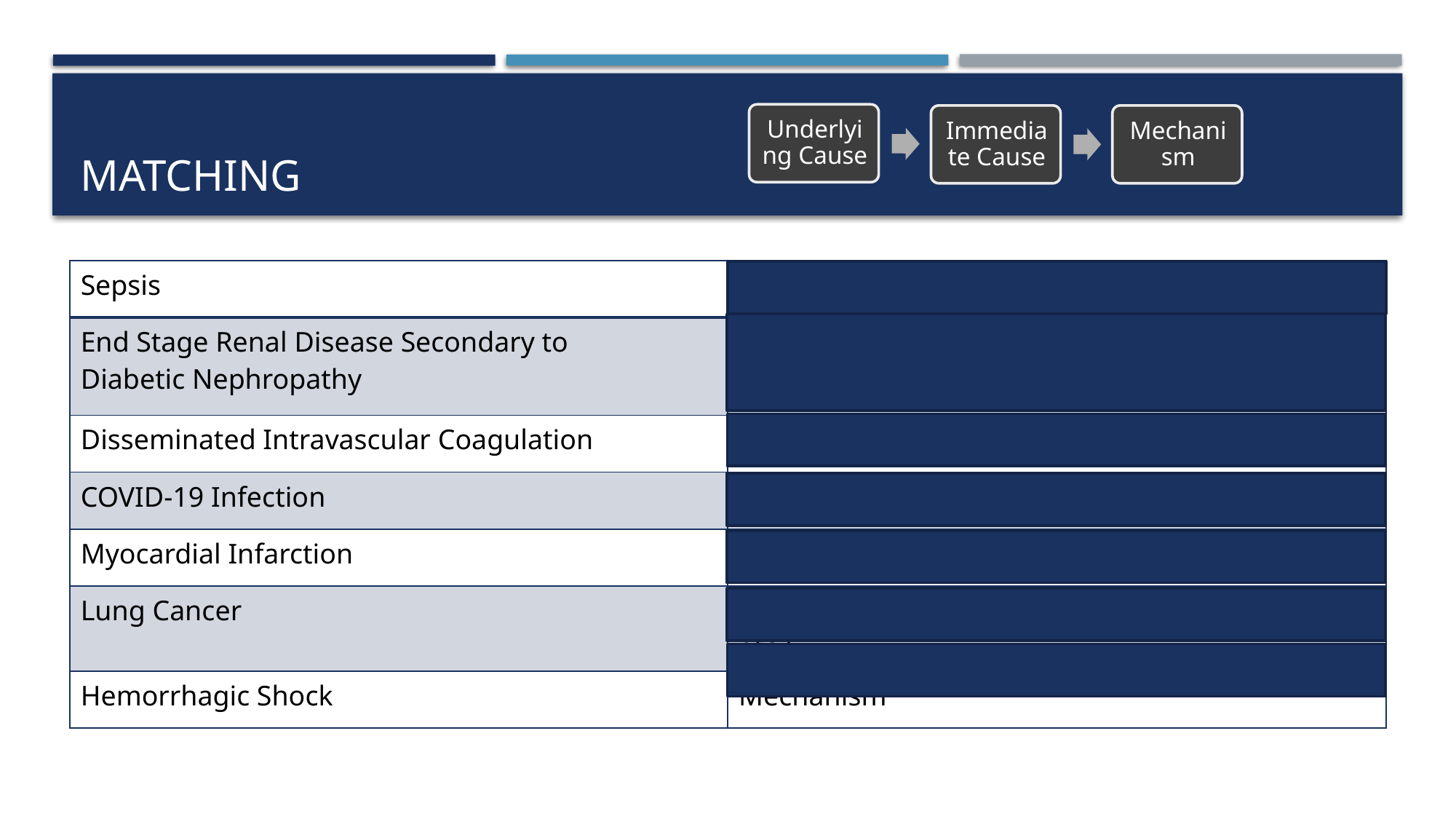

# Matching
| Sepsis | Immediate Cause (What is the source?) |
| --- | --- |
| End Stage Renal Disease Secondary to Diabetic Nephropathy | Underlying Cause |
| Disseminated Intravascular Coagulation | Mechanism |
| COVID-19 Infection | Underlying Cause |
| Myocardial Infarction | Immediate Cause (Atherosclerosis) |
| Lung Cancer | Underlying or Immediate (Tobacco Use Disorder, etc.) |
| Hemorrhagic Shock | Mechanism |

## Slide 21
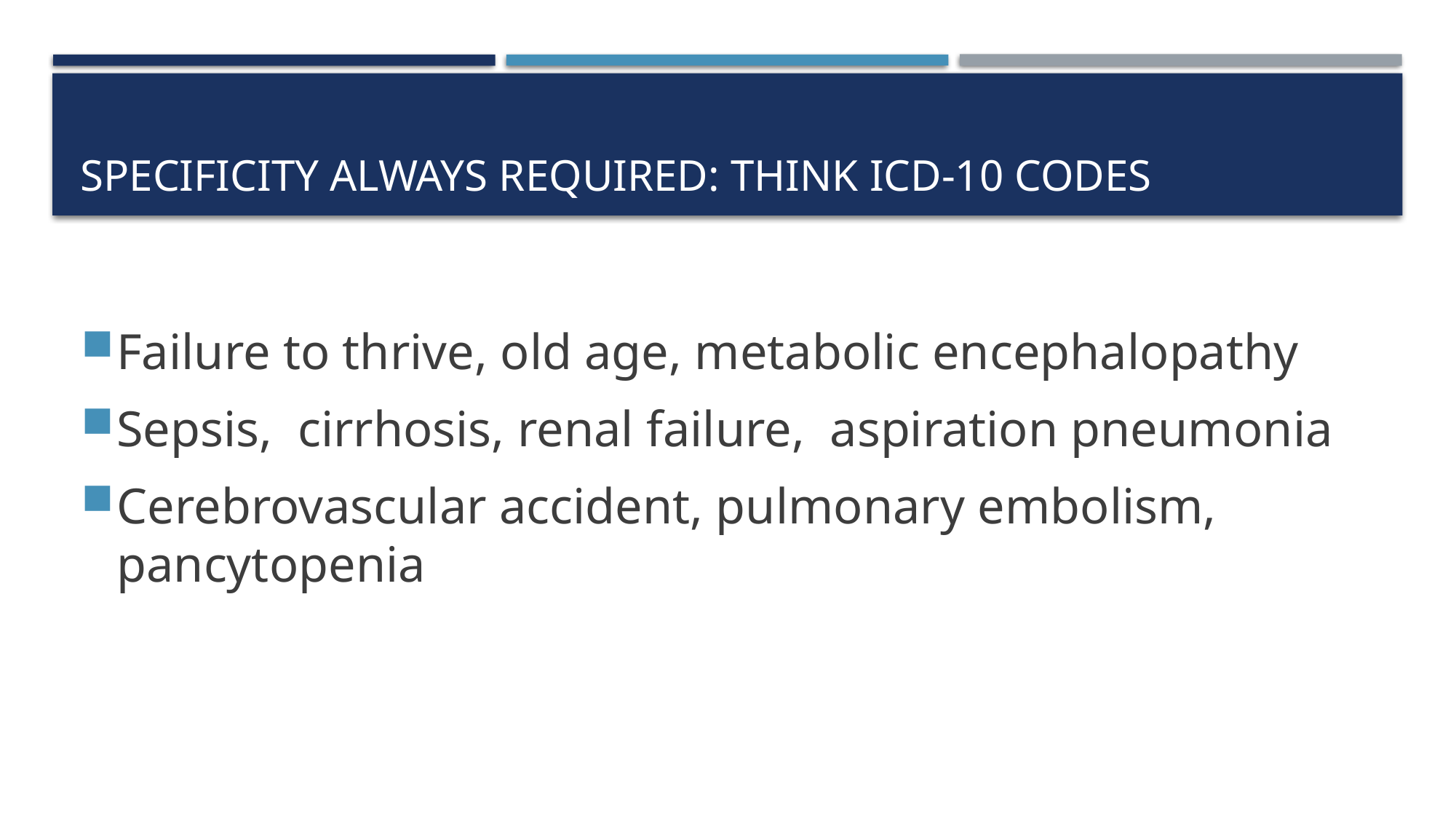

# Specificity Always required: Think ICD-10 Codes
Failure to thrive, old age, metabolic encephalopathy
Sepsis, cirrhosis, renal failure, aspiration pneumonia
Cerebrovascular accident, pulmonary embolism, pancytopenia

## Slide 22
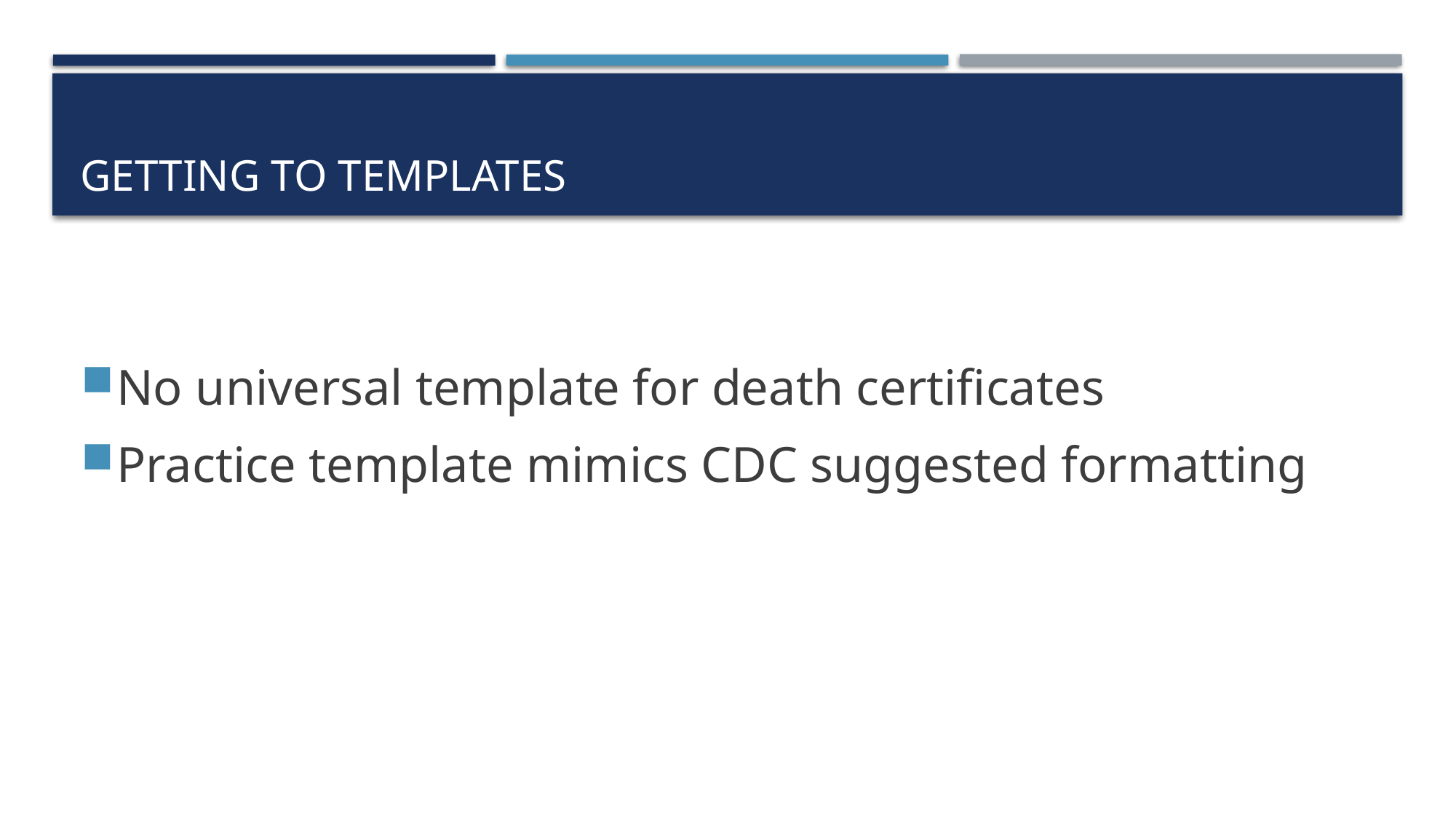

# Getting to templates
No universal template for death certificates
Practice template mimics CDC suggested formatting

## Slide 23
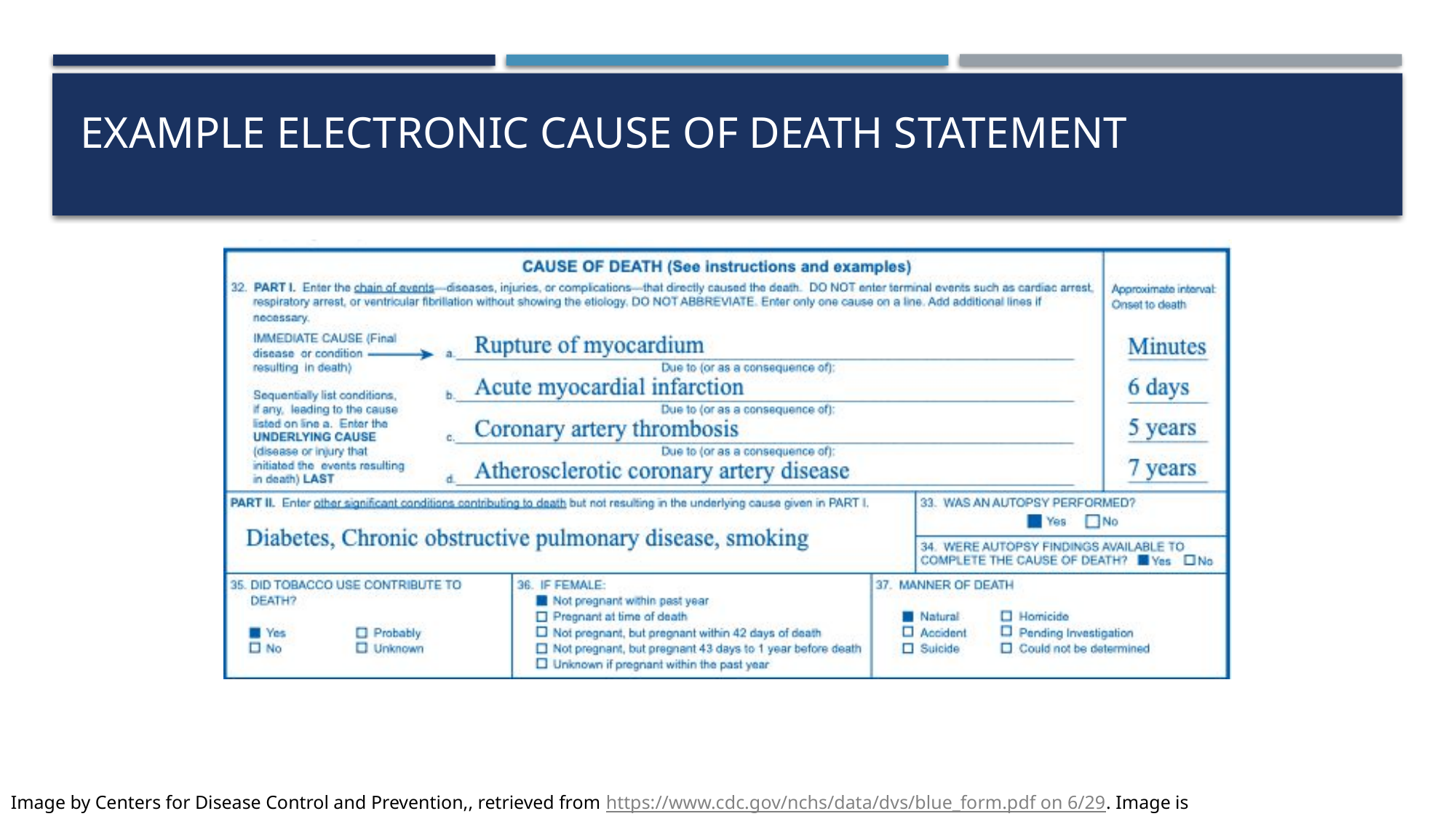

# Example electronic Cause of death Statement
Image by Centers for Disease Control and Prevention,, retrieved from https://www.cdc.gov/nchs/data/dvs/blue_form.pdf on 6/29. Image is in the public domain.

## Slide 24
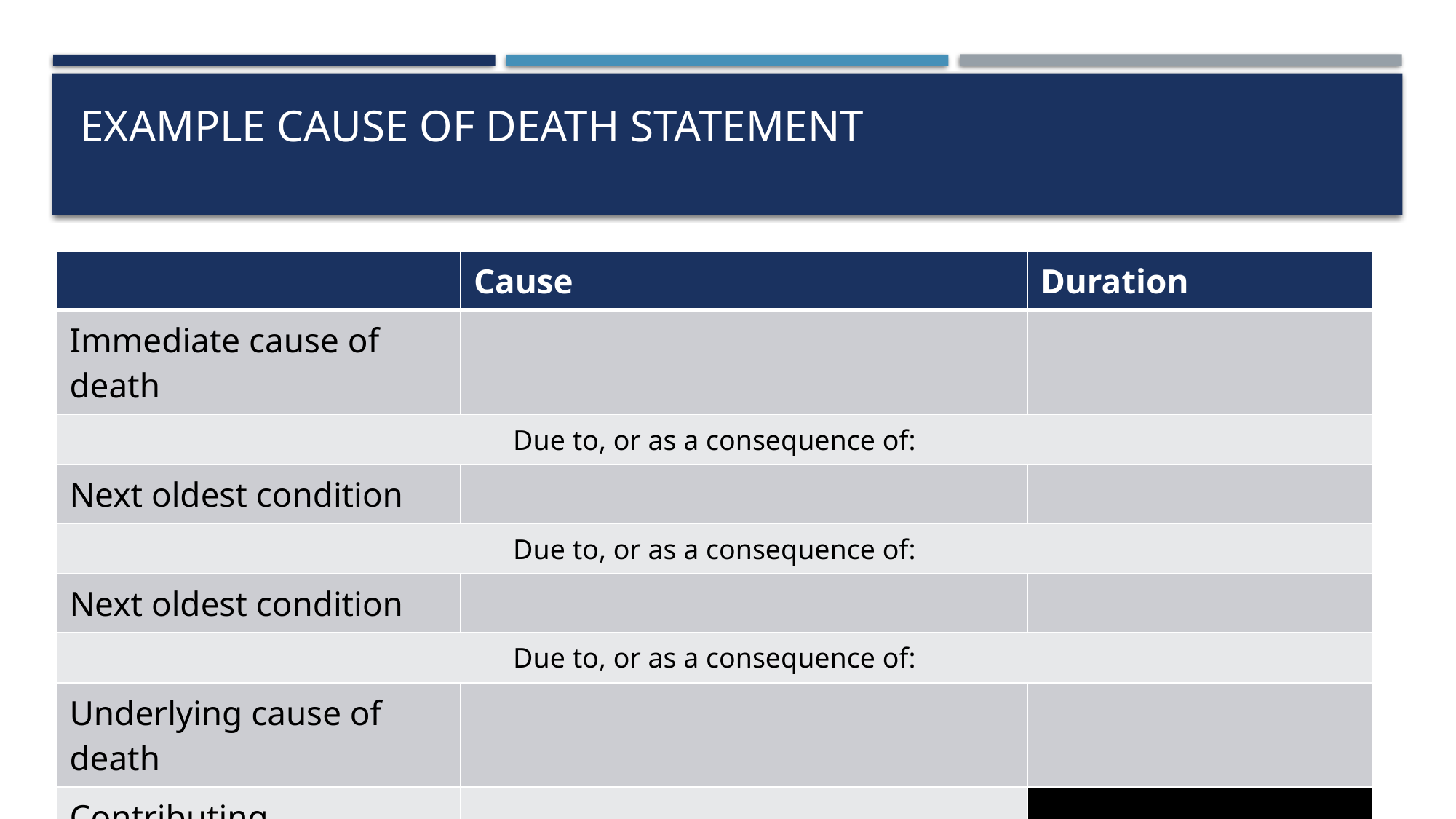

# Example Cause of death statement
| | Cause | Duration |
| --- | --- | --- |
| Immediate cause of death | | |
| Due to, or as a consequence of: | | |
| Next oldest condition | | |
| Due to, or as a consequence of: | | |
| Next oldest condition | | |
| Due to, or as a consequence of: | | |
| Underlying cause of death | | |
| Contributing condition(s) | | |

## Slide 25
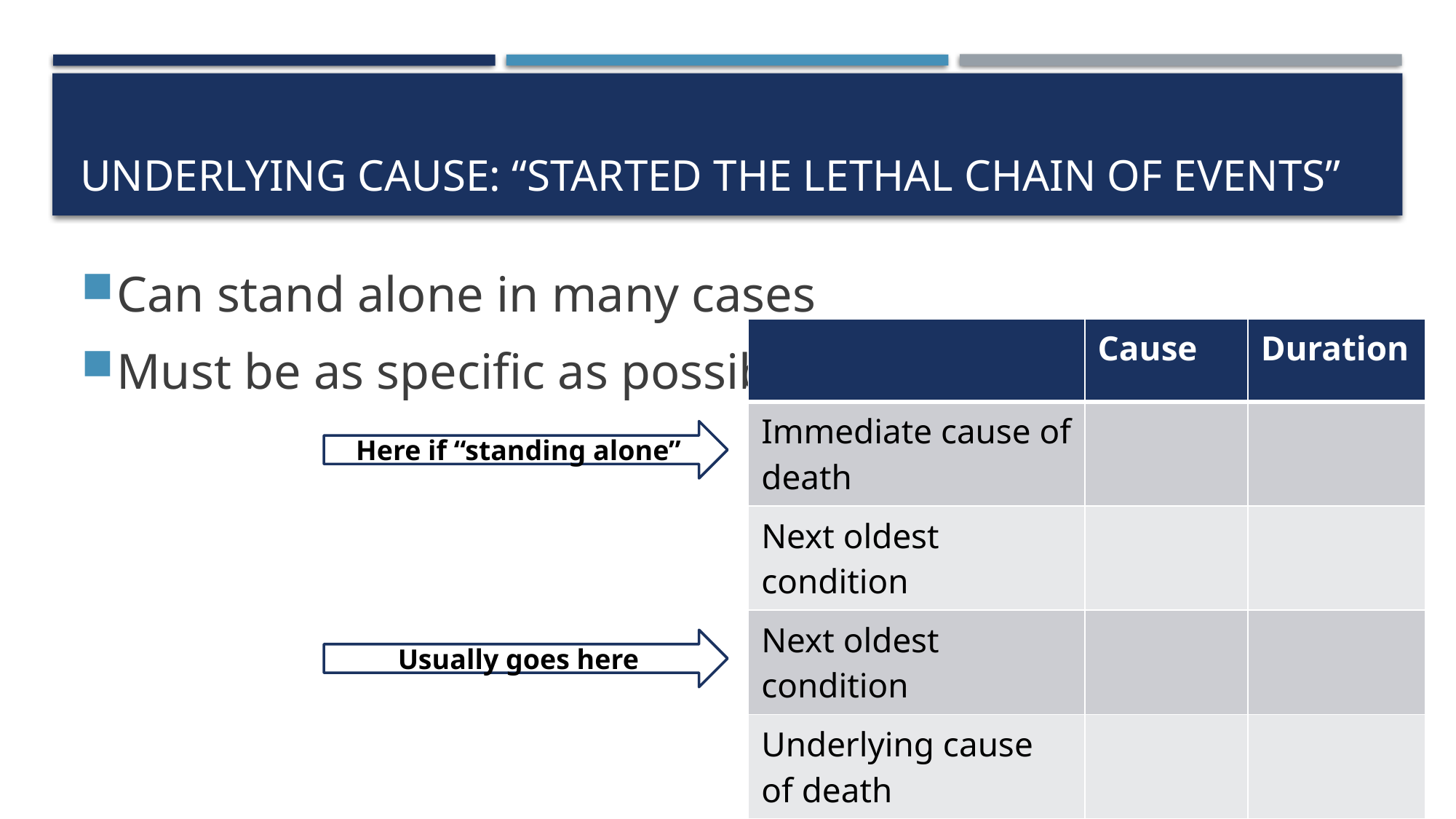

# Underlying Cause: “started the lethal chain of events”
Can stand alone in many cases
Must be as specific as possible
| | Cause | Duration |
| --- | --- | --- |
| Immediate cause of death | | |
| Next oldest condition | | |
| Next oldest condition | | |
| Underlying cause of death | | |
| Contributing condition(s) | | |
Here if “standing alone”
Usually goes here

## Slide 26
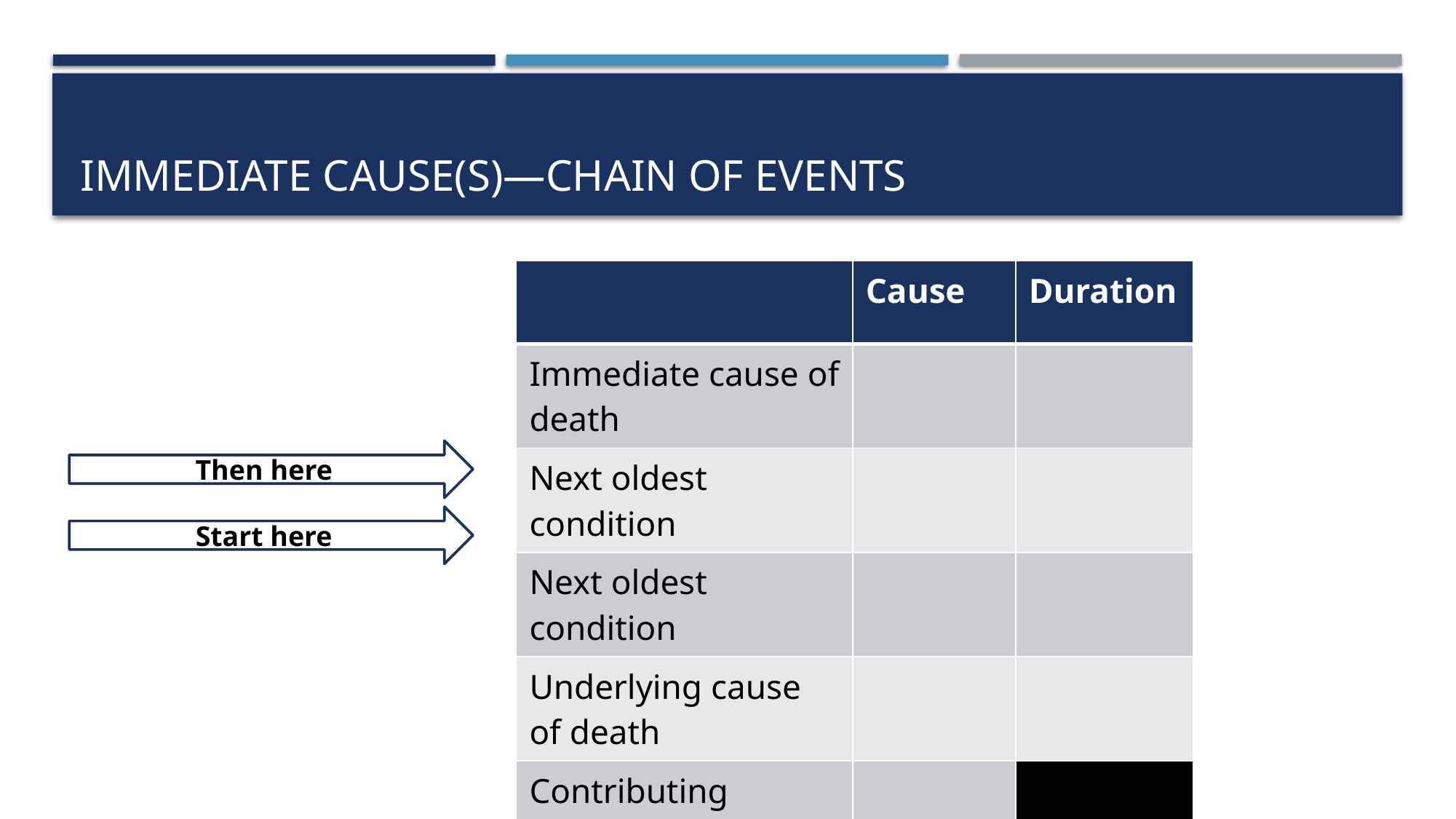

# Immediate Cause(s)—chain of events
| | Cause | Duration |
| --- | --- | --- |
| Immediate cause of death | | |
| Next oldest condition | | |
| Next oldest condition | | |
| Underlying cause of death | | |
| Contributing condition(s) | | |
Then here
Start here

## Slide 27
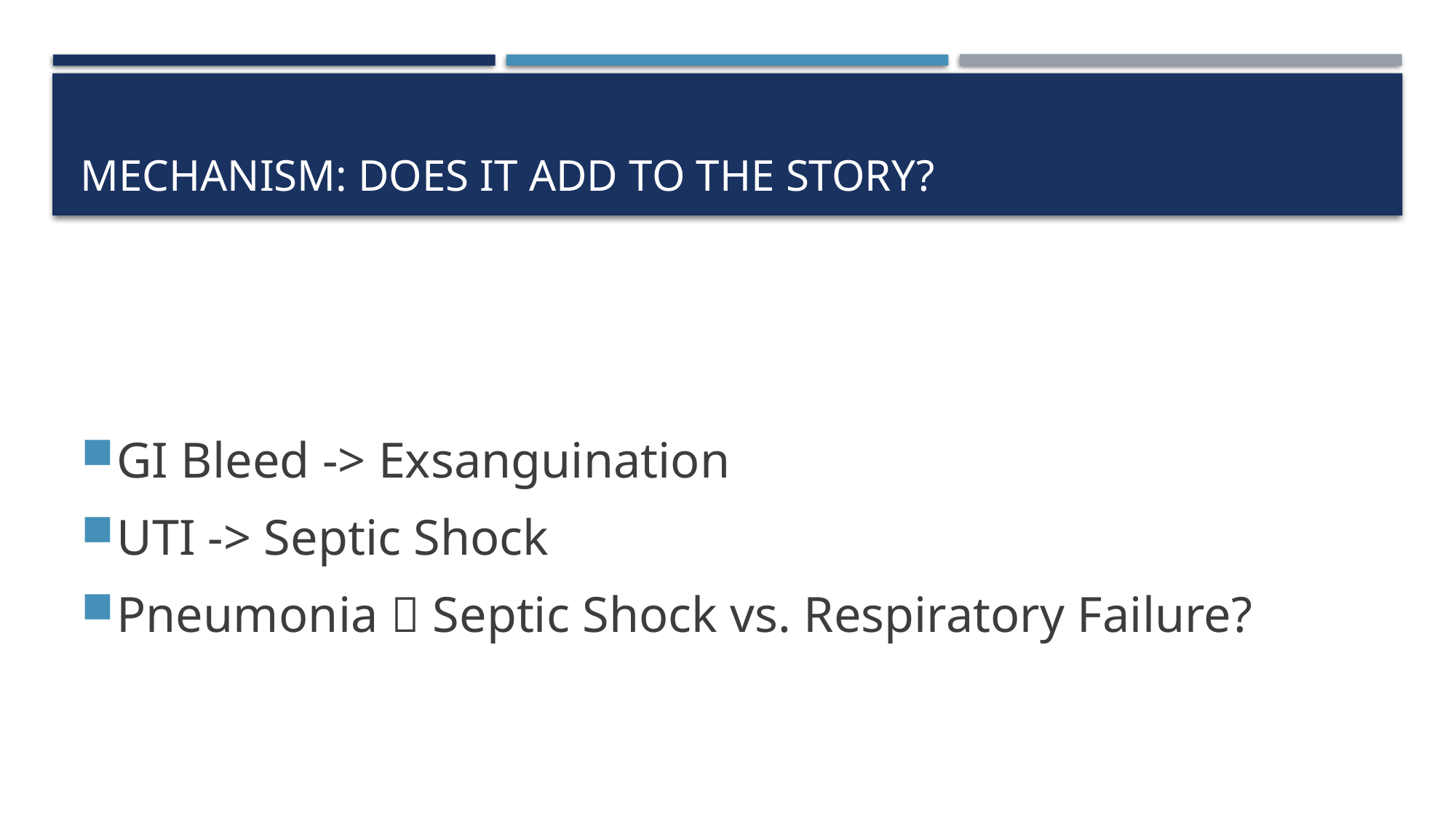

# Mechanism: Does it add to the story?
GI Bleed -> Exsanguination
UTI -> Septic Shock
Pneumonia  Septic Shock vs. Respiratory Failure?

## Slide 28
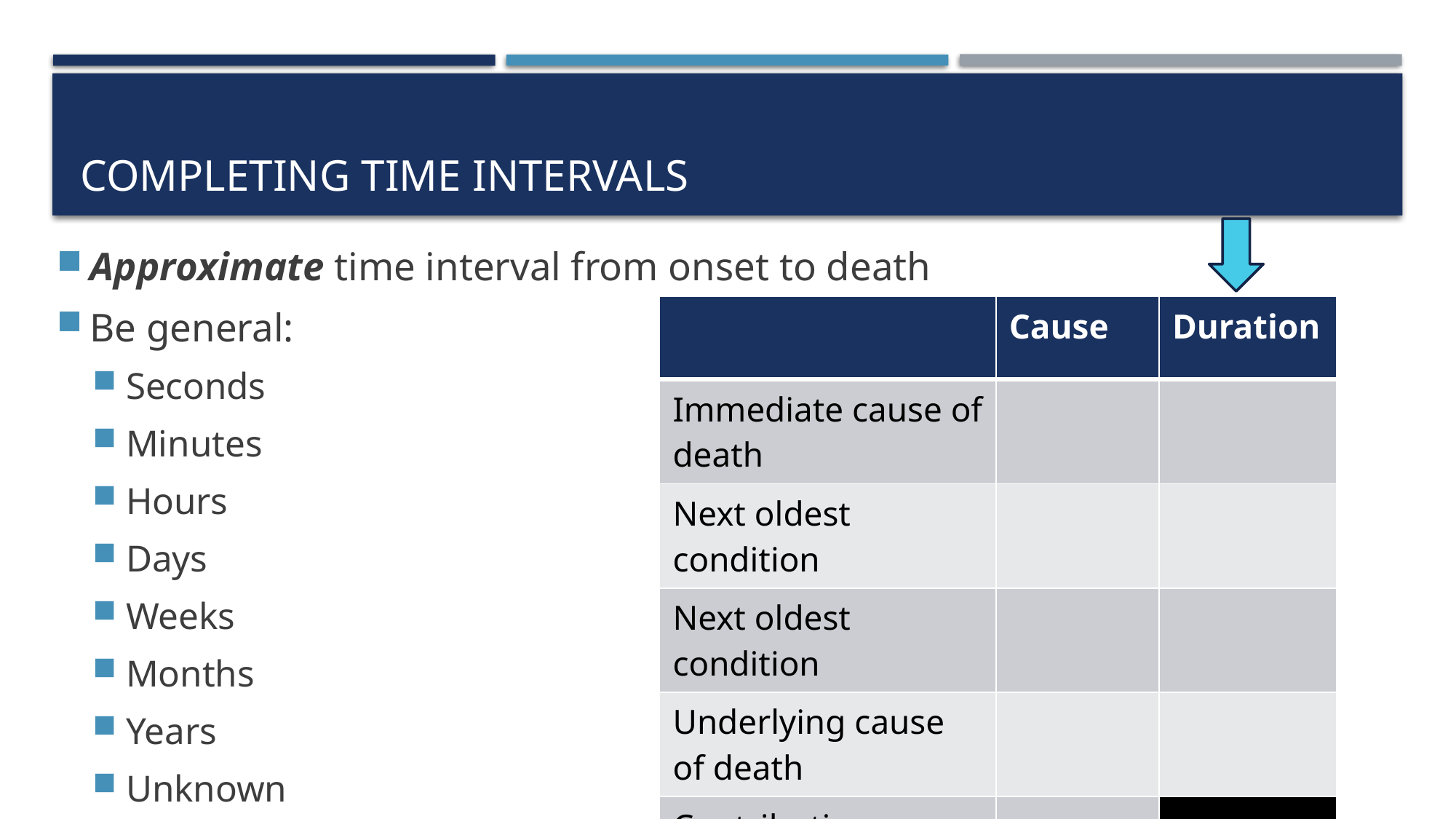

# Completing time intervals
Approximate time interval from onset to death
Be general:
Seconds
Minutes
Hours
Days
Weeks
Months
Years
Unknown
| | Cause | Duration |
| --- | --- | --- |
| Immediate cause of death | | |
| Next oldest condition | | |
| Next oldest condition | | |
| Underlying cause of death | | |
| Contributing condition(s) | | |

## Slide 29
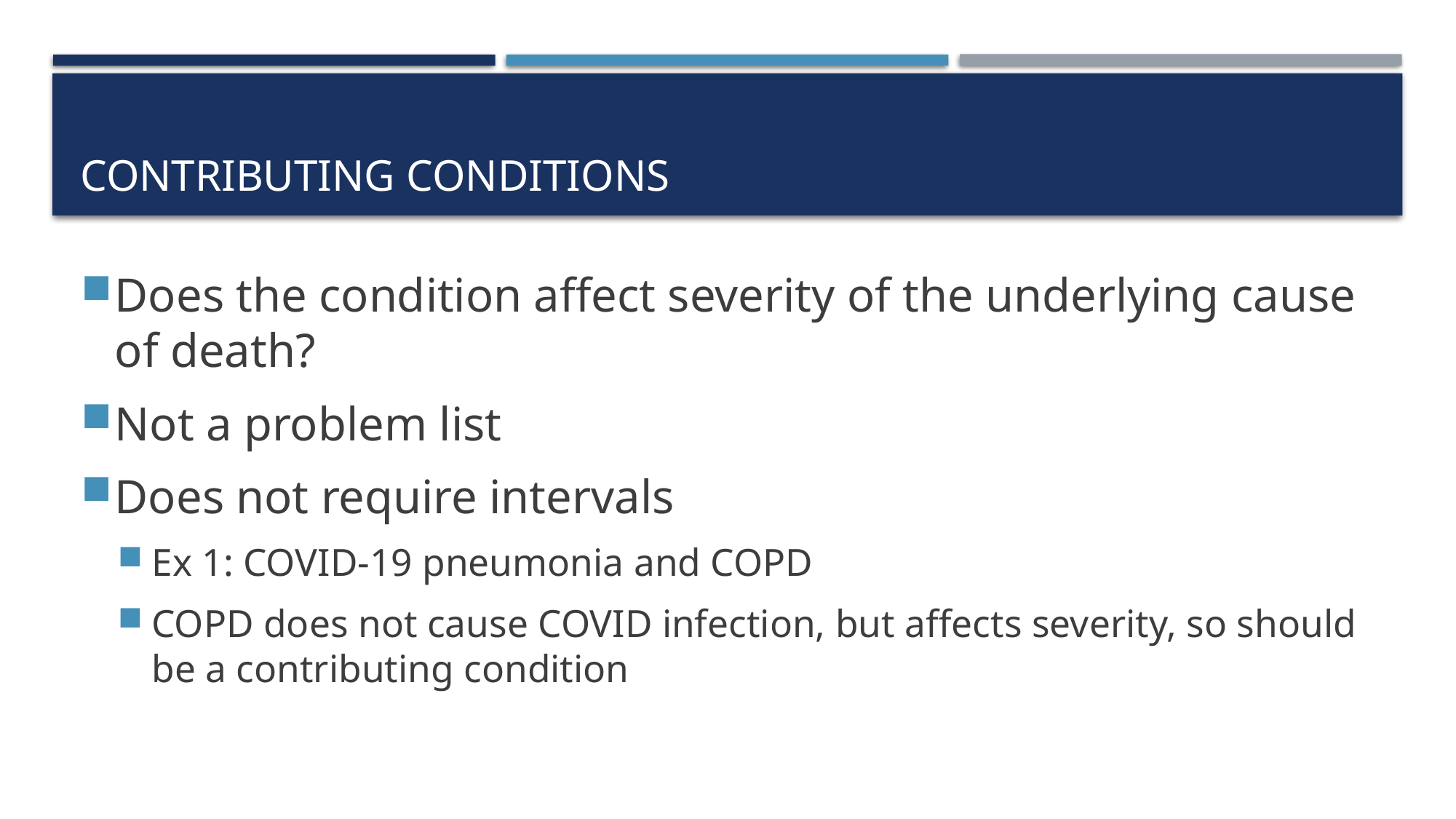

# Contributing Conditions
Does the condition affect severity of the underlying cause of death?
Not a problem list
Does not require intervals
Ex 1: COVID-19 pneumonia and COPD
COPD does not cause COVID infection, but affects severity, so should be a contributing condition

## Slide 30
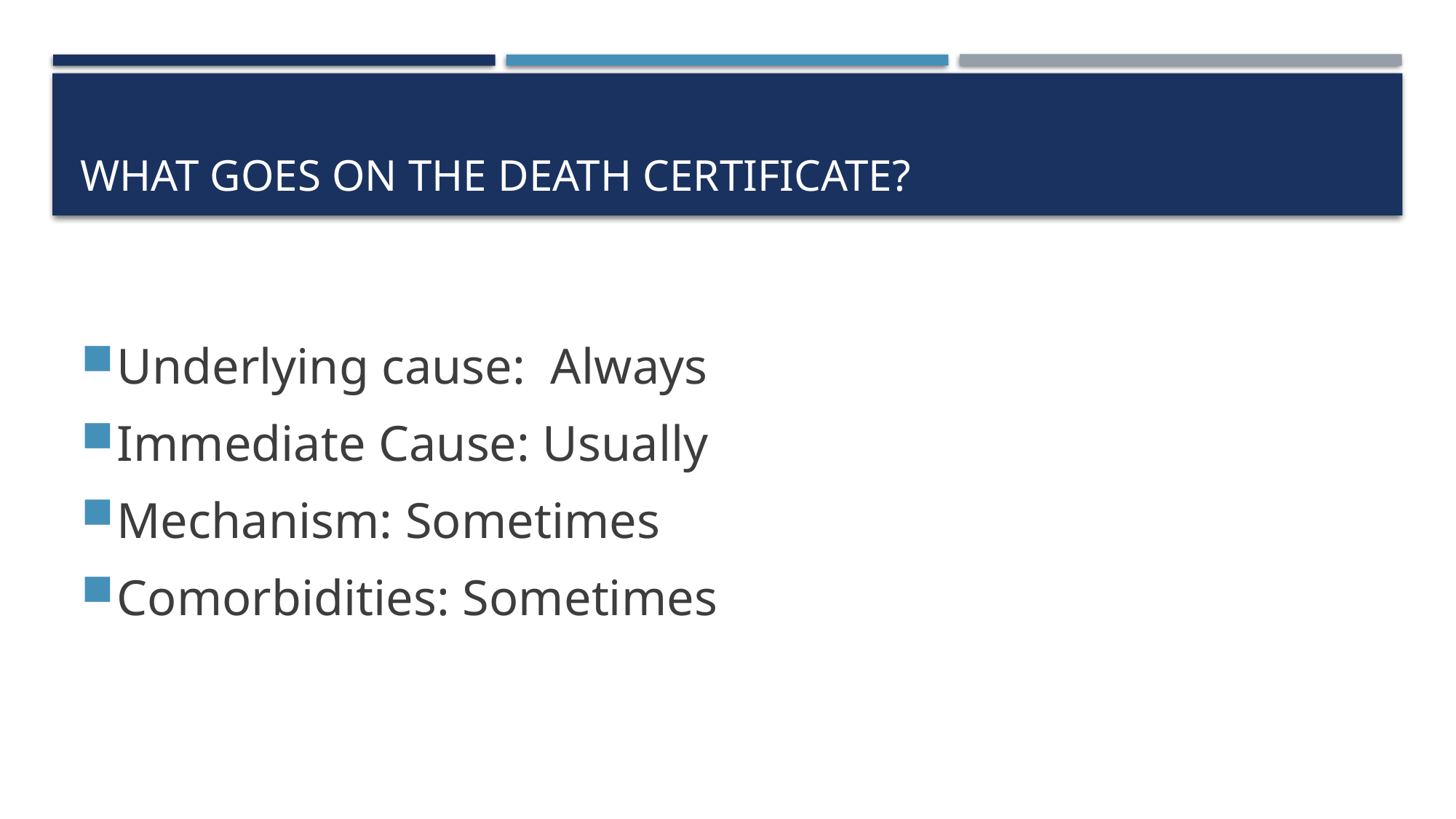

# What goes on the death certificate?
Underlying cause: Always
Immediate Cause: Usually
Mechanism: Sometimes
Comorbidities: Sometimes

## Slide 31
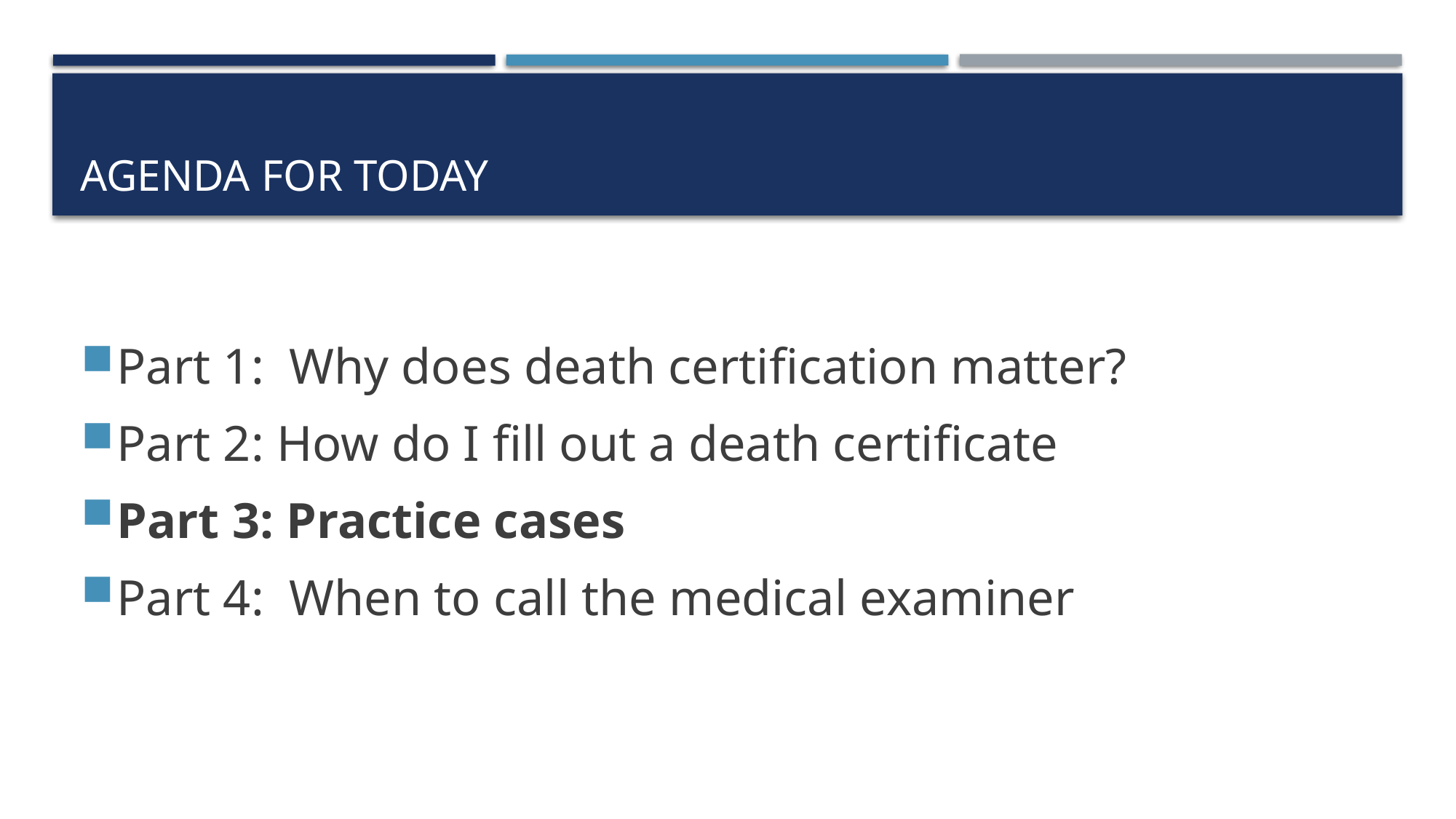

# Agenda for today
Part 1: Why does death certification matter?
Part 2: How do I fill out a death certificate
Part 3: Practice cases
Part 4: When to call the medical examiner

## Slide 32
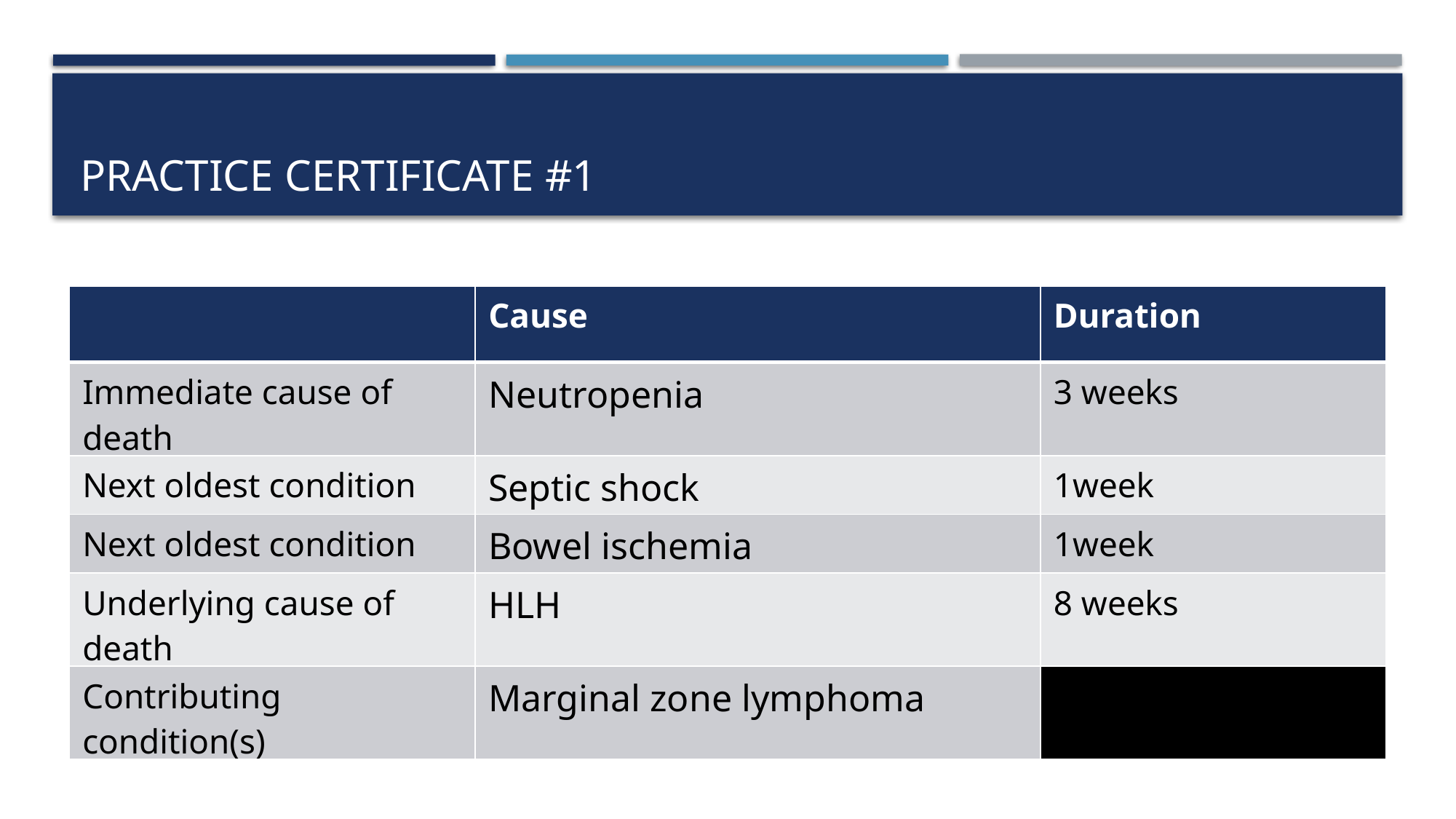

# Practice Certificate #1
| | Cause | Duration |
| --- | --- | --- |
| Immediate cause of death | Neutropenia | 3 weeks |
| Next oldest condition | Septic shock | 1week |
| Next oldest condition | Bowel ischemia | 1week |
| Underlying cause of death | HLH | 8 weeks |
| Contributing condition(s) | Marginal zone lymphoma | |
months

## Slide 33
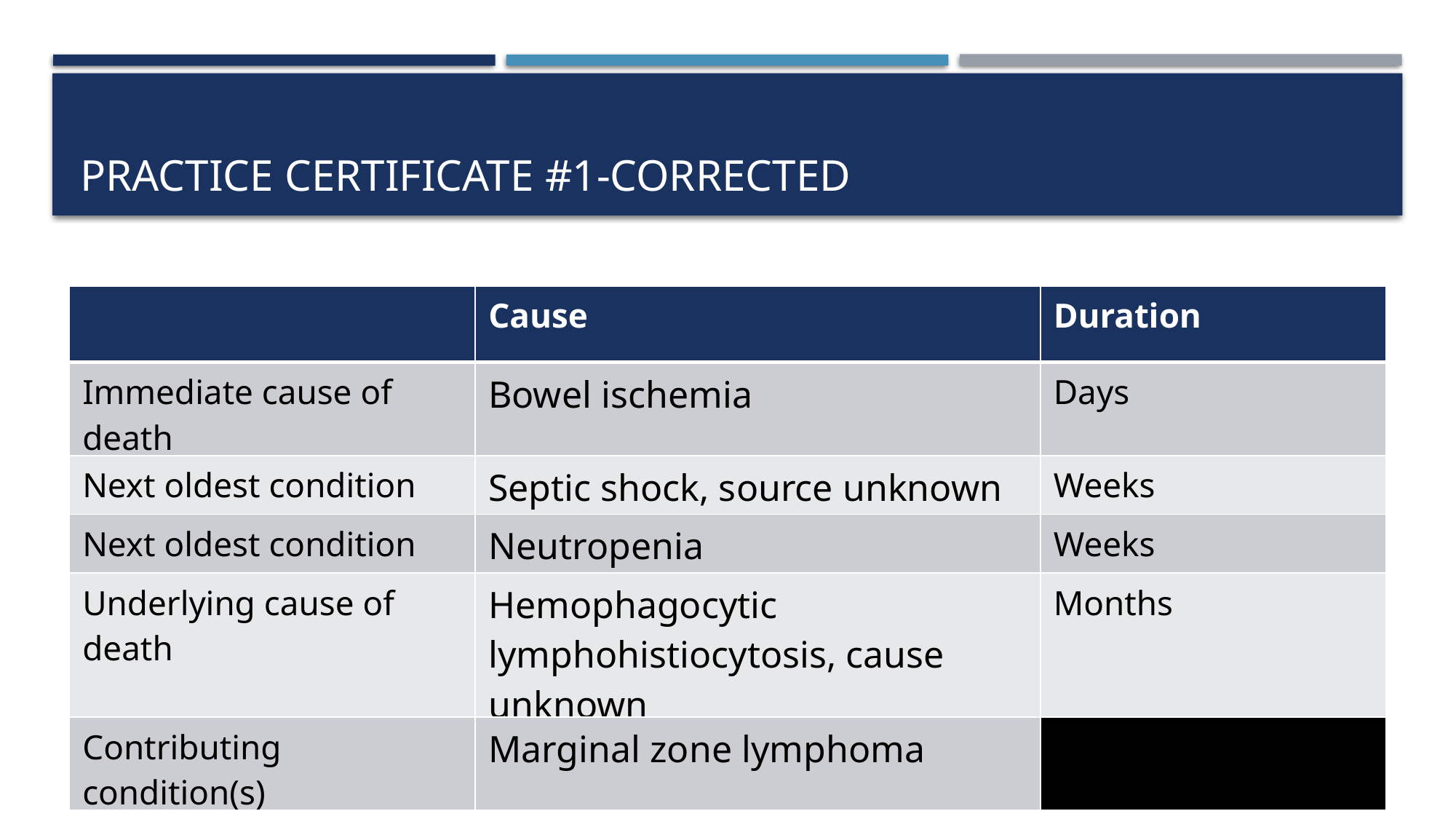

# Practice Certificate #1-Corrected
| | Cause | Duration |
| --- | --- | --- |
| Immediate cause of death | Bowel ischemia | Days |
| Next oldest condition | Septic shock, source unknown | Weeks |
| Next oldest condition | Neutropenia | Weeks |
| Underlying cause of death | Hemophagocytic lymphohistiocytosis, cause unknown | Months |
| Contributing condition(s) | Marginal zone lymphoma | |
months

## Slide 34
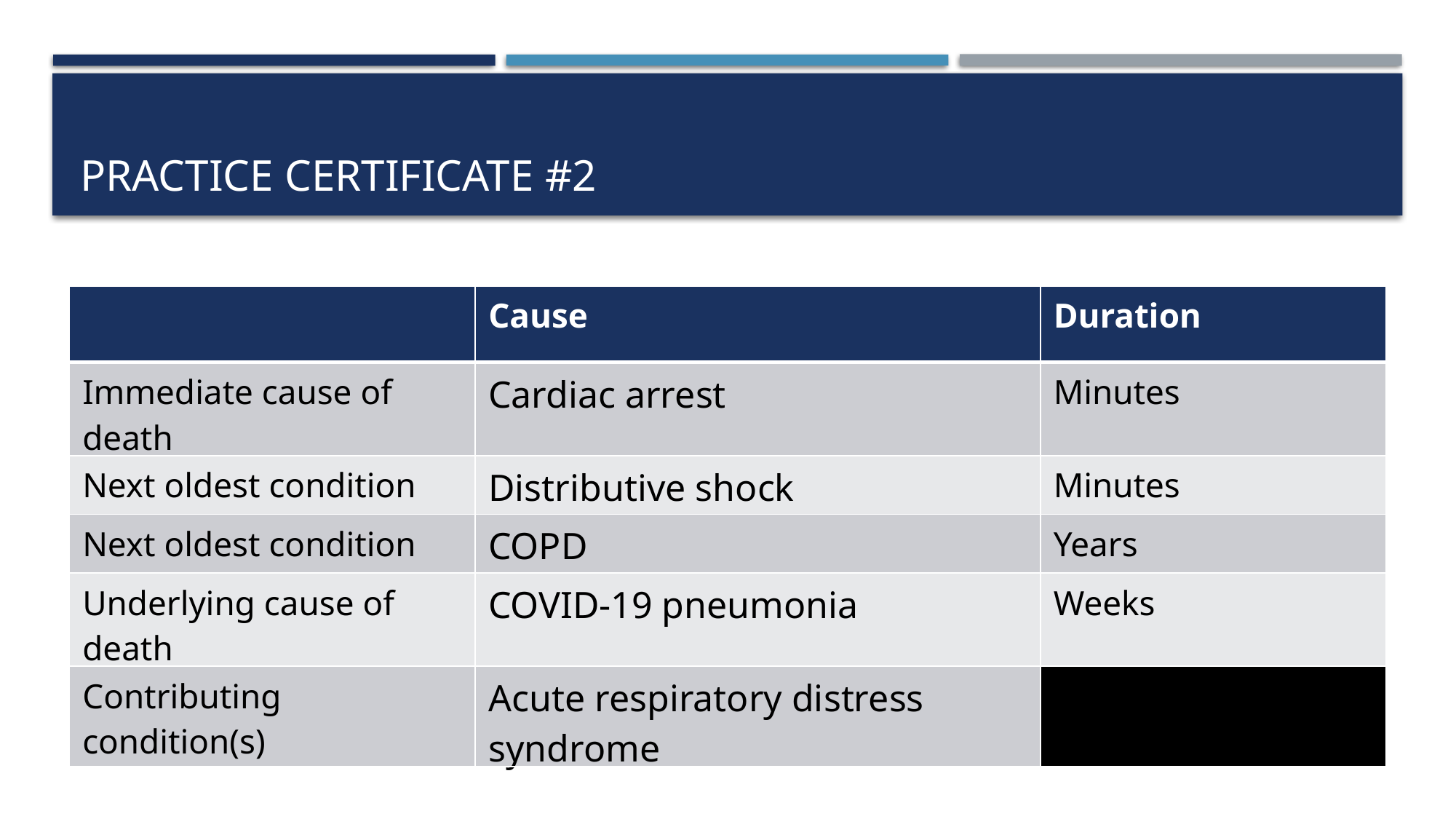

# Practice Certificate #2
| | Cause | Duration |
| --- | --- | --- |
| Immediate cause of death | Cardiac arrest | Minutes |
| Next oldest condition | Distributive shock | Minutes |
| Next oldest condition | COPD | Years |
| Underlying cause of death | COVID-19 pneumonia | Weeks |
| Contributing condition(s) | Acute respiratory distress syndrome | |
months

## Slide 35
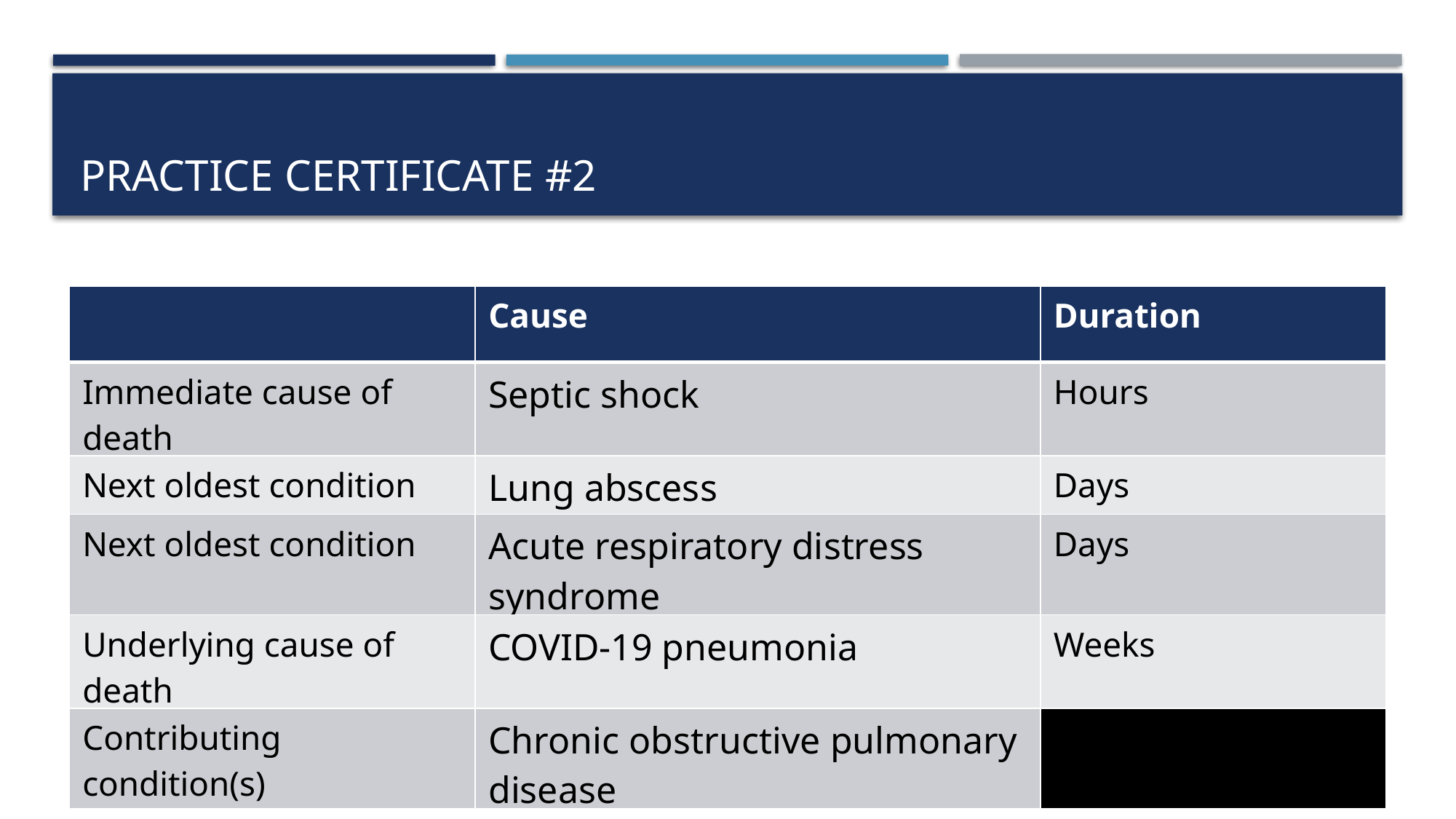

# Practice Certificate #2
| | Cause | Duration |
| --- | --- | --- |
| Immediate cause of death | Septic shock | Hours |
| Next oldest condition | Lung abscess | Days |
| Next oldest condition | Acute respiratory distress syndrome | Days |
| Underlying cause of death | COVID-19 pneumonia | Weeks |
| Contributing condition(s) | Chronic obstructive pulmonary disease | |
months

## Slide 36
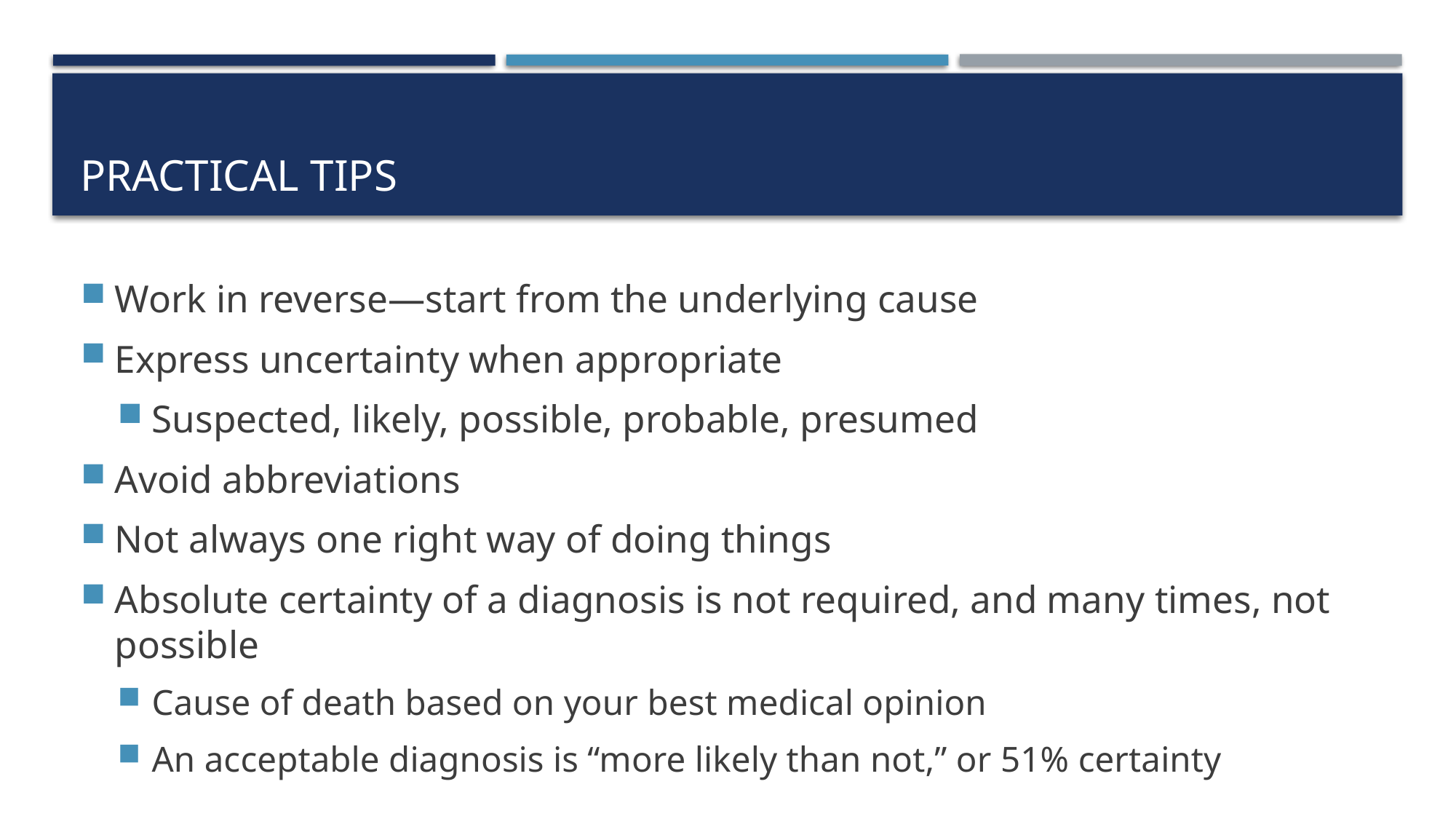

# Practical Tips
Work in reverse—start from the underlying cause
Express uncertainty when appropriate
Suspected, likely, possible, probable, presumed
Avoid abbreviations
Not always one right way of doing things
Absolute certainty of a diagnosis is not required, and many times, not possible
Cause of death based on your best medical opinion
An acceptable diagnosis is “more likely than not,” or 51% certainty

## Slide 37
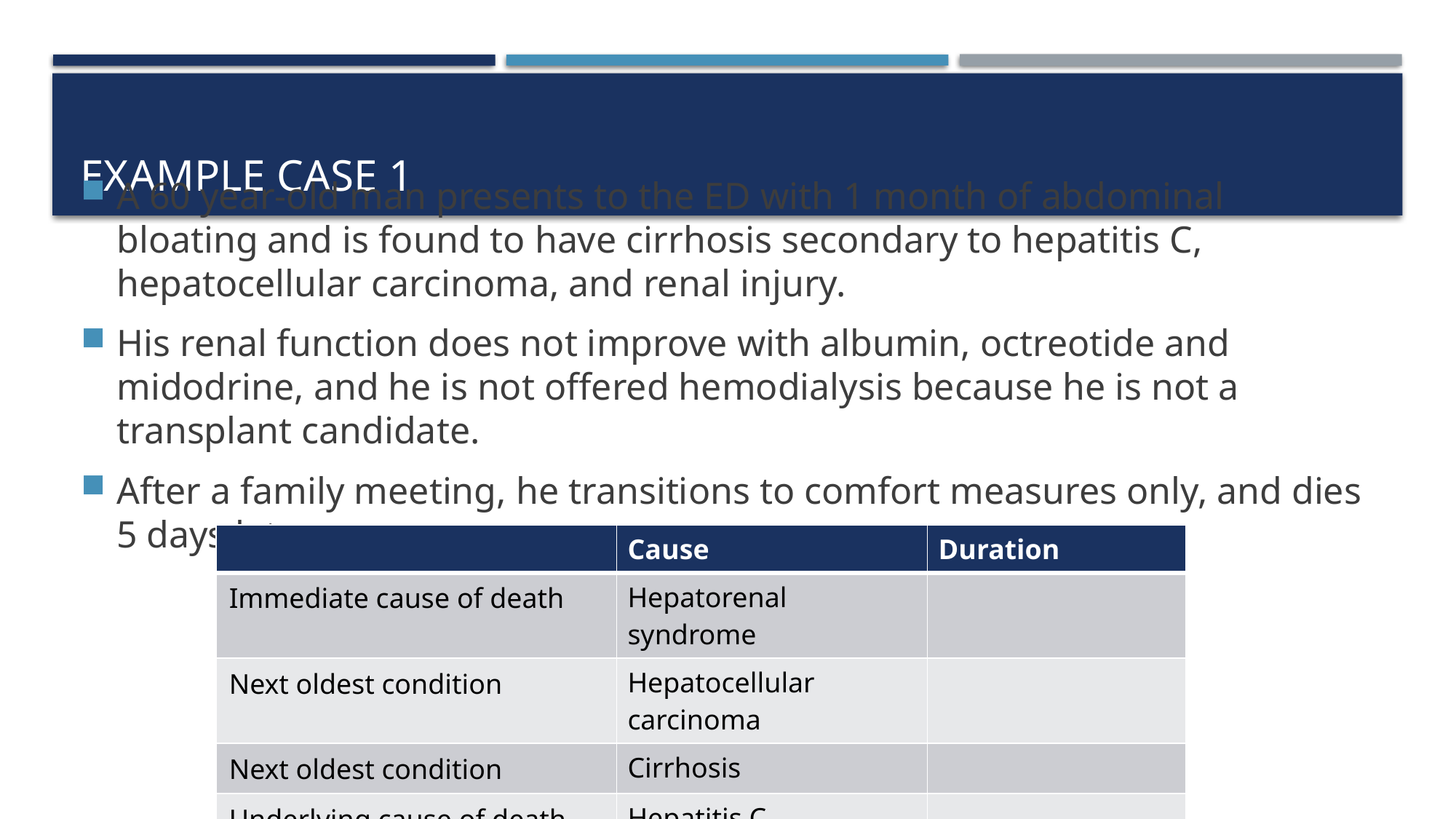

# Example Case 1
A 60 year-old man presents to the ED with 1 month of abdominal bloating and is found to have cirrhosis secondary to hepatitis C, hepatocellular carcinoma, and renal injury.
His renal function does not improve with albumin, octreotide and midodrine, and he is not offered hemodialysis because he is not a transplant candidate.
After a family meeting, he transitions to comfort measures only, and dies 5 days later.
| | Cause | Duration |
| --- | --- | --- |
| Immediate cause of death | Hepatorenal syndrome | |
| Next oldest condition | Hepatocellular carcinoma | |
| Next oldest condition | Cirrhosis | |
| Underlying cause of death | Hepatitis C | |
| Contributing condition(s) | | |

## Slide 38
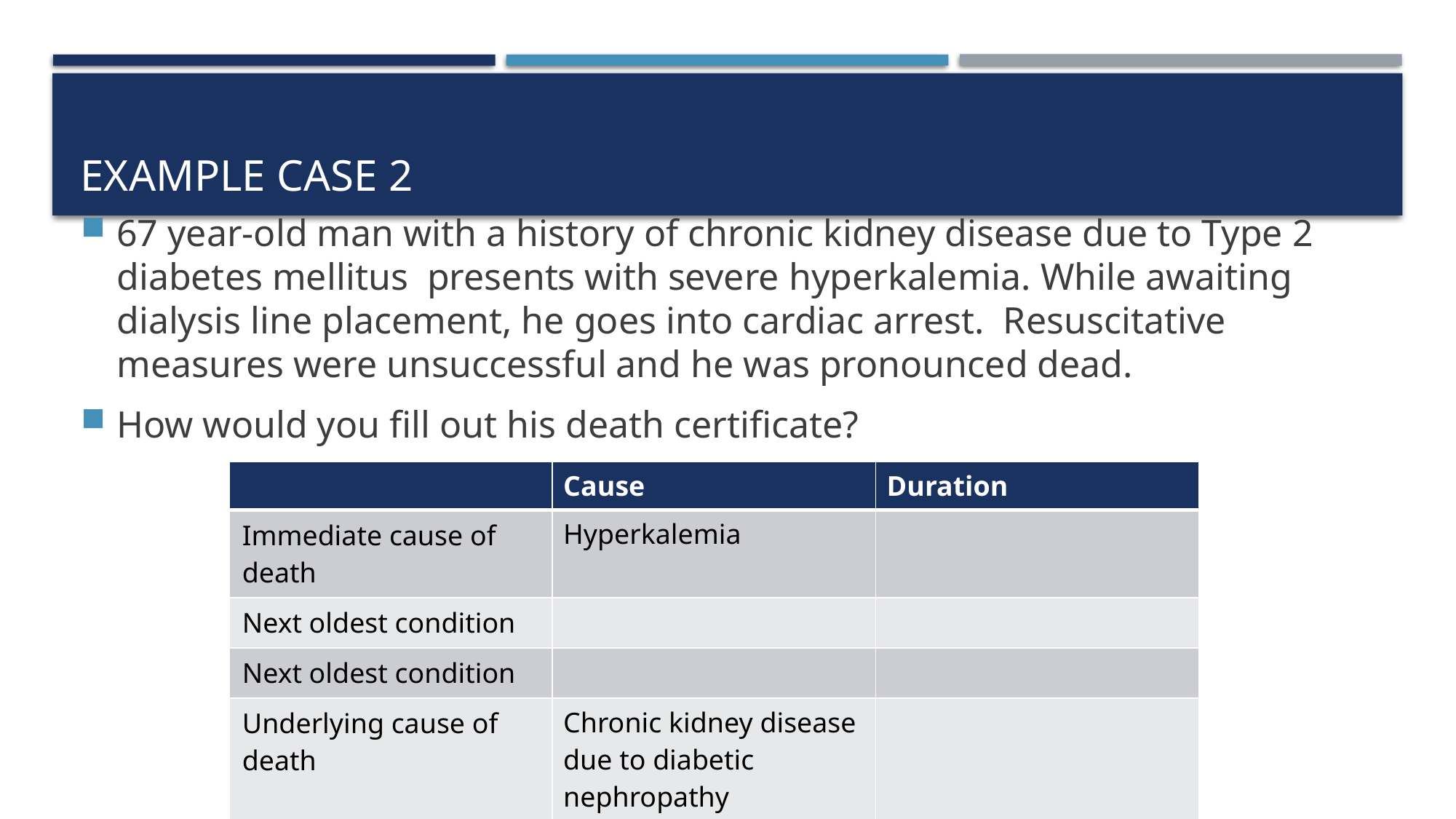

# Example Case 2
67 year-old man with a history of chronic kidney disease due to Type 2 diabetes mellitus presents with severe hyperkalemia. While awaiting dialysis line placement, he goes into cardiac arrest. Resuscitative measures were unsuccessful and he was pronounced dead.
How would you fill out his death certificate?
| | Cause | Duration |
| --- | --- | --- |
| Immediate cause of death | Hyperkalemia | |
| Next oldest condition | | |
| Next oldest condition | | |
| Underlying cause of death | Chronic kidney disease due to diabetic nephropathy | |
| Contributing condition(s) | | |

## Slide 39
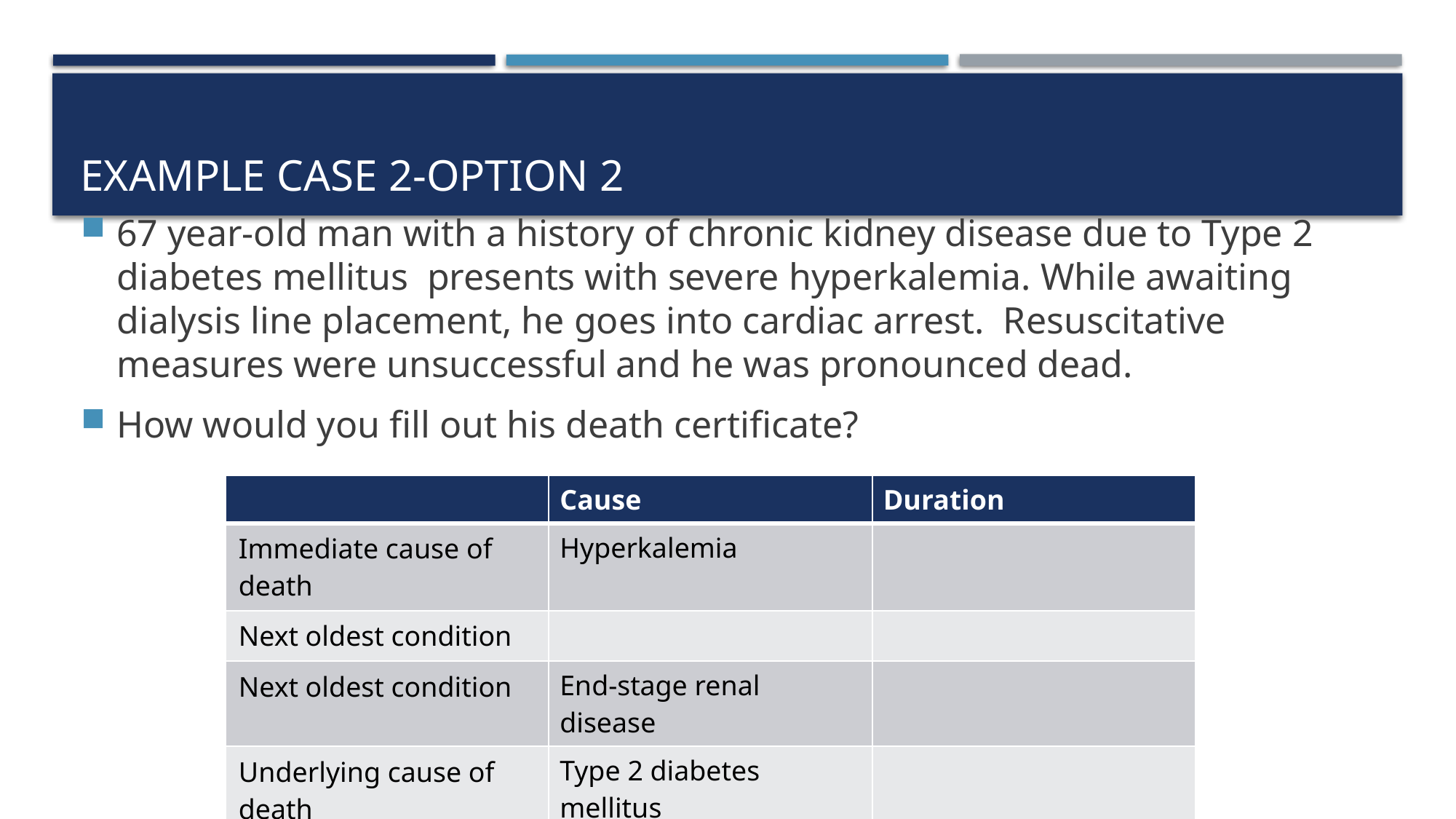

# Example Case 2-Option 2
67 year-old man with a history of chronic kidney disease due to Type 2 diabetes mellitus presents with severe hyperkalemia. While awaiting dialysis line placement, he goes into cardiac arrest. Resuscitative measures were unsuccessful and he was pronounced dead.
How would you fill out his death certificate?
| | Cause | Duration |
| --- | --- | --- |
| Immediate cause of death | Hyperkalemia | |
| Next oldest condition | | |
| Next oldest condition | End-stage renal disease | |
| Underlying cause of death | Type 2 diabetes mellitus | |
| Contributing condition(s) | | |

## Slide 40
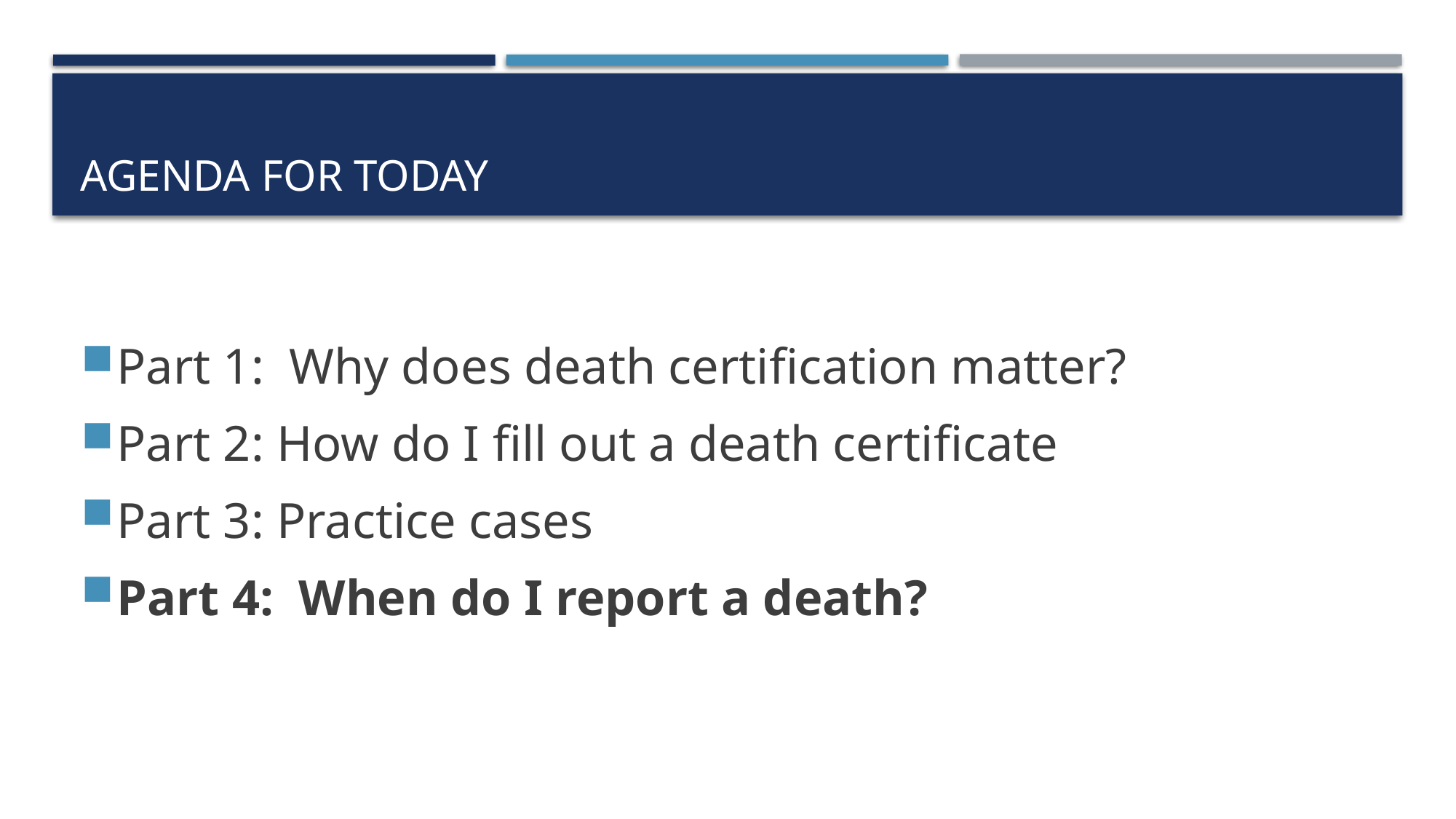

# Agenda for today
Part 1: Why does death certification matter?
Part 2: How do I fill out a death certificate
Part 3: Practice cases
Part 4: When do I report a death?

## Slide 41
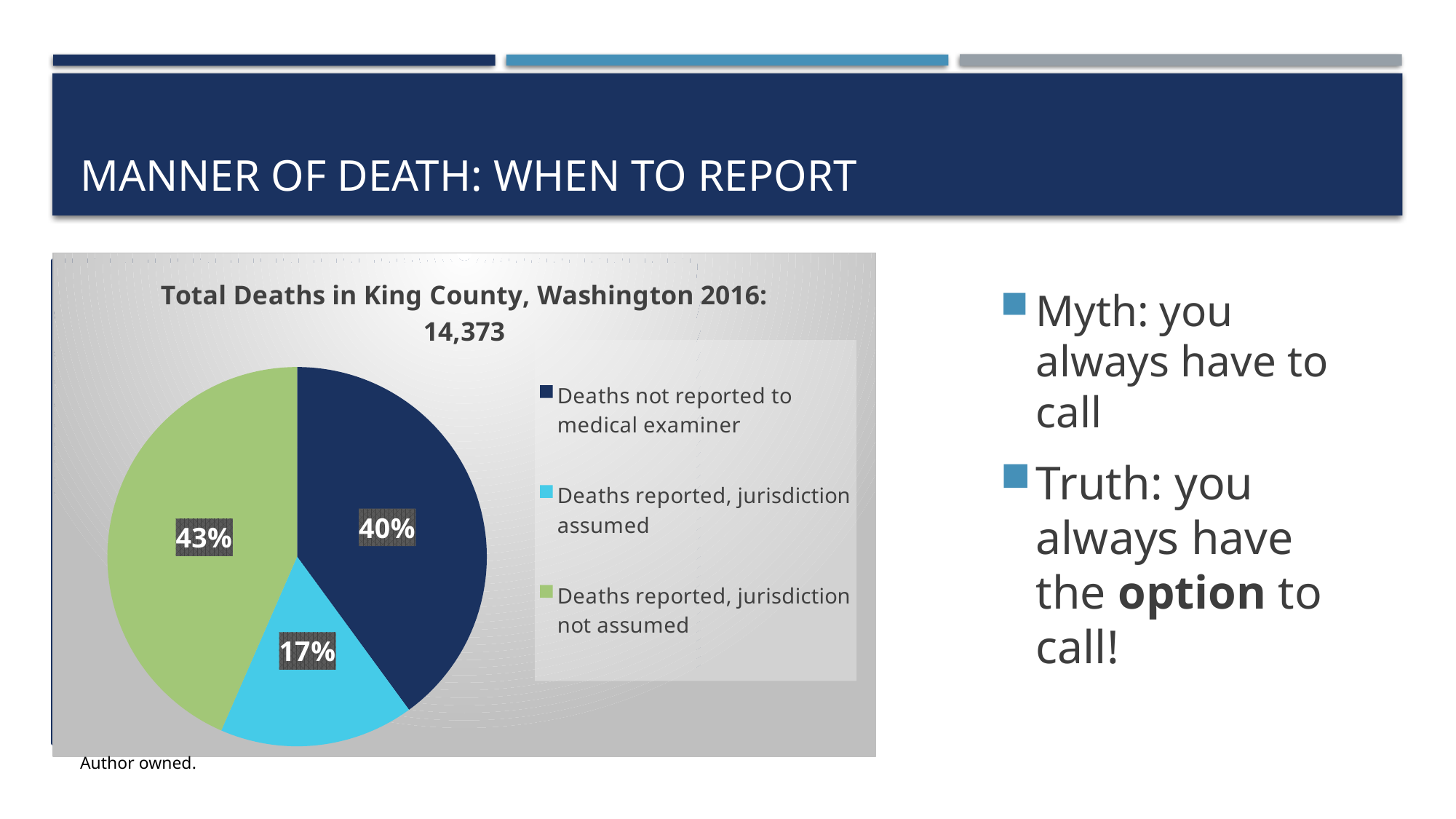

# Manner of Death: When to report
### Chart: Total Deaths in King County, Washington 2016: 14,373
| Category | |
|---|---|
| Deaths not reported to medical examiner | 5743.0 |
| Deaths reported, jurisdiction assumed | 2384.0 |
| Deaths reported, jurisdiction not assumed | 6246.0 |
Myth: you always have to call
Truth: you always have the option to call!
Author owned.

## Slide 42
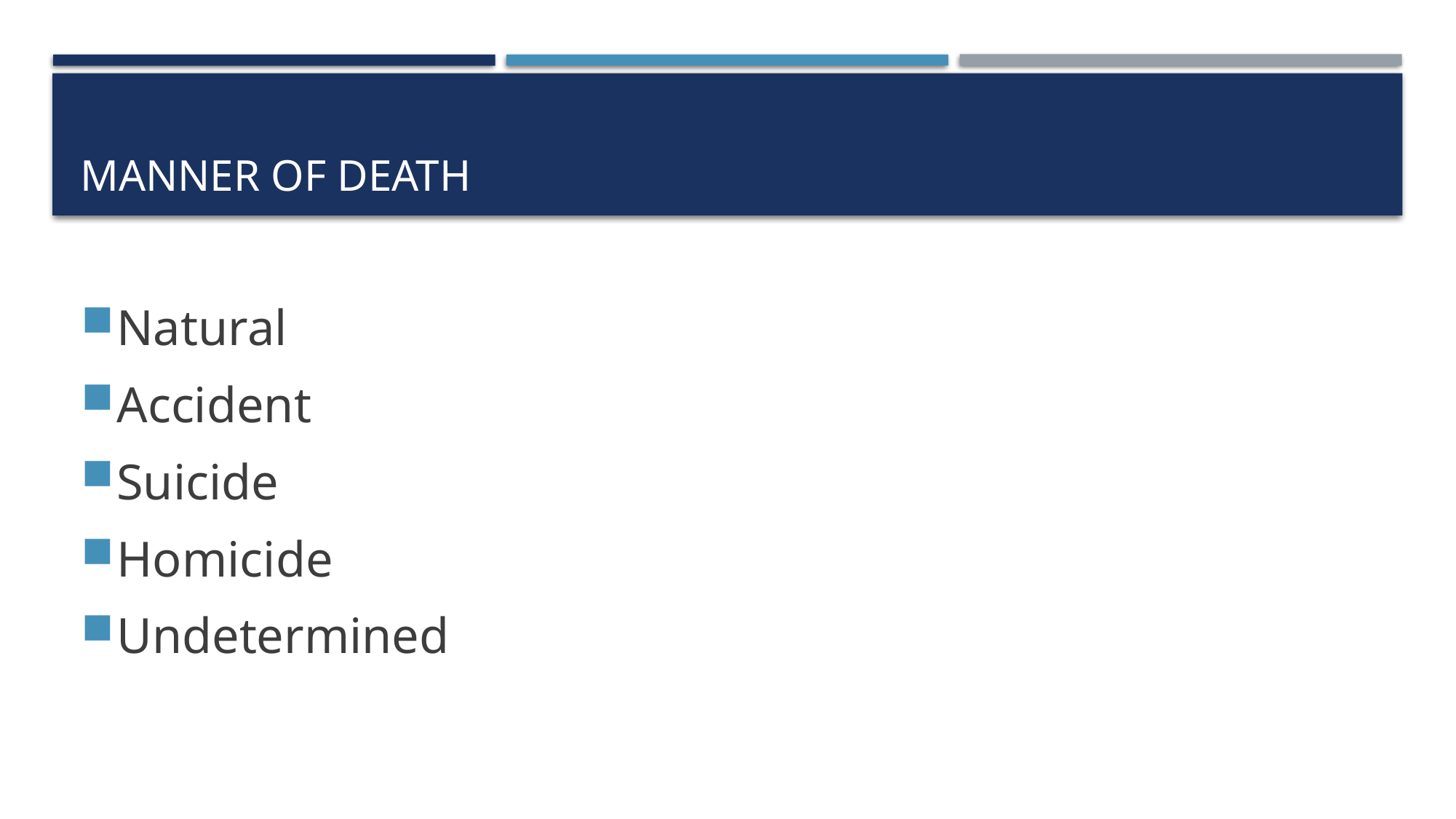

# Manner of Death
Natural
Accident
Suicide
Homicide
Undetermined

## Slide 43
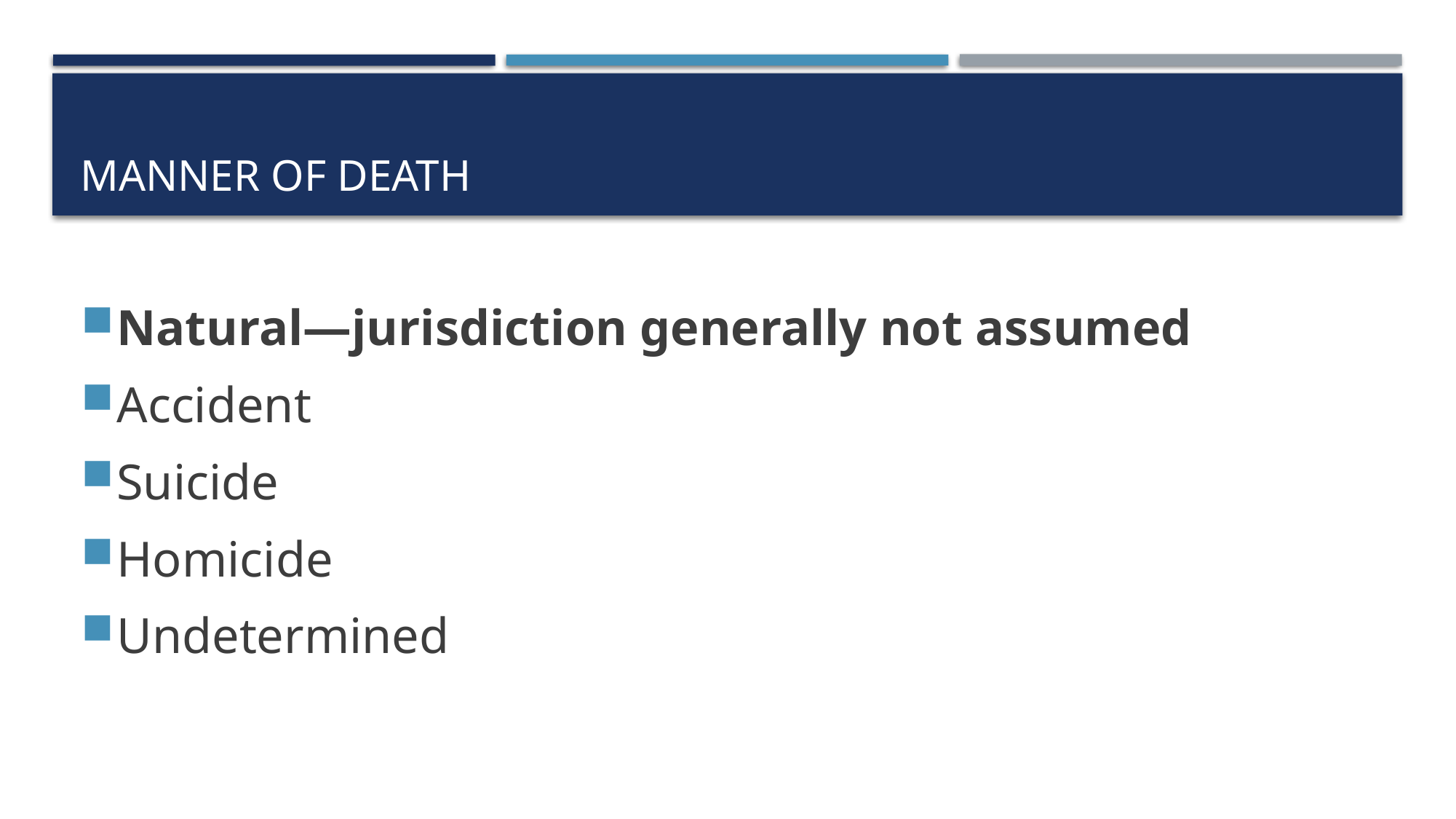

# Manner of Death
Natural—jurisdiction generally not assumed
Accident
Suicide
Homicide
Undetermined

## Slide 44
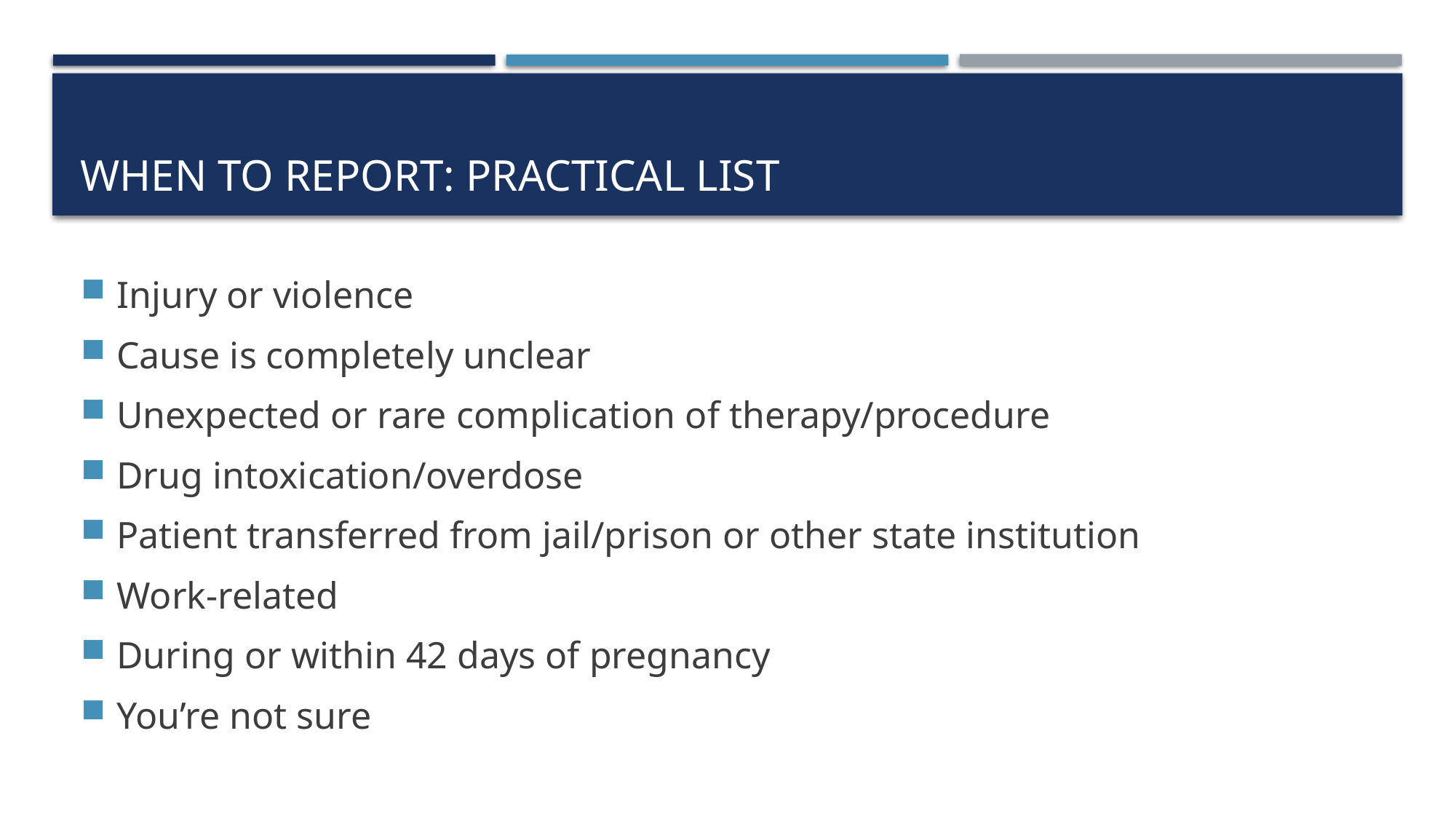

# When to report: practical list
Injury or violence
Cause is completely unclear
Unexpected or rare complication of therapy/procedure
Drug intoxication/overdose
Patient transferred from jail/prison or other state institution
Work-related
During or within 42 days of pregnancy
You’re not sure

## Slide 45
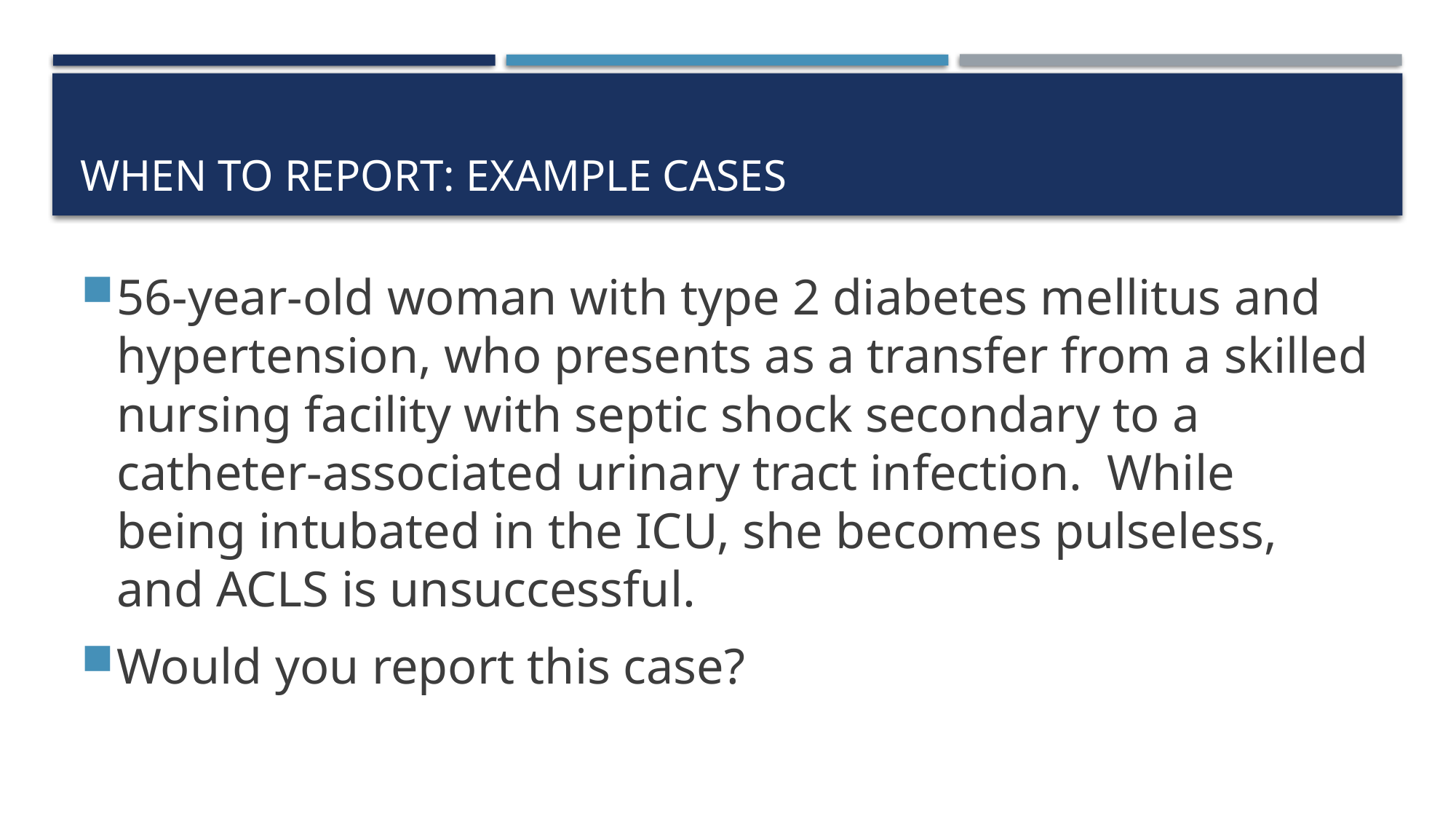

# When to report: example cases
56-year-old woman with type 2 diabetes mellitus and hypertension, who presents as a transfer from a skilled nursing facility with septic shock secondary to a catheter-associated urinary tract infection. While being intubated in the ICU, she becomes pulseless, and ACLS is unsuccessful.
Would you report this case?

## Slide 46
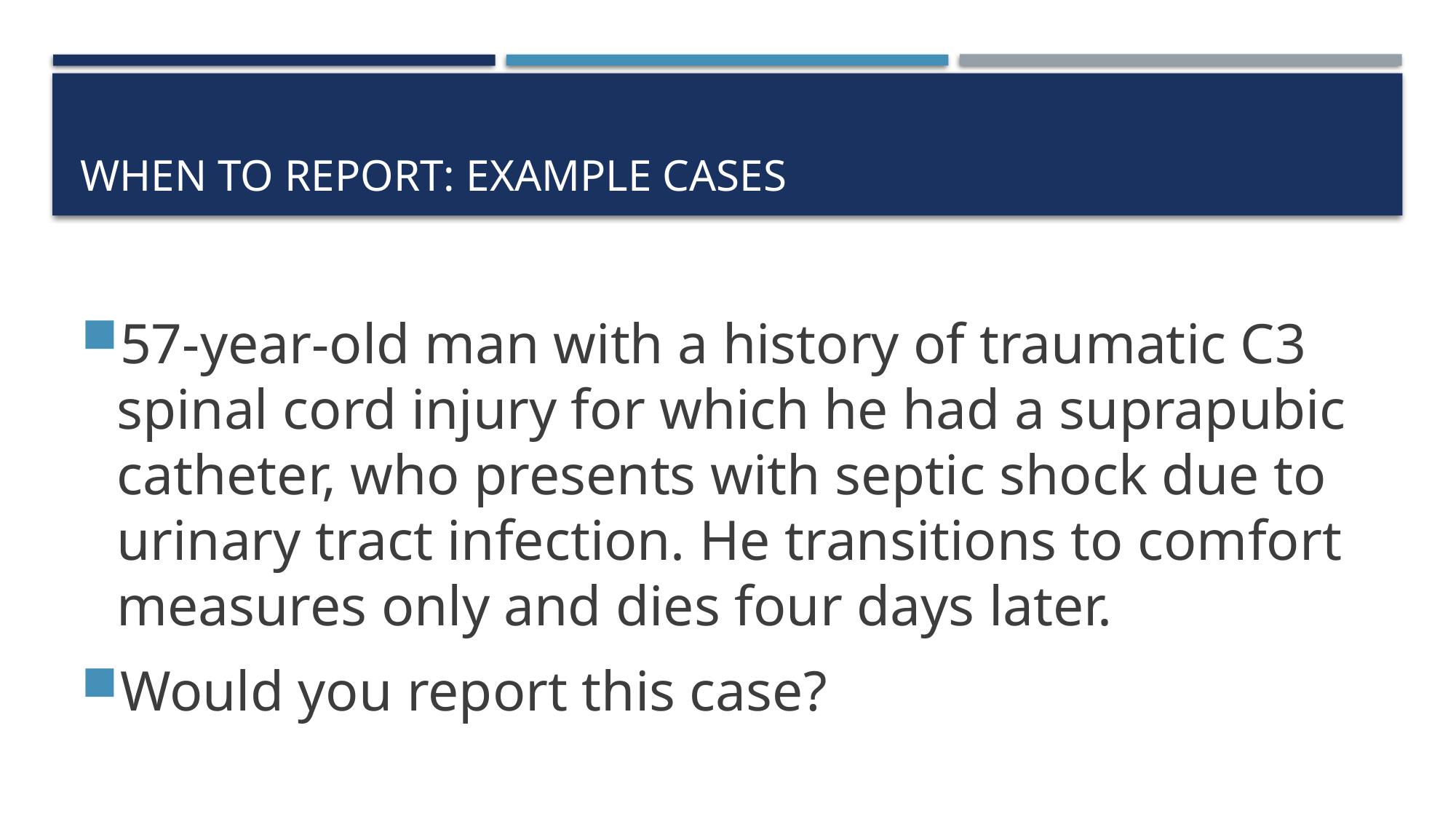

# When to report: example cases
57-year-old man with a history of traumatic C3 spinal cord injury for which he had a suprapubic catheter, who presents with septic shock due to urinary tract infection. He transitions to comfort measures only and dies four days later.
Would you report this case?

## Slide 47
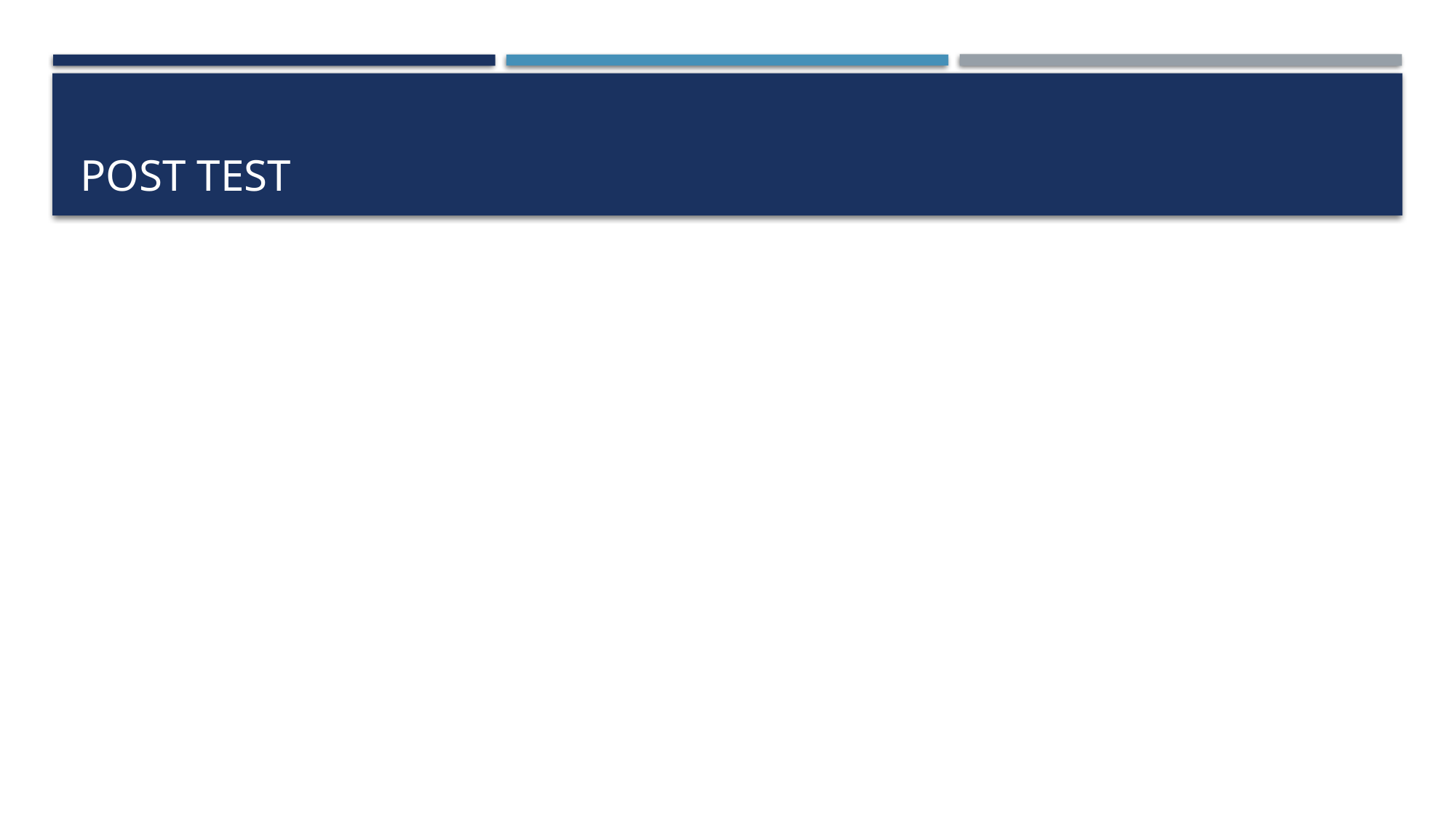

# POSt test

## Slide 48
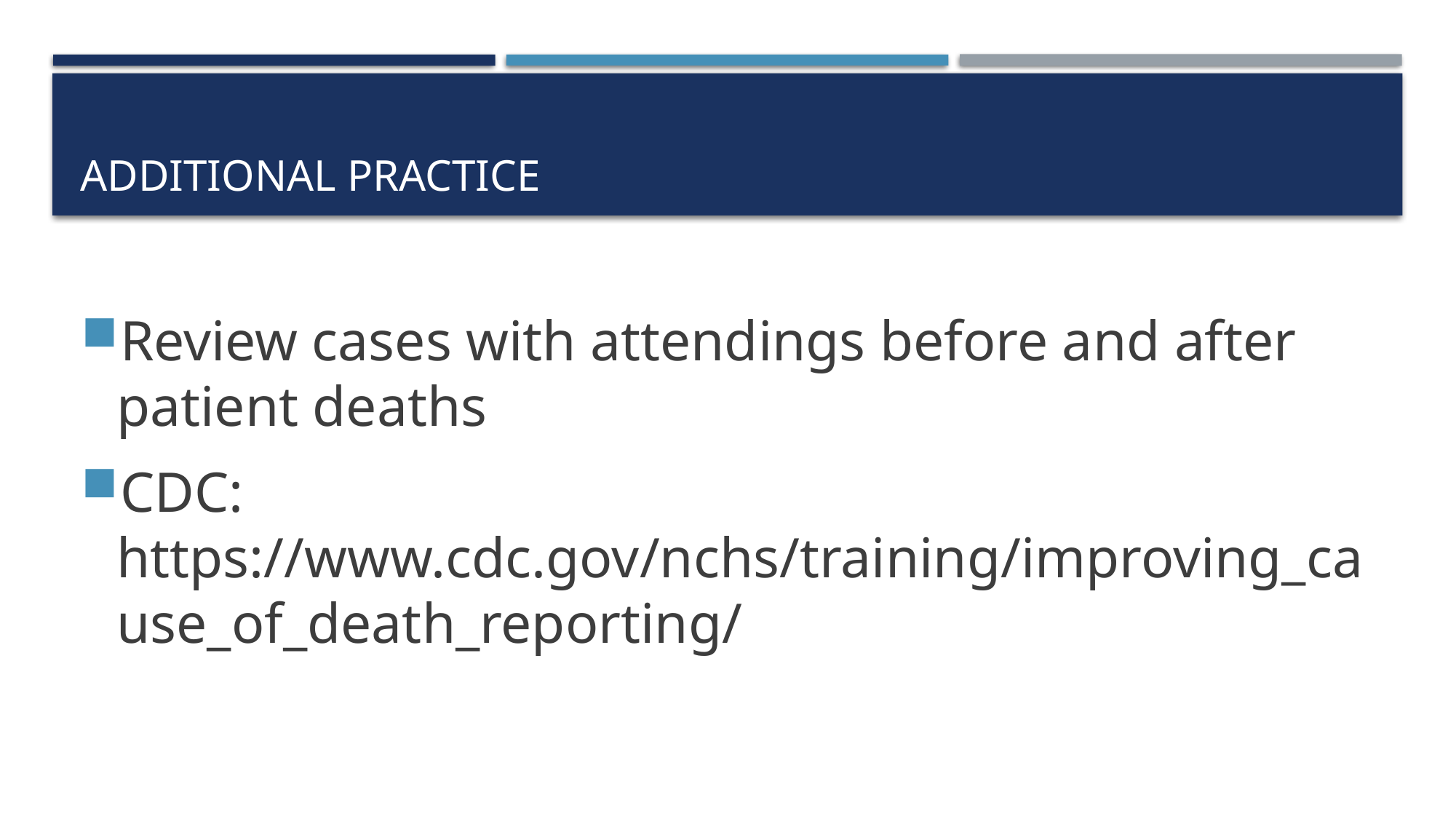

# Additional Practice
Review cases with attendings before and after patient deaths
CDC: https://www.cdc.gov/nchs/training/improving_cause_of_death_reporting/

## Slide 49
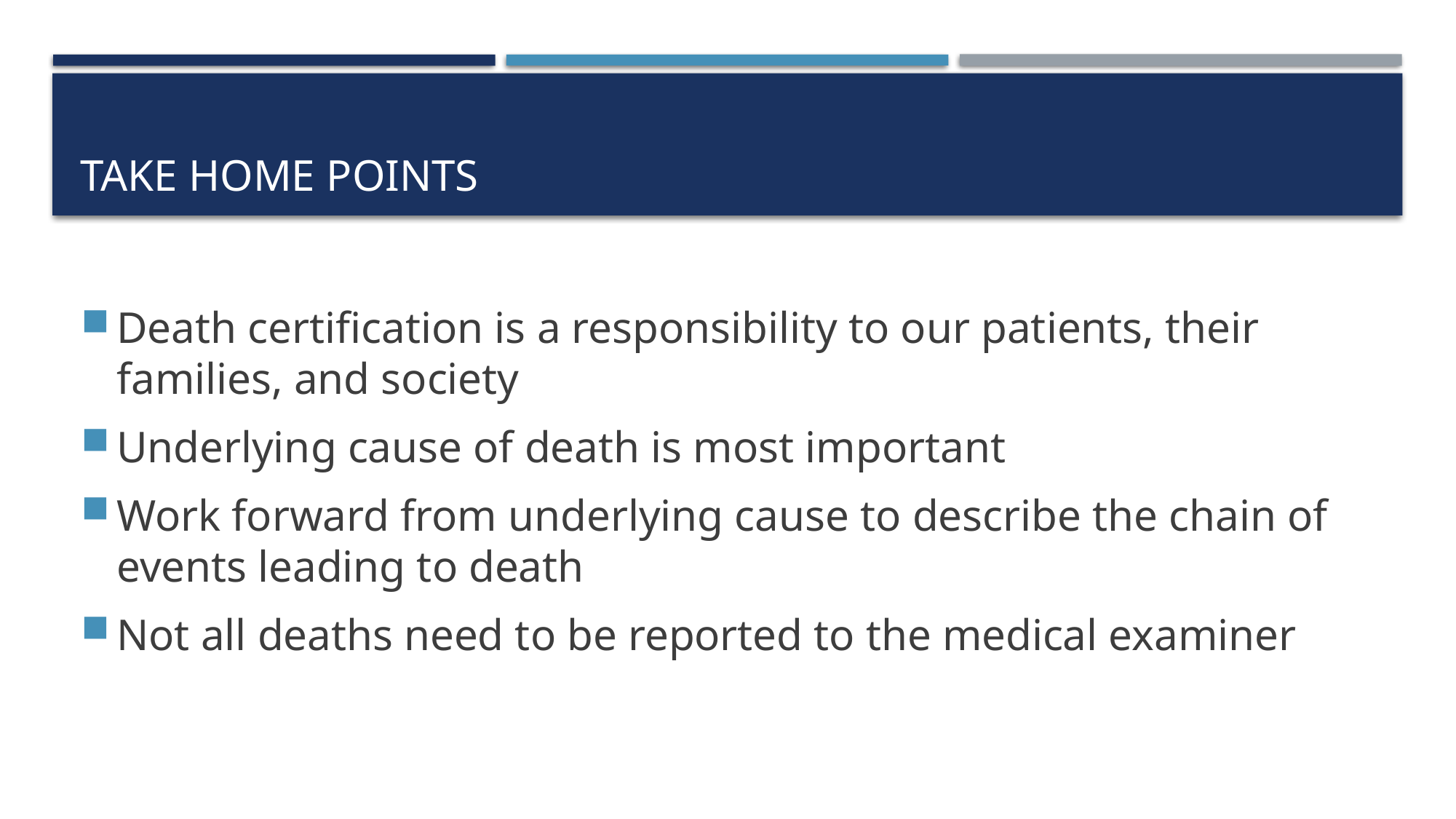

# Take Home Points
Death certification is a responsibility to our patients, their families, and society
Underlying cause of death is most important
Work forward from underlying cause to describe the chain of events leading to death
Not all deaths need to be reported to the medical examiner

## Slide 50
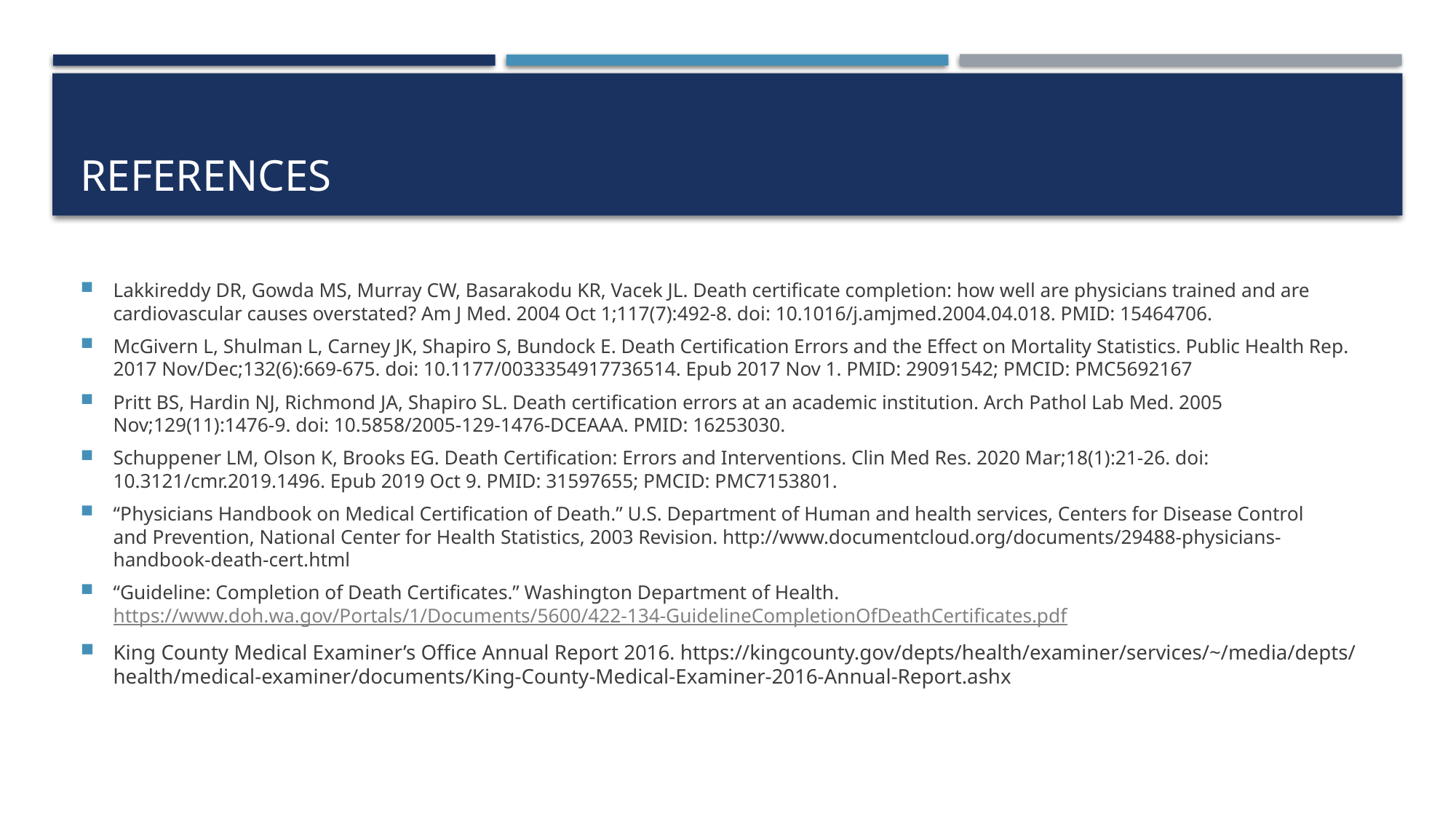

# References
Lakkireddy DR, Gowda MS, Murray CW, Basarakodu KR, Vacek JL. Death certificate completion: how well are physicians trained and are cardiovascular causes overstated? Am J Med. 2004 Oct 1;117(7):492-8. doi: 10.1016/j.amjmed.2004.04.018. PMID: 15464706.
McGivern L, Shulman L, Carney JK, Shapiro S, Bundock E. Death Certification Errors and the Effect on Mortality Statistics. Public Health Rep. 2017 Nov/Dec;132(6):669-675. doi: 10.1177/0033354917736514. Epub 2017 Nov 1. PMID: 29091542; PMCID: PMC5692167
Pritt BS, Hardin NJ, Richmond JA, Shapiro SL. Death certification errors at an academic institution. Arch Pathol Lab Med. 2005 Nov;129(11):1476-9. doi: 10.5858/2005-129-1476-DCEAAA. PMID: 16253030.
Schuppener LM, Olson K, Brooks EG. Death Certification: Errors and Interventions. Clin Med Res. 2020 Mar;18(1):21-26. doi: 10.3121/cmr.2019.1496. Epub 2019 Oct 9. PMID: 31597655; PMCID: PMC7153801.
“Physicians Handbook on Medical Certification of Death.” U.S. Department of Human and health services, Centers for Disease Control and Prevention, National Center for Health Statistics, 2003 Revision. http://www.documentcloud.org/documents/29488-physicians-handbook-death-cert.html
“Guideline: Completion of Death Certificates.” Washington Department of Health. https://www.doh.wa.gov/Portals/1/Documents/5600/422-134-GuidelineCompletionOfDeathCertificates.pdf
King County Medical Examiner’s Office Annual Report 2016. https://kingcounty.gov/depts/health/examiner/services/~/media/depts/health/medical-examiner/documents/King-County-Medical-Examiner-2016-Annual-Report.ashx
