# Supplementary material for: Death Certification: An Interactive Teaching Session
Source: MedEdPORTAL. 2023 Jan 17;19:11296. doi: 10.15766/mep_2374-8265.11296 (PMC9842806; doi:10.15766/mep_2374-8265.11296)
Supplement: Supplementary file 1 — Death Certification Interactive Session.pptxExample Cases.docxRubric for Grading Cases.docxTake-home Handout for Participants.docx [file mep_2374-8265.11296-s001.zip › B. Example Cases.docx]

Death Certification: An Interactive Teaching Session

Appendix B: Example Cases

These cases should be administered pre- and post-workshop, along with a template, to assess participants’ skills in filling out death certificates. Their responses will be graded using the template provided in Appendix C.

**Case 1: Case Description**

A 58-year-old-woman with hypertension, hyperlipidemia, and breast cancer with known peritoneal carcinomatosis is directly admitted from the oncology clinic for intractable pain. A CT scan of the abdomen and pelvis demonstrates her known metastatic disease. After talking with her primary oncologist and the Palliative Care team, she transitions to comfort measures only with the aim of going home on hospice care. Before she can be discharged, she dies in the hospital on a hydromorphone infusion.

**Case 1: Participant Template**

Immediate cause of death

Next oldest condition:

Next oldest condition:

Underlying cause of death:

Significant comorbidities contributing to death:

**Case 2: Case Description**

A 52-year-old man with chronic obstructive pulmonary disease is admitted to the ICU with hypoxemic respiratory failure due to COVID-19 for which he requires oxygen via high-flow nasal cannula. On hospital day 3, he develops worsening chest pain and hypoxemia, is found to have a new segmental pulmonary embolism and is started on anticoagulation. Despite this intervention, he develops progressive hypoxemia and suffers a cardiac arrest. After 30 minutes of advanced cardiac life support measures, return of spontaneous circulation is never achieved and he is declared dead.

**Case 2: Participant Template**

Immediate cause of death

Next oldest condition:

Next oldest condition:

Underlying cause of death:

Significant comorbidities contributing to death:
